# Phosphorus-Containing Silsesquioxane Derivatives as Additive or Reactive Components of Epoxy Resins

**DOI:** 10.3390/ma13235373

**Published:** 2020-11-26

**Authors:** Mariusz Szołyga, Michał Dutkiewicz, Marek Nowicki, Kamila Sałasińska, Maciej Celiński, Bogdan Marciniec

**Affiliations:** 1 Centre for Advanced Technologies, Adam Mickiewicz University in Poznan, Uniwersytetu Poznanskiego 10, 61-614 Poznan, Poland; markussqueeze@wp.pl (M.S.); midu@amu.edu.pl (M.D.); marek.nowicki@amu.edu.pl (M.N.); 2 Poznan Science and Technology Park, Adam Mickiewicz University Foundation, Rubiez 46, 61-612 Poznan, Poland; 3 Department of Chemical, Biological and Aerosol Hazards, Central Institute for Labour Protection - National Research Institute, Czerniakowska 16, 00-701 Warsaw, Poland; kasal@ciop.pl (K.S.); macel@ciop.pl (M.C.); 4 Faculty of Chemistry, Adam Mickiewicz University in Poznan, Uniwersytetu Poznanskiego 8, 61-614 Poznan, Poland

**Keywords:** silsesquioxane, phosphate, epoxy resin, flammability

## Abstract

Two phosphorus-containing cage-like silsesquioxane derivatives were synthesized as reactive or additive flame retardants for epoxy resin. The silsesquioxanes were obtained via an epoxide ring-opening reaction using a 10-hydroxy-9,10-dihydro-9-oxa-10-phosphaphenanthrene-10-oxide (DOPA). In one derivative containing in its structure 4 glycidoxypropyl and 4 phosphate groups, denoted as 4P4GS, only half of the epoxy rings was reacted with phosphate to obtain a reactive additive, while in the second derivative containing 8 phosphate groups, denoted as 8PS, all epoxy groups were converted, thus an additive modifier was obtained. The silsesquioxanes containing phosphorus atoms and the reactive phosphorus-free silsesquioxane derivative (octakis[(3-glycidoxypropyl)dimethylsiloxy]octasilsesquioxane (8GS)) were used to prepare hybrid materials based on epoxy resin. To compare the impact of the structure of silsesquioxane derivatives on the properties of hybrid materials, a number of samples containing 1, 5, and 10% of the modifiers making a series of epoxy materials containing additive or reactive modifiers, were obtained. The modified epoxies were studied using scanning electron microscopy and energy-dispersive X-ray spectroscopy (SEM-EDS), thermogravimetric analysis (TGA), differential scanning calorimetry (DSC), nanoindentation, water contact angle, and cone calorimetry tests to assess the effects of the modifier structure on the physicochemical properties of the investigated materials.

## 1. Introduction

Epoxy resins are one of the most important and broadly explored chemo setting polymers containing reactive epoxy (oxirane) functional groups that are receptive to attack by both electrophiles and nucleophiles. This attractive feature has permitted the development of a wide group of compounds applied as hardeners of different reactivity (i.a., aliphatic and aromatic amines, carboxylic anhydrides) to enable obtaining systems crosslinking in a wide temperature range and showing various physicochemical properties [1,2,3,4,5]. Epoxy resins are vital in a very wide range of commercially important applications, from high strength adhesives and special coatings to advanced composites. Profound interest in these materials results from the desirable properties of the hardened resin, including good adhesion to many surfaces, rather high hardness (often resulting from the type of modifier used), good resistance to external factors (chemical and environmental), high electrical resistance, and low shrinkage, as well as the possibility of the adaptation of the curing reaction to suit the processing requirements [6,7,8,9]. Unfortunately, one of the greatest limitations in the use of epoxy resins is their relatively high flammability and low thermal stability [10,11]. To circumvent these disadvantages, several flame-retardant additives have been applied in commercially available epoxy resins, including phosphorus compounds (e.g., triphenylphosphine, tris-β-chloroethyl phosphate), hydrates (e.g., alumina trihydrate), halogenated compounds (e.g., octabromobiphenyl, decabromobiphenyl, dechlorane) in different amounts ranging from 5 to 30%, often together with synergists like antimony oxide, zinc borate, or molybdic oxide [12,13,14].

Irrespective of the chemical structure and flame retardancy mechanism, flame retardants (FRs) can be divided into two groups: additive and reactive. The first of them is physically incorporated into the polymer matrix during its processing and does not react chemically with the polymer. The other ones are commonly introduced into the polymer at the stage of its synthesis (as co-monomers or polymer precursors) or in a polymer fictionalization process (polymer grafting). The latter FRs are chemically bonded to the modified polymers in this way [15]. The most commonly used FRs were based on halogenated organic derivatives. However, the presence of the halogen atoms (chlorine or bromine) in their structure caused the evolution of toxic and corrosive gases during the combustion of modified materials [16]. For this reason, the search for new FRs is still a top priority. Particularly promising are the materials based on silicon-, phosphorus-, and boron-containing compounds [17,18,19,20]. Moreover, besides the use of the abovementioned synergists, the literature also confirms the synergy of phosphorus and silicon compounds combined into one material [21,22]. Recently, the combination of additive phosphorus FRs and silsesquioxane derivatives have attracted the attention of several research groups [23,24,25,26,27].

In their work, Shi et al. describe the simultaneous modification of epoxy resin with octaaminophenyl silsesquioxanes and polyphosphazene, resulting in the formation of flame-retardant composites with significantly reduced peak heat release rate (pHRR) values [23]. A similar synergistic effect of silicon and phosphorus was reported by Cakmakci [24]. In his work, allylaminodiphenylphosphine oxide (APA) and octamercaptopropyl silsesquioxane were used to improve the flame retardancy of photocured epoxy acrylate-based coatings. The addition of APA itself influenced the reduction of the obtained epoxy acrylate composites’ flammability, while the addition of only silsesquioxane to the system increased their flammability [24]. In the work of Zhang et al., the pyrolysis and fire behavior of epoxy resin composites based on a silsesquioxane resin containing 9,10-dihydro-9-oxa-10-phosphaphenanthrene-10-oxide (DOPO-POSS) and diglycidyl ether of bisphenol A (DGEBA) were investigated. The results of the cone calorimetry measurements indicated that DOPO-POSS can significantly reduce the HRR of the investigated materials and enhance the formation of a char yield [25]. In their work, Zhang et al. described the effects of the phosphorus-based flame retardants applied with or without octaphenyl silsesquioxane (OPS) on the flammability of the prepared materials. The results of the LOI (limiting oxygen index) and UL-94 (flammability standard released by Underwriters Laboratories) tests showed that the flame retardancy of the samples is defined by the chemical structure of phosphorus FRs; however, the addition of OPS could enhance these effects [26]. In their work, Wang et al. also described the behavior of epoxy resin/silsesquioxane hybrids and phosphorus–silicon synergism, which improved the flame retardancy of the investigated systems. They have concluded that phosphorus promoted the char formation, while silicon protected the char against its thermal degradation [27].

However, cage-like silsesquioxanes containing other flame-retardant atoms, such as phosphorus or boron, incorporated into their structure have not been used and the relationship between the modifier structure and thermal stability or flammability of epoxy resins have not been evaluated as yet. Therefore, we decided to synthesize a family of phosphorus-containing cage-like silsesquioxane derivatives bearing different numbers of epoxy and phosphate groups to be used as reactive or additive epoxy resin modifiers. Subsequently, the proposed derivatives were used for epoxy resin modification, and the prepared materials were subjected to a series of tests to determine the influence of the structure of the proposed derivatives and the manner of their interaction with the polymer matrix on the properties of the obtained materials.

## 2. Materials and Methods

### 2.1. Materials

Octakis(hydridodimethylsiloxy)octasilsesquioxane was supplied by PIW Unisil Sp. z o.o., Tarnów, Poland, and 9,10-dihydro-9-oxa-10-phosphahenanthrene-10-oxide (DOPO) (>97%) was purchased from TCI Deutschland GmbH, Eschborn, Germany. Chloroform-d, allyl glycidyl ether (≥99%), and Karstedt catalyst (2% Pt in xylenes) were purchased from Sigma-Aldrich Sp. z o.o, Poznań, Poland. Acetone, tetrahydrofuran (THF), and toluene (all purities were suitable for analysis) were purchased from POCH, Gliwice, Poland. Hydrogen peroxide (30%) was purchased from ChemPur, Piekary Śląskie, Poland. Epoxy resin Epidian 652 (E652) and isophoronediamine (IDA) as a hardener were purchased from Ciech Resins, Nowa Sarzyna, Poland. All reagents were used without further purification.

### 2.2. Analytical Techniques

#### 2.2.1. Nuclear Magnetic Resonance Spectroscopy (NMR)

^1^H, ^13^C, ^29^Si, and ^31^P NMR spectra were recorded on a Varian XL 300, Zug, Switzerland, spectrometer at room temperature using CDCl_3_ as a solvent.

#### 2.2.2. Infrared Spectroscopy (FT-IR)

FT-IR spectra were recorded on a Bruker Tensor 27, Billerica, MA, USA, Fourier-transform spectrophotometer equipped with a single-reflection diamond ATR (Attenuated Total Reflectance) SPECAC Golden Gate, Orpington, GB module. In all cases, 16 scans were collected at a resolution of 2 cm^−1^ for the spectrum.

#### 2.2.3. Thermogravimetric Analysis (TGA)

Thermogravimetric analysis was carried out using a Mettler Toledo TGA/DSC 1 STAR^e^ System Columbus, OH, USA. The measurements were conducted in an air or N_2_ atmosphere at a flow rate of 60 mL min^−1^, in the temperature range from room temperature (RT) to 800 °C at a heating rate of 10 K min^−1^.

#### 2.2.4. Scanning Electron Microscope and Energy-Dispersive X-ray Spectroscopy (SEM, EDS)

The epoxy resin composites’ morphology was defined based on SEM images using an FEI Quanta 250 FEG, Hillsboro, Oregon, USA, field emission scanning electron microscope equipped with an EDAX, Mahwah, NJ, USA EDS (Energy Dispersive Spectroscopy) detector. The measurements were carried out in low- or high-vacuum mode. In the low-vacuum mode (50–100 Pa) with a 10 kV accelerating voltage, the signal was collected by an LFD (Low vacuum Secondary Electron) detector, while in the high-vacuum mode (2.5 × 10^−4^ Pa), the accelerating voltage was 5 kV and the signal was collected by an ETD (Everhart-Thornley Detector) FEI, Hillsboro, Oregon, USA, detector.

#### 2.2.5. Differential Scanning Calorimetry (DSC)

DSC measurements were carried out using a Mettler Toledo DSC-1, Columbus, OH, USA, differential scanning calorimeter. Each prepared epoxy resin composite sample was placed in 40 μL aluminum pans with a pierced lid and analyzed in a N_2_ atmosphere at a flow rate of 25 mL min^−1^ in the temperature range from 25 to 230 °C at a heating/cooling rate of 10 K min^−1^ to establish its glass transition temperature (T_g_). The T_g_ temperatures were assessed as the midpoints of the characteristic step transition in the baseline of the second cooling curves.

#### 2.2.6. Cone Calorimetry

The flammability of the prepared composites was assessed based on the results of cone calorimetry measurements. All samples were tested using Fire Testing Technology Ltd., East Grinstead, UK, apparatus according to ISO 5660 standard. Samples of dimensions of 100 × 100 × 6 mm were placed in aluminum foil and irradiated horizontally at a heat flux of 35 kW m^−2^. The pyrolysis products that were released during tests were spark ignited. The images of the residues after the flammability tests were collected using a Canon Inc. EOS 400 D Tokyo, Japan, digital camera. The FIGRA (fire growth rate) was calculated based on Equation (1):

(1) $$\mathrm{FIGRA}=\frac{\mathrm{pHRR}}{T_{\mathrm{pHRR}}},$$

where pHRR is the maximum heat release rate and T_pHRR_ is the time of the pHRR occurrences.

#### 2.2.7. Nanomechanical Analysis

The hardness and modulus of elasticity of the prepared samples were measured using a nanoindentation technique on an Agilent 5500 Santa Clara, CA, USA indenter with a high-precision DCM (Dynamic Contact Module) measuring head. A fused quartz sample was used for the nanoindenter calibration. The hardness and modulus of elasticity were calculated on the basis of results averaged from five measuring points for each sample.

#### 2.2.8. Static Water Contact Angle (WCA)

Static WCA values were measured using a Krüss GmbH DSA 100 Expert, Hamburg, Germany, drop shape analyzer equipped with a software-controlled (DSA4 2.0 Hamburg, Germany) motorized samples table, automatic dosing unit with automatically adjusted zoom, focus, illumination, and a camera with a 780 × 580 pix resolution. The drop profile was extracted using a circle-fitting model. Presented data are arithmetic means of measurements for five drops (5 µL volume) per sample.

### 2.3. Synthetic Procedures

#### 2.3.1. Synthesis of Octakis[(3-glycidoxypropyl)dimethylsiloxy]octasilsesquioxane (8GS)

8GS was synthesized according to the procedure described previously by authors via the hydrosilylation of allyl-glycidyl ether with octakisdimethylsiloxyoctasilsesquioxane catalyzed using Karstedt’s complex [19]. Dry toluene (400 mL) as a solvent, octakisdimethylsiloxyoctasilsesquioxane (40 g, 39.2 mmol), and allyl-glycidyl ether (44.8 mL, 376 mmol with 20% excess) were placed together in a reactor equipped with a thermometer, a condenser, and a magnetic bar. Next, Karstedt catalyst (171.2 mg, 2.35 × 10^−6^ mol Pt) was added at RT and the reaction mixture was heated to 110 °C and stirred for 8 h. After the reaction mixture had cooled down, the excess of olefin and the solvent were evaporated under vacuum to give a product as a viscous oil.

Spectroscopic analysis for 8GS: ^1^H NMR (CDCl_3_, δ (ppm)): 0.12 (OSiCH_3_); 0.58 (SiCH_2_); 1.60 (CH_2_); 2.57, 2.76 (CH_2_O); 3.11 (CHO); 3.36 (CH_2_O); 3.43, 3.66 (OCH_2_). ^13^C NMR (CDCl_3_, δ (ppm)): −0.30 (SiCH_3_); 13.75 (SiCH_2_); 23.26 (CH_2_); 44.38 (CH_2_O); 50.91 (CHO); 71.53 (OCH_2_); 74.17 (CH_2_O). ^29^Si NMR (CDCl_3_, δ (ppm)): 12.90 (OSi(CH_3_)_2_); −109.10 (SiOSi). FT-IR (ATR): 2998–2955, 2931, 2869, 1253, 1070, 902, 838 cm^−1^.

#### 2.3.2. Synthesis of 10-Hydroxy-9,10-dihydro-9-oxa-10-phosphaphenanthrene-10-oxide (DOPA)

DOPA was synthesized according to the procedure described elsewhere with a slight modification [28]. 9,10-Dihydro-9-oxa-10-phosphahenanthrene-10-oxide (50 g, 230 mmol) was placed in a three-necked round-bottom flask equipped with a thermometer, a condenser, and a magnetic bar, and hydrogen peroxide (150 mL, 1.9 mol) was added in one portion at room temperature. The temperature of the reaction mixture gradually rose to 100 °C and was allowed to drop down to 80 °C. The reaction mixture was stirred at this temperature overnight, cooled down to room temperature, and filtered. The obtained product was washed with acetone several times and dried overnight at 80 °C.

Spectroscopic analysis for DOPA: ^1^H NMR (CDCl_3_, δ (ppm)): 3.73 (PO–H); 7.28–8.10 (C–H_ar_). ^13^C NMR (CDCl_3_, δ (ppm)): 120.45–133.33 (C–H_ar_). ^31^P NMR (CDCl_3_, δ (ppm)): 14.03. FT-IR (ATR): 3100–3000, 1650–1450, 1210, 1000–920 cm^−1^. Substrate and product structures (DOPO and DOPA) as well as FT-IR and NMR spectra of DOPA presented in Figures S1–S5 are available in Supplementary Materials.

#### 2.3.3. Synthesis of Phosphorus-Containing Silsesquioxanes (4P4GS and 8PS)

A dry 250 mL three-necked flask equipped with a magnetic stirrer, thermometer, and reflux condenser was loaded with 10 g (5.2 mmol) of 8GS, 4.81 g (20.7 mmol) of DOPA (for the synthesis of 4P4GS and 9.62 g (41.4 mmol) for the synthesis of 8PS), and 100 mL of THF. The flask was then placed in an oil bath and the solution was heated to 55 °C and vigorously stirred. After about 3 h, the DOPA precipitate disappeared and the solution became transparent. The reaction was continued for another 20 h. After this time, the solution was transferred to a single-neck flask and the volatile components were evaporated on a vacuum evaporator. The product was then dried for another 48 h using a vacuum pump. The obtained products were in the form of a light orange, highly viscous oil. The reaction yield was quantitative (product weight for 4P4GS was 14.8 g and for 8PS was 19.6 g).

Spectroscopic analysis for 4P4GS: ^1^H NMR (CDCl_3_, δ (ppm)): 0.09, 0.10 (OSiCH_3_); 0.56 (SiCH_2_); 1.58 (CH_2_); 2.55, 2.73 (CH_2_O); 3.09 (CHO); 3.09–3.64 (CH_2_OH, CHOH, CH_2_O); 3.87 (HCOH); 4.12 (CH_2_OH); 7.20–7.90 (C–H_ar_). ^13^C NMR (CDCl_3_, δ (ppm)): −0.35 (SiCH_3_); 13.67 (SiCH_2_); 23.10 (CH_2_); 44.28 (CH_2_O); 50.88 (CHO); 59.18–74.14 (CH_2_O, CHOH, and CH_2_OH); 120.26–137.00 and 149.81 (C_ar_). ^29^Si NMR (CDCl_3_, δ (ppm)): 12.97 (OSi(CH_3_)_2_); −109.04 (SiOSi). ^31^P NMR (CDCl_3_, δ (ppm)): 11.28 (P–O–C^I^); 10.19 (P–O–C^II^). FT-IR (ATR): 3600–3200, 3100–3000, 2998, 2955, 2931, 2869, 1650–1450, 1253, 1210, 1070, 902, 838 cm^−1^.

Spectroscopic analysis for 8PS: ^1^H NMR (CDCl_3_, δ (ppm)): 0.08 (OSiCH_3_); 0.49 (SiCH_2_); 1.50 (CH_2_); 3.32 (CH_2_OH, CHOH, CH_2_O); 3.88 (HCOH); 4.14 (CH_2_OH); 7.19–7.88 (C–H_ar_). ^13^C NMR (CDCl_3_, δ (ppm)): −0.33 (SiCH_3_); 13.60 (SiCH_2_); 23.02 (CH_2_); 59.19 - 74.13 (CH_2_O, CHOH and CH_2_OH); 120.19–136.99 and 149.78 (C_ar_). ^29^Si NMR (CDCl_3_, δ (ppm)): 13.00 (OSi(CH_3_)_2_); −109.02 (SiOSi). ^31^P NMR (CDCl_3_, δ (ppm)): 11.14 (P–O–C^I^); 10.23 (P–O–C^II^). FT-IR (ATR): 3600–3200, 3100–3000, 2998, 2955, 2931, 2869, 1650–1450, 1253, 1210, 1070, 902, 838 cm^−1^.

#### 2.3.4. Epoxy Resin Samples Preparation

The appropriate amount (1, 5, or 10%) of the modifier (silsesquioxane 8GS, 4P4GS, or 8PS) was weighed into a plastic cup (amounts given in Table 1). Then, 40 g of epoxy resin (Epidian 652) was added to the cup and the contents were vigorously stirred by hand with a wooden stick for 10 min until the mixture was fully homogeneous. In a second cup, the appropriate amount of isophoronediamine (IDA) as a hardener (the amounts of hardener given in Table 1 were calculated such that it would react the epoxy groups present in both Epidian 652 and silsesquioxane) was weighed and poured into the cup already containing the resin with the modifier, and then the contents were vigorously stirred. Then, the resulting mixture was transferred to the hardener cup and stirred again, then poured into the resin cup again, and stirred (this procedure was repeated three times).

The mixture obtained in this way was divided into three parts placed in a 10 × 10 × 0.6 cm mold, on a glass plate (2.6 × 7.5 cm), and in a cylindrical form (diameter of 1.1 cm and height of 0.8 cm). Air bubbles were removed using a gas burner. The samples were left for 2 weeks at ambient temperature to fully cure.

## 3. Results and Discussion

### 3.1. Silsesquioxanes Synthesis and Characterization

The first step toward the implementation of the planned scope of research, assuming the assessment of the impact of the structure of silsesquioxane derivatives containing phosphate groups on the modified epoxy resins’ flammability, was the synthesis of appropriate derivatives.

**Scheme 1:**
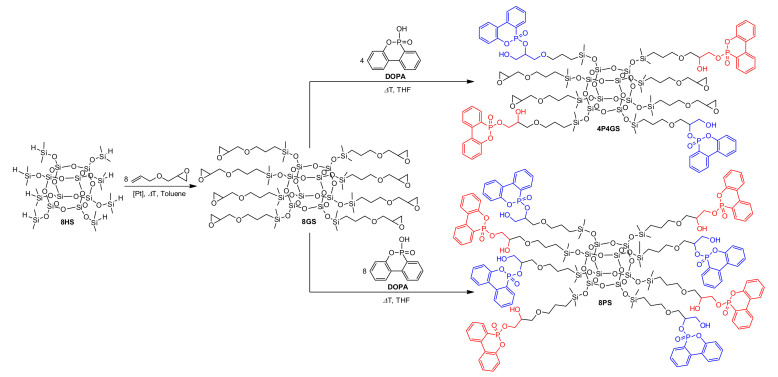
Synthesis of 8GS, 4P4GS, and 8PS silsesquioxane derivatives. 8HS: octakis(dimethylsiloksyhydrido)octasilsesquioxane, DOPA: 10-hydroxy-9,10-dihydro-9-oxa-10-phosphaphenanthrene-10-oxide,THF: tetrahydrofuran.

**Figure 1:**
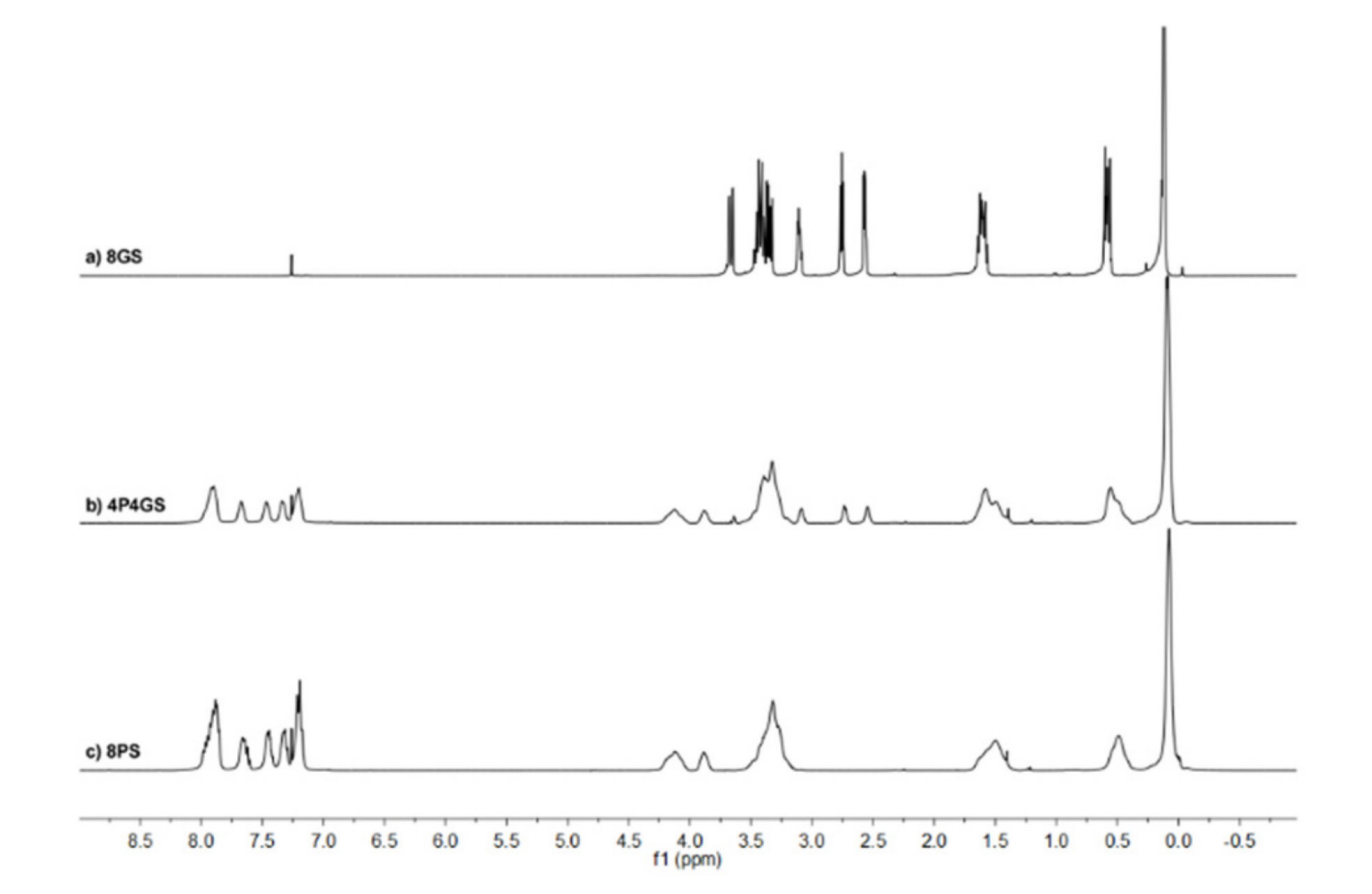
Stacked ^1^H nuclear magnetic resonance (NMR) spectra of the (**a**) 8GS, (**b**) 4P4GS, and (**c**) 8PS derivatives.

According to the presented Scheme 1, the synthesis was conducted in two steps. In the first one, the 8GS derivative containing eight glycidoxypropyl groups was obtained via hydrosilylation of the allyl-glycidyl ether with octakisdimethylsiloxyoctasilsesquioxane catalyzed using Karstedt’s complex, and after isolation, it was used as a substrate for the synthesis of two further derivatives. The synthesis of the 4P4GS derivative containing four glycidoxypropyl and four phosphate groups and the 8PS one containing eight phosphate groups only was carried out via an epoxy ring-opening reaction using the 8GS derivative and DOPA as substrates. By changing the stoichiometry of the reagents used, the silsesquioxane derivatives that could act as reactive flame retardants (4P4GS) chemically bonded to the structure of the modified epoxy resin or as an additive (8PS) that only physically mixed with the polymer matrix were prepared. Subsequently, the obtained derivatives were subjected to the spectroscopic analyses (^1^H-, ^13^C-, ^29^Si-, ^31^P-NMR, and FT-IR), which confirmed their structures. As can be observed in Figure 1, which shows the stacked ^1^H NMR, in the spectra of the 8GS, 4P4GS, and 8PS derivatives, the signals from the protons present in the Si(CH_3_)_2_ groups at 0.12 ppm and CH_2_ groups of alkyl chains at 0.58 and 1.6 ppm in the spectrum of the 8GS derivative were broadened and shifted upfield in the spectra of the 4P4GS and 8PS derivatives. The intensity of the signals from the protons of the ether and epoxy groups present in the spectrum of the 8GS derivative at 2.57, 2.76, 3.11, and 3.66 ppm decreased in the spectrum of the 4P4GS derivative and disappeared completely in the spectrum of the 8PS derivative. At the same time, the formation of new signals in the spectra of 4P4GS and 8PS derivatives at 3.88 and 4.13 ppm as a result of the epoxy ring-opening of 8GS derivatives is observed. Furthermore, the spectra of the 4P4GS and 8PS derivatives also showed groups of signals ranging from 7.1 to 8 ppm that are characteristic of the protons of aromatic groups derived from the DOPA derivative. NMR spectra of all discussed silsesquioxane derivatives are presented in Figures S6–S16 available in Supplementary Materials.

However, it needs to be highlighted that the structures of the obtained compounds (4P4GS and 8PS) presented in Scheme 1 are a kind of simplification. The applied method for the synthesis of the 4P4GS derivative containing phosphate and glycidoxypropyl groups in its structure leads, in fact, to the formation of a mixture of compounds with different numbers of the discussed groups and their different arrangements around the inorganic silsesquioxane core, which was demonstrated in the earlier works of our research group based on the results of the MALDI (Assisted Laser Desorption and Ionization (UltrafleXtreme, Bruker, Billerica, MA, USA)) analysis of silsesquioxane derivatives containing two types of functional groups in their structures [29,30]. However, statistically, the ratio of epoxy to phosphate groups was 4:4. In the case of the 8PS derivative, the presence of a mixture of compounds should also be assumed, which resulted from the possibility of a different substitution of DOPA to epoxy groups, as shown in Scheme 1.

**Figure 2:**
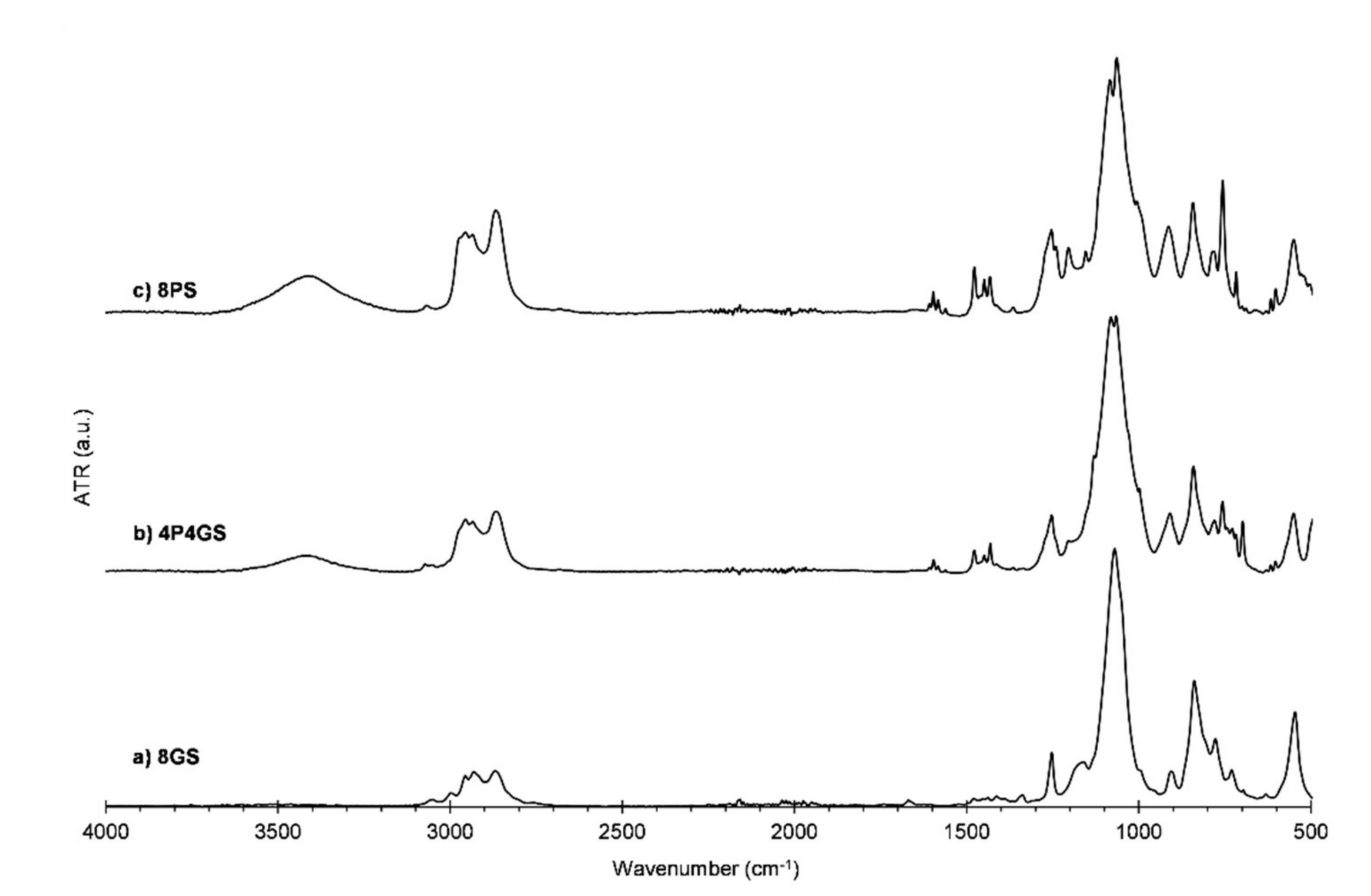
Stacked Fourier-transform infrared spectroscopy (FT-IR) spectra of the (**a**) 8GS, (**b**) 4P4GS, and (**c**) 8PS derivatives.

Furthermore, the results of the FT-IR analysis of the obtained derivatives presented in Figure 2 confirmed the structures of the obtained derivatives. This was evidenced by the appearance of bands that are characteristic for vibrations of OH groups at 3400 cm^−1^ in the spectra of 4P4GS and 8PS derivatives, groups of bands in the range of 1600–1400 cm^−1^, and bands with a maximum at 1070 (split), 905, and 754 cm^−1^, which were assigned to the stretching vibrations of C–H and C=C bonds of aromatic rings, stretching vibrations of P–C (aromatic) bonds, and asymmetric and symmetric stretching vibrations of P–O–C (aliphatic) bonds, respectively.

The obtained silsesquioxane derivatives were then used to prepare samples of modified epoxy resins based on the isophoronediamine-cured Epidian 652 resin, as described in Section 2.3.4. The resin samples prepared in this way were subjected to further tests to assess the effect of the structure and amount of used modifiers on the properties of the produced resins.

### 3.2. Scanning Electron Microscopy and Energy-Dispersive X-Ray Spectroscopy Measurements

**Figure 3:**
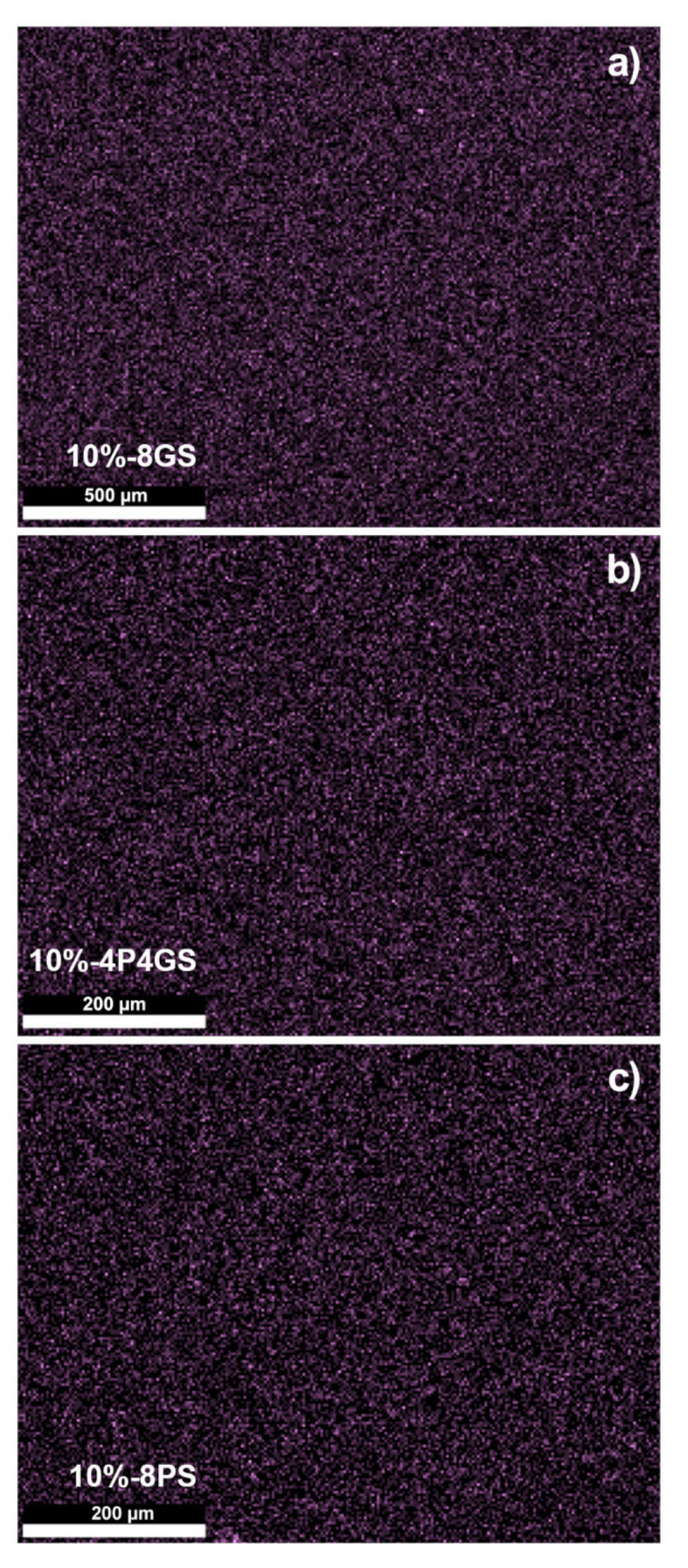
Scanning electron microscopy and energy-dispersive X-ray spectroscopy (SEM-EDS) silicon distribution maps of the modified epoxy resins containing 10% of (**a**) 8GS, (**b**) 4P4GS, and (**c**) 8PS derivatives.

The SEM technique combined with EDS was employed for the evaluation of the amount and the distribution of silsesquioxane modifiers used in the epoxy matrix based on the silicon and phosphorous atoms’ concentrations. Analysis of the recorded images confirmed the uniform distribution of modifiers in the polymer matrix, irrespective of the type and amount of the modifier used, as shown in the exemplary distribution maps of silicon atoms in the samples 10%-8GS, 10%-4P4GS, and 10%-8PS, which contained the highest amounts of each modifier used, as presented in Figure 3.

The results of the silicon and phosphorus concentration measurements that are presented in Table 2 confirm the assumed compositions of the prepared resins.

The SEM-EDS micrographs confirming the uniform distribution of the silicon and phosphorous atoms in the other samples can be found in the Supplementary Materials (Figures S17–S46 and Tables S1–S10).

### 3.3. Surface Properties

To assess the effect of the type and amount of the modifier on the surface properties of the prepared samples, the hydrophobicity of their surfaces was determined based on the static WCA. The WCA values obtained for all samples are summarized in Table 3. According to the results, for all samples except 10%-8PS, the WCA values were slightly increased, from 77° for the pure epoxy resin sample to about 80–83°. The decrease in WCA to 50° observed for the 10%-8PS sample was caused by the highest content of phosphate groups, commonly known as effective surfactants. The obtained results showed that irrespective of the type and amount of the modifier used, their static water contact angles remained below 90°, which, in light of the adopted definitions, classifies them as hydrophilic materials [31]. 

### 3.4. Mechanical Properties

The effect of the type and amount of the silsesquioxane modifier on the modulus of elasticity and hardness of the prepared epoxies were evaluated using nanoindentation, in which the measured parameters were determined based on the continuously recorded indenter load on the sample and its displacement. The recorded load–displacement curves were then analyzed to determine the mentioned parameters (elasticity modulus and hardness) [32]. The main advantage of the discussed analytical technique is the possibility of obtaining information on the basic mechanical properties of the tested materials based on the analysis of samples with limited dimensions (coatings, thin layers) or an increased heterogeneity, such as polymer nanocomposites, including those based on epoxy resins [33,34,35,36].

The results of the measurements carried out at room temperature are presented in Table 4. The presented mechanical parameters are the average values from 16 independent measurements made for each sample.

The results revealed that the type and amount of silsesquioxane used for the preparation of the modified epoxy resins had a marginal influence on the measured mechanical parameters. Nevertheless, it can be observed that the modulus of elasticity and hardness of all the prepared modified epoxies were higher than those measured for the reference sample. The lack of differences in hardness and modulus of elasticity between the samples modified with the silsesquioxane derivatives that were capable of chemical incorporation into the resin structure and those without epoxy groups suggests that in the tested materials, the additives used played only the role of a nanofiller and the increase in crosslinking density did not affect the mechanical properties of the produced materials. It should also be noted that the low standard deviation values of the measured parameters (not exceeding 3%) proved the high homogeneity of prepared resins, which is consistent with the results of the SEM-EDS analysis.

### 3.5. TGA and DSC Measurements

The samples of epoxy resins modified with 1, 5, and 10 wt.% of all silsesquioxane derivatives were subsequently subjected to TG and DSC analyses to evaluate the effects of the chemical structure of the studied silsesquioxane derivatives and their amount on the thermal properties of investigated hybrid epoxies. The obtained results revealed some quite well-pronounced differences in the TG and DTG (derivative thermogravimetric) curves, confirming the influence of the applied silsesquioxane derivatives on the epoxy resin thermal behavior and the relationship of this behavior to the chemical structures of the derivatives. As the observed changes were the most pronounced for the samples containing the highest amount of modifiers used, only the TG and DTG curves for the samples containing 10% by weight of 8GS, 4P4GS, and 8PS derivatives are presented. The remaining curves for samples containing 1 and 5% of the modifiers are presented in Figures S47–S52 available in Supplementary Materials.

**Figure 4:**
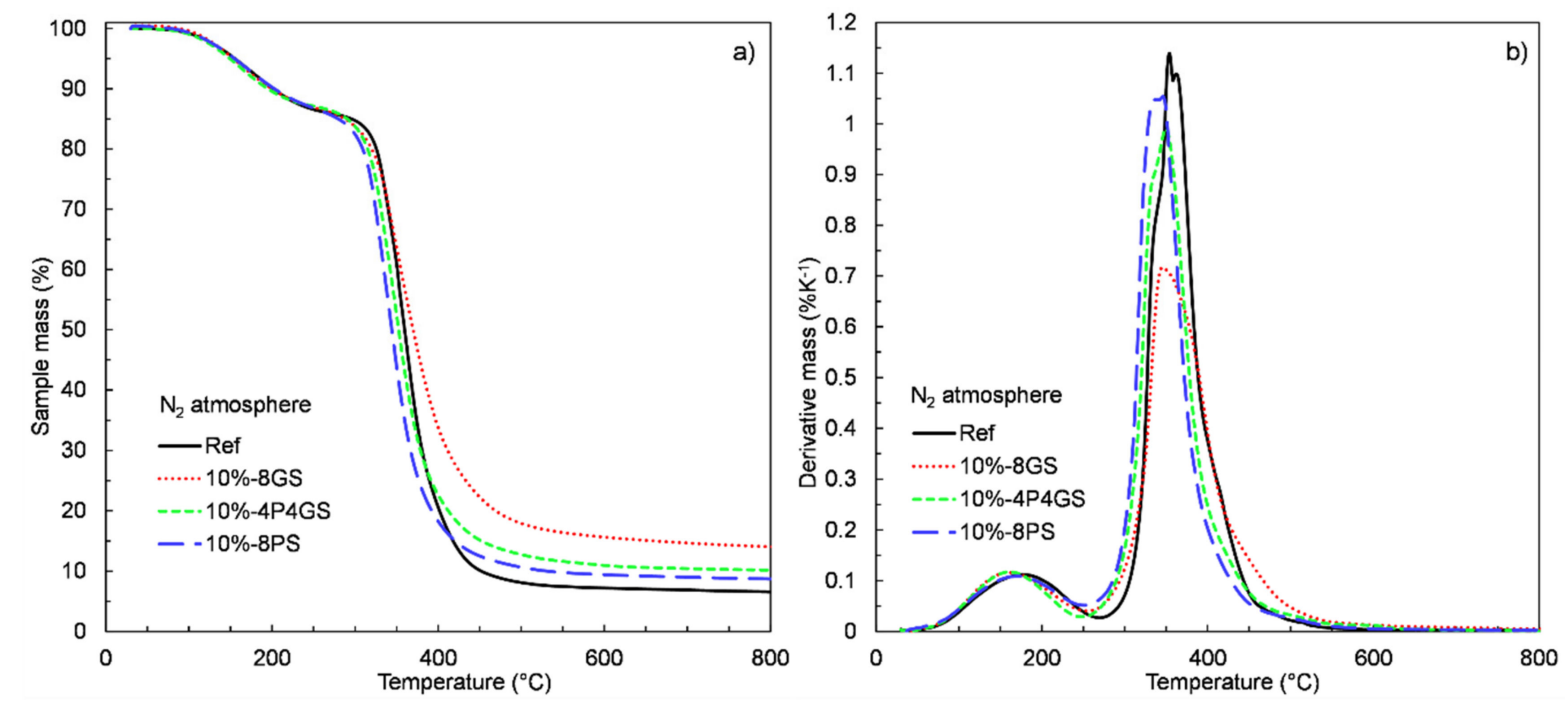
Thermogravimetric TG (**a**) and derivative thermogravimetric DTG (**b**) plots for epoxy resins modified with 10% of the 8GS, 4P4GS, and 8PS derivatives in the N_2_ atmosphere.

The results of the TG and DTG analyses in the N_2_ atmosphere shown in Figure 4 revealed that all investigated samples decomposed in two distinct steps, taking place in the ranges from 40–270 and 270–600 °C. The first of them was most probably the result of the evaporation of benzyl alcohol present in the IDA hardener in the amount of approximately 20 wt.% and trapped in the prepared samples during the curing process, while the second one was the result of the thermal decomposition of the polymeric network.

As mentioned above, the influence of a 1% addition of all the tested silsesquioxane derivatives on the thermal properties of the obtained materials was marginal. More pronounced effects of the applied modifiers were observed for the 5 and 10% additions. The addition of 5% of the 8GS derivative caused a slight increase in the thermal stability of the epoxy resin, but increasing its amount to 10% by weight caused a slight decrease. Furthermore, in the case of samples containing 5 and 10% of the 8GS derivative, a decrease in the rate of the decomposition process was observed, which was manifested as a decrease in the maxima of the DTG curves and an increase in the residue at 800 °C. The effect of a decrease of the thermal stability was also observed in the case of samples containing 5 and 10% of the 4P4GS derivative, and especially the 8PS one. This was evidenced by the shift of the peaks of the second decomposition stage in their DTG curves toward lower temperatures with a simultaneous slight decrease in their height.

The observed effects were directly related to the changes in the structure of the applied modifiers and resulted from the decreased number of reactive epoxy groups present in the applied modifier molecules (from 8 to 4 and 0), causing a gradual decrease in the samples’ crosslinking density and an increase in the content of phosphate groups, from 0 to 4 and 8. The detailed results of TG and DTG analyses carried out in the N_2_ atmosphere are summarized in Table 5 and Table 6.

**Figure 5:**
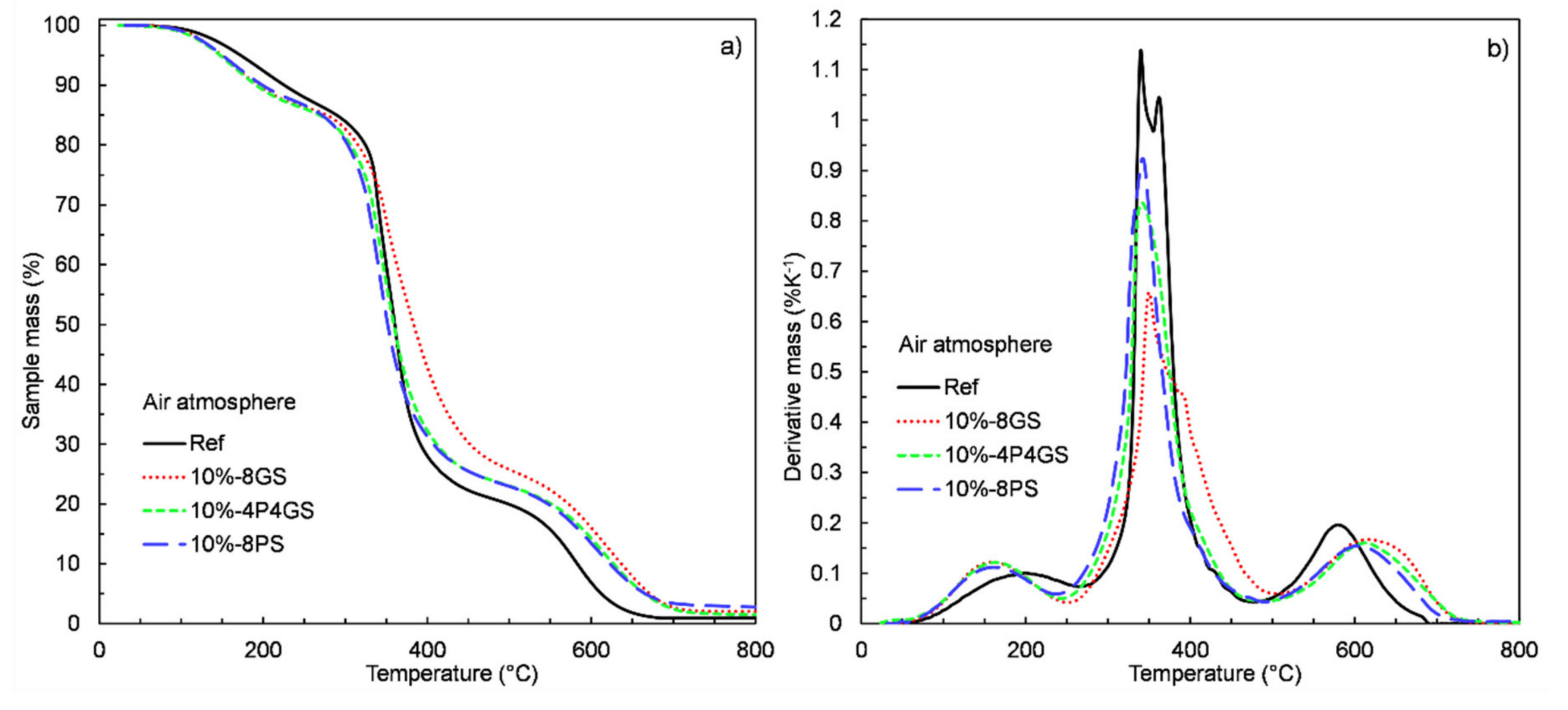
TG (**a**) and DTG (**b**) plots for the epoxy resins modified with 10% of 8GS, 4P4GS, and 8PS derivative in the air atmosphere.

The differences in the thermal behavior of the discussed resins are even more pronounced in the case of the TG and DTG curves for samples measured in the air atmosphere shown in Figure 5.

While the bimodal decomposition process was observed in the experiments carried out in the N_2_ atmosphere, an additional decomposition step in the range of 470–750°C (depending on the amount and type of modifier used) was observed for all samples measured in the air atmosphere, making the process trimodal. Besides the previously discussed effects of the decrease of the thermal stability of the tested samples and the lowering of their decomposition rate, in the case of experiments conducted in the air atmosphere, the influence of the modifiers used on shifting the third decomposition step of modified resins toward higher temperatures was also observed. This proved that the presence of the discussed derivatives could help to preserve the carbonized protective layer that formed in the second decomposition stage and could protect the materials against high temperatures and restrict the combustion process. The results of TG and DTG analyses for all samples measured in the air atmosphere are summarized in Table 7 and Table 8.

Although the results of TG analysis confirmed the effects of the chemical structure and the amount of the discussed silsesquioxanes on the thermal decomposition kinetics of the modified epoxy resins, it is hard to compare the obtained results with those previously reported by other research groups as they are new compounds and have never been tested before, as well as due to the number of factors affecting the thermal stability of modified epoxy composites, such as the type of epoxy resin and hardener used; the amount, type, and the way it interacts with the polymer matrix; the manner of composite preparation (blending or curing process). Nevertheless, by comparing the obtained results to the exemplary research on epoxy resins modified with octasilsesquioxane derivatives with phosphate groups of a similar structure obtained by Liu et al. or Zheng et al., certain regularities can be found [37,38,39,40]. The results of the TG analysis presented by the abovementioned research groups, as in the case of the discussed derivatives and the materials obtained with their use, revealed that the addition of DOPO-functionalized silsesquioxane derivatives, especially introduced in an amount exceeding 3.5%, slightly reduces the temperature of the beginning of the decomposition process of the tested samples, decreases the rate of the first decomposition step and, in the case of measurements carried out in an air atmosphere, increases the temperature of the maximum decomposition rate at its last stage. The addition of DOPO-functionalized octasilsesquioxane derivatives to epoxy resins also causes a slight increase in char yield at 700–800 °C.

Despite the presence of certain analogies, it should be emphasized that the compared studies were conducted with the use of slightly different epoxy resins, other curing agents, and the prepared samples were hardened at different temperatures. Moreover, it should be noted that the derivatives used by Liu et al. and Zheng et al. did not have reactive epoxy groups in their structure that would enable their chemical incorporation into the material’s structure. As mentioned above, all these factors make a more precise comparison of the thermal properties of the discussed materials very difficult.

The DSC analysis also revealed slight changes in the course of DSC curves (see Figures S53–S56 in the Supplementary Materials), which was dependent on the type and amount of the modifier. Only the glass transition (T_g_) manifested as the characteristic, reversible step transition in the heat flow was observed in this case. The glass transition temperatures, determined as the midpoints of the second cooling curves, are collected in Table 9. It can be seen that the addition of 1% of reactive 8GS or 4P4GS caused a slight increase in the T_g_ temperature compared to that of the reference sample, while for the sample containing 1% of 8PS, the T_g_ temperature was almost unchanged. The addition of 10% of all modifiers resulted in a minor decrease in the T_g_ temperature. The most significant decrease (by more than 4 °C) was observed for the sample containing 10% of the 8PS derivative with no reactive epoxy groups. The DSC analysis confirmed the effect of the chemical structure and amount of the silsesquioxanes used on the thermal properties of the modified epoxy resins.

### 3.6. Flammability

**Figure 6:**
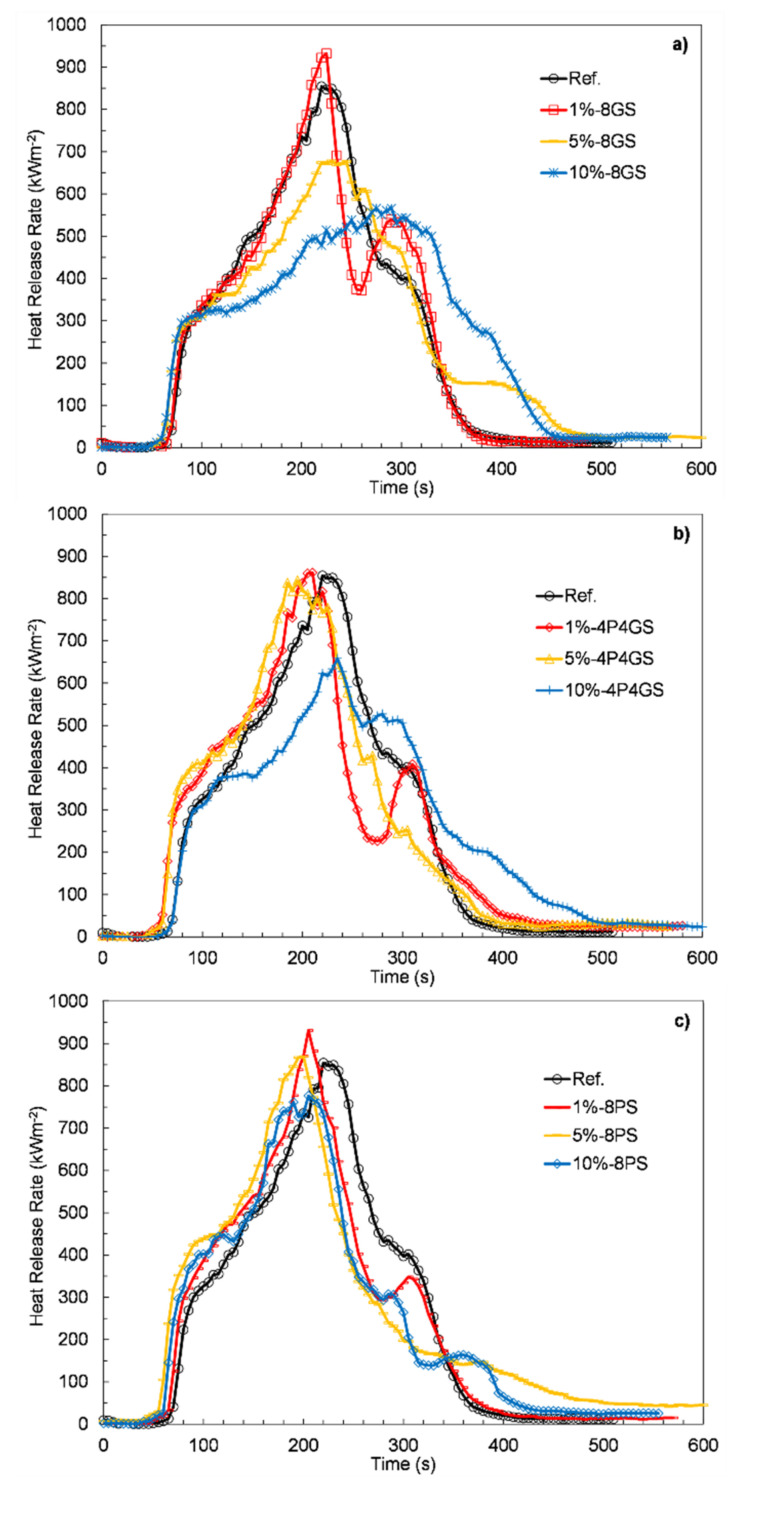
Heat release rate (HRR) curves of the epoxy resin samples modified with (**a**) 8GS, (**b**) 4P4GS, and (**c**) 8PS silsesquioxane.

The response of the formulations studied to a simulated fire scenario was evaluated using the measurements taken with a cone calorimeter (CC) [41]. The heat release rate (HRR) curves are presented in Figure 6, while the values of the key parameters for the assessment of the flammability of the tested materials are summarized in Table 10.

**Figure 7:**
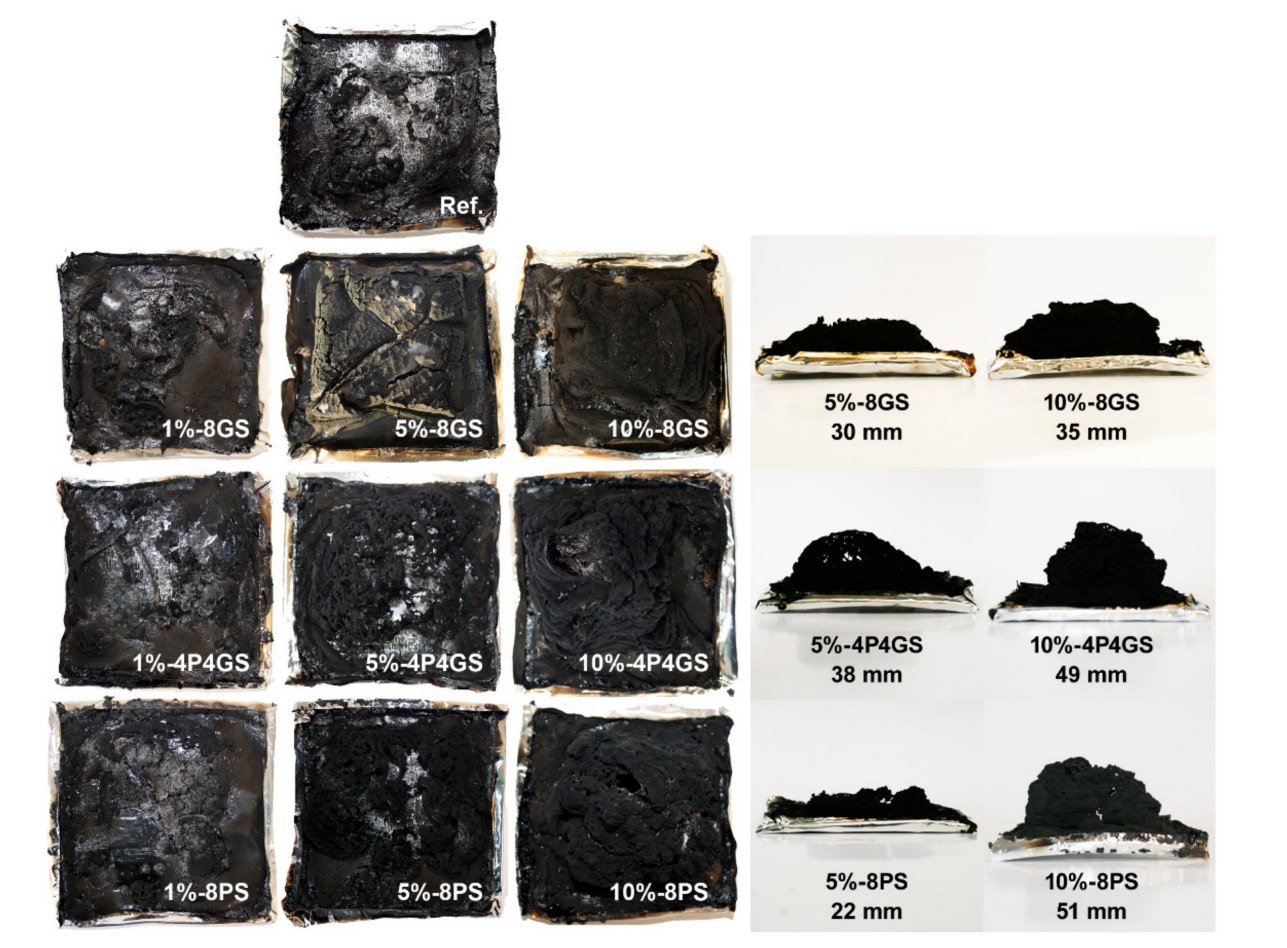
Images of the residue after the cone calorimetry tests (top and side views).

It was found that all samples were ignited after 51–67 s, and the time to ignition (TTI) values did not depend on the presence or amount of the investigated flame retardants (Table 10). As can be seen from Figure 6, the reference unmodified epoxy resin (Ref.) burned intensively with a pHRR of 855 kW m^−2^ and left a very small residue (Figure 7).

The addition of 1 wt.% of the functionalized silsesquioxanes did not reduce the HRR; on the contrary, the pHRR values increased, and no change in the amount of residue was observed. The incorporation of 5 wt.% of the silsesquioxanes led to the formation of carbonaceous char; however, only when 8GS was added, a significant reduction in pHRR, by approximately 21%, was noted. The image of the 5%-8GS sample after the cone calorimetry test (Figure 7) shows the swollen structures with numerous cracks, while the images of the 5%-4P4GS and 5%-8PS reveal a discontinuous mesh-like structure (Figure 7). The lowest pHRR, one-third lower than that of the unmodified resin, was obtained for sample 10%-8GS. Only for this material, the char in the form of compact and severely swollen structure, covering most of the sample, was observed. The formation of swollen char prevents heat transmission and enhances the flame retardancy of polymeric materials [42]. The maximum average rate of the heat emission (MARHE), which corresponds to the peak of the cumulative heat emission divided by time, was determined to evaluate the fire development rate.

The values of the MAHRE that were determined for the samples containing 1–10 wt.% of the 8GS derivative were lower than that of the reference sample, while for the other samples, it was necessary to add at least 10 wt.% of the modifier to achieve a similar result. Another parameter of great importance that is used to assess the performance of flame retardants is the FIGRA. The values of this parameter were reduced for the samples 5%-8GS, 10%-4P4GS, and 10%-8PS. The lowest MARHE (reduction by 14%) and FIGRA (reduction by 46%) were determined for the materials labeled as 10%-8GS. The total heat release (THR) values were similar, except for sample 5%-8PS, and ranged from 135 to 143 MJ m^−2^. The THR was dependent to a certain extent on the flame burning time, and its lowest value was obtained for the samples with the shortest time to flameout (TTF), i.e., for 1%-8GS, 1%-8PS, and 10%-8PS.

**Figure 8:**
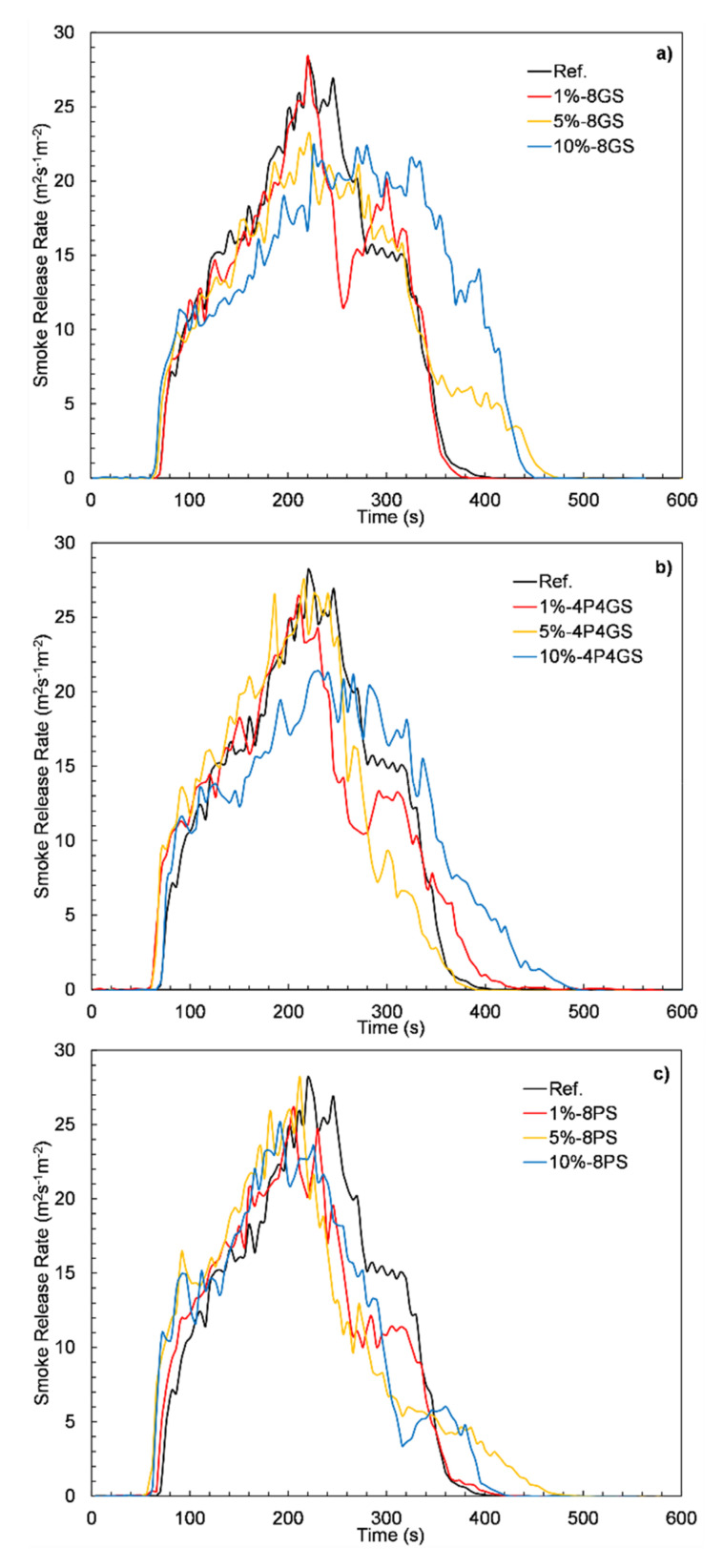
Smoke release rate (SRR) curves of the epoxy resin samples modified with (**a**) 8GS, (**b**) 4P4GS, and (**c**) 8PS silsesquioxane.

The addition of functionalized silsesquioxanes also had a positive effect on the amount of smoke emitted, which was evaluated based on the specific extinction area (SEA) and the total smoke release (TSR) values, as well as the smoke release rate (SRR) curves presented in Figure 8. As shown in Table 10, the SEA was reduced as a consequence of the addition of the investigated substances. In contrast to the pHRR, the highest values of the SEA were obtained for the sample 10%-8GS, while the lowest values were found for the samples 1%-8GS or 1%-8PS and 5%-4P4GS. A similar tendency was observed for the TSR, whose highest reduction (by approximately 9%) was achieved for 1%-8PS.

Analysis of the results allowed us to conclude that while the lowest heat release rate was measured for the epoxy resin with the reactive flame retardant, a greater reduction in smoke emission was noted for the samples modified with an additive flame retardant containing phosphorus groups.

It is to be noted that only small amounts of silsesquioxanes were used to prepare the polymers with reduced flammability and smoke emission; however, it seems reasonable to combine them with intumescent flame retardants to get the enhanced synergy of the individual components, leading to a complete flame retardancy of polymeric materials.

## 4. Conclusions

This study aimed to verify the effect of the structure of phosphate cage-like silsesquioxane derivatives and their ability to chemically bond to the polymer matrix on the surface and their mechanical and thermal properties, as well as the flammability of epoxy resins modified with their use. The obtained results show that the chemical structure of the obtained derivatives and their ability to chemically bond to the polymer matrix (epoxy resin) did not significantly affect the elasticity modulus or hardness of the modified materials or their surface properties (hydrophobicity). However, a meaningful impact of the chemical structure of the investigated derivatives on the thermal properties of the resin was observed. The epoxy derivatives that were capable of interacting with the polymer matrix through the formation of covalent bonds were evidenced to cause a notable reduction in the thermal decomposition rate of the obtained resins and the shift of the decomposition process toward higher temperatures. The derivatives that were chemically inert toward the modified materials containing only phosphate groups also reduced the rate of decomposition, but its onset was observed at lower temperatures than for pure epoxy resin.

In contrast, the derivatives containing phosphate and epoxy groups that were capable of chemical reactions with the modified resin were responsible for the lowering of the onset temperature of thermal decomposition, a decrease in the rate of decomposition, and its spread over time.

The differences in the structures of the tested silsesquioxane derivatives also had a clear impact on the flammability of epoxy resins modified with their use. Depending on the structure of the modifier used, reductions in the rate and amount of heat released, the rate of flame spread, or the amount of smoke emitted were observed. However, none of the tested derivatives displayed improvement of all measured parameters at once.

It should also be noted that the measured thermal parameters should be assessed through the prism of the content of additives used, whose amount was calculated in terms of the content of silicon and phosphorus atoms, did not exceed 1.7 and 0.8% by weight, respectively, as estimated by the EDS analysis.

This led to the conclusion that silsesquioxane derivatives can be considered as effective modifiers of thermal properties of epoxy resins; however, the choice of their chemical structure must be dictated by the assumed target parameters of the designed material.

## Figures and Tables

**Table 1:** Epoxy resin samples’ compositions.

Sample	E 652 (g)	IDA (g)	Modifier	Modifier Mass (g)
Ref.	40	20	-	-
1%-8GS		20.166	8GS	0.4
5%-8GS		20.828	8GS	2
10%-8GS		21.656	8GS	4
1%-4P4GS		20.056	4P4GS	0.4
5%-4P4GS		20.28	4P4GS	2
10%-4P4GS		20.56	4P4GS	4
1%-8PS		20	8PS	0.4
5%-8PS		20	8PS	2
10%-8PS		20	8PS	4

E 652—Epidian 652 epoxy resin; IDA—isophoronediamine; Ref.—unmodified epoxy resin as a reference sample; 1, 5 and 10%-8GS—epoxy resin samples containing 1, 5 or 10% of 8GS silsesquioxane derivative; 1, 5 and 10%-4P4GS—epoxy resin samples containing 1, 5 or 10% of 4P4GS silsesquioxane derivative; 1, 5 and 10%-8PS—epoxy resin samples containing 1, 5 or 10% of 8PS silsesquioxane derivative.

**Table 2:** Calculated and measured weights of Si and P in the prepared samples.

Sample	Calculated Element Weight (%)		Measured Element Weight (%)		Error (%)	
	Si	P	Si	P	Si	P
Ref	-	-	-	-	-	-
1%-8GS	0.15	-	0.19	0.01	7.05	55.63
5%-8GS	0.74	-	0.91	0.01	3.59	56.14
10%-8GS	1.42	-	1.69	0.01	3.13	55.94
1%-4P4GS	0.1	0.03	0.07	0.02	18.71	54.88
5%-4P4GS	0.51	0.14	0.4	0.1	4.86	12.84
10%-4P4GS	0.97	0.27	0.88	0.25	3.48	6.61
1%-8PS	0.08	0.04	0.12	0.07	11.79	19.81
5%-8PS	0.38	0.21	0.3	0.17	5.21	8.55
10%-8PS	0.74	0.41	0.73	0.41	3.68	4.99

**Table 3:** Water contact angles (WCAs) of the modified epoxy resins.

Sample	WCA (°)
Ref	77.1 ± 3.4
1%-8GS	81 ± 4.69
5%-8GS	81.7 ± 1.93
10%-8GS	80.2 ± 3.9
1%-4P4GS	82.7 ± 2.55
5%-4P4GS	81 ± 0.97
10%-4P4GS	81.3 ± 1.75
1%-8PS	82.5 ± 0.8
5%-8PS	79.7 ± 1.04
10%-8PS	50.1 ± 3.31

**Table 4:** Mechanical properties of modified epoxy resins.

Sample	Modulus (500–1500 nm)		Hardness (500–1500 nm)	
	Mean (MPa)	Std. Dev. (%)	Mean (MPa)	Std. Dev. (%)
Ref.	3064	1.6	127	1.7
1%-8GS	3555	1.6	163	1.7
5%-8GS	3149	1.4	136	1.7
10%-8GS	3149	0.9	137	1.2
1%-4P4GS	3071	1.2	129	1.4
5%-4P4GS	3112	0.8	130	2.8
10%-4P4GS	3086	1	132	1.1
1%-8PS	3127	0.6	137	1.1
5%-8PS	3210	1.8	139	2.1
10%-8PS	3230	0.7	145	0.9

Std. Dev.—standard deviation.

**Table 5:** Results of the TG analysis for epoxy resins modified with 1, 5, and 10 wt.% of silsesquioxane derivatives in the N_2_ atmosphere.

Sample	Mass Loss Temperature (°C)							Char Yield @ 800 °C (%)
	T_1%_	T_5%_	T_10%_	T_20%_	T_40%_	T_60%_	T_80%_	
Ref.	104.2	155.4	201.3	325.7	350.8	368.8	401.6	6.6
1%-8GS	96.7	146.8	190.3	325.4	352	369.7	404.4	7.6
5%-8GS	100.1	143.8	189	329.1	356.3	377.7	424.9	10.9
10%-8GS	111	153.3	199.6	320	355.1	386.6	469.2	14.1
1%-4P4GS	104	151.7	196	326.3	351.7	368.8	401.5	6.9
5%-4P4GS	105.6	152.4	198.2	318.5	344.6	364	400	8
10%-4P4GS	101.3	149.3	194.9	315.1	342.2	363.4	412.1	10.2
1%-8PS	110.7	161.4	208.9	321.8	347.6	366.9	400.2	7.2
5%-8PS	104.1	152.3	197.6	317.8	343.2	361.9	392.6	7.8
10%-8PS	106.6	154.9	202.9	309.2	334.7	353.9	392.2	8.7

T_1%,_ T_5%,_ T_10%,_ T_20%,_ T_40%,_ T_60%,_ T_80%_—temperatures of 1, 5, 10, 20, 40, 60 and 80% mass loss, respectively.

**Table 6:** Results of the DTG analysis for epoxy resins modified with 1, 5, and 10 wt.% of silsesquioxane derivatives in the N_2_ atmosphere.

Sample	Step I				Step II			
	Range (°C)	Change (%)	T_max_ (°C)	Rate (%K^−1^)	Range (°C)	Change (%)	T_max_ (°C)	Rate (%K^−1^)
Ref.	41–270	14.1	176	0.11	270–600	78.7	353.8	1.14
1%-8GS	52–260	13.6	161.3	0.12	260–600	77.7	362.8	1.15
5%-8GS	44–255	12.7	150.5	0.12	255–600	75.9	361.8	0.99
10%-8GS	40–255	13.8	161.3	0.12	255–600	71.1	345.7	0.72
1%-4P4GS	46–263	13.5	167.8	0.12	263–600	79	356.7	1.26
5%-4P4GS	41–260	13.9	167.7	0.11	260–600	77.5	354.5	1
10%-4P4GS	40–250	12.8	161.3	0.12	250–650	76.6	349.8	1
1%-8PS	50–270	14.2	180.5	0.11	270–600	78.1	359.8	1.1
5%-8PS	45–255	13.2	167.2	0.12	255–600	78.4	350.8	1.1
10%-8PS	−250	13.7	170.2	0.11	250–600	77.3	346.2	1.1

T_max_—temperature of maximum decomposition rate.

**Table 7:** Results of the TG analysis for epoxy resins modified with 1, 5, and 10 wt.% of silsesquioxane derivatives in the air atmosphere.

Sample	Mass Loss Temperature (°C)							Residue @ 800 °C (%)
	T_1%_	T_5%_	T_10%_	T_20%_	T_40%_	T_60%_	T_80%_	
Ref	113.5	171.3	222.8	318.7	343.3	363.5	490	0.96
1%-8GS	104.7	153.1	197.8	312.8	346.4	374.4	457	0.44
5%-8GS	94.7	143.2	184.3	305.8	350.7	387.1	543.9	0.59
10%-8GS	104.3	148.6	191.6	309.8	355.8	400	561.9	2
1%-4P4GS	106.1	157	202.4	312.3	345	369.7	431.3	0
5%-4P4GS	105.2	154.4	198.3	308.1	340.4	364.4	497.8	0.8
10%-4P4GS	98.8	147.5	190.3	301.3	339.6	368.7	542	1.48
1%-8PS	109.4	158	202.7	312.3	343.9	371	436.2	0.23
5%-8PS	99	147.4	190.2	300.7	337.8	359.7	501.2	0
10%-8PS	102.2	149.9	197.2	297.6	333.5	361.3	537.7	2.73

**Table 8:** Results of the DTG analysis for epoxy resins modified with 1, 5, and 10 wt.% of silsesquioxane derivatives in the air atmosphere.

Sample	Step I				Step II				Step III			
	Range (°C)	Change (%)	T_max_ (°C)	Rate (%K^−1^)	Range (°C)	Change (%)	T_max_ (°C)	Rate (%K^−1^)	Range (°C)	Change (%)	T_max_ (°C)	Rate (%K^−1^)
Ref.	55–260	13.4	196.8	0.1	260–470	65.6	333.5	1.14	470–680	20.1	569.5	0.2
1%-8GS	45–257	14	169.3	0.12	257–503	68.6	341.5	0.9	503–738	17	587.5	0.14
5%-8GS	30–246	14.1	158	0.13	246–489	61.7	350.5	0.67	489–733	23.5	593.8	0.18
10%-8GS	30–247	13.7	159.3	0.12	247–498	61	343	0.66	498–746	23.3	606.3	0.17
1%-4P4GS	42–257	14	177	0.11	257–514	72.4	348.2	0.97	514–714	13.6	600.2	0.13
5%-4P4GS	41–251	14	172.8	0.12	251–483	65.3	336.8	1	483–734	19.9	580.3	0.16
10%-4P4GS	47–240	13.6	162	0.12	240–483	62.9	335.7	0.8	483–754	21.9	604.5	0.16
1%-8PS	30–255	13.9	175.3	0.12	255–413	71.4	335.3	0.9	513–733	14.5	612.3	0.13
5%-8PS	52–243	13.9	164.7	0.12	243–479	65.1	344.7	0.96	479–743	21	589.7	0.16
10%-8PS	57–233	12.6	160.2	0.11	233–480	64	335.3	0.92	480–736	20.4	591.7	0.15

**Table 9:** Results of the DSC analysis showing the glass temperature (T_g_) for epoxy resins modified with 1 and 10% of the silsesquioxane derivatives.

Sample	T_g_ (°C)
Ref.	43.93
1%-8GS	44.44
10%-8GS	42.24
1%-4P4GS	45.91
10%-4P4GS	41.47
1%-8PS	43.57
10%-8PS	39.88

**Table 10:** Cone calorimeter results of epoxy resins with different amounts of silsesquioxanes.

Sample	TTI (s)	TTF (s)	pHRR (kWm^−2^)	MARHE (kWm^−2^)	FIGRA (kWm^−2^s^−1^)	THR (MJm^−2^)	SEA (m^2^kg^−1^)	TSR (m^2^m^−2^)
Ref.	63	393	854.5	405.9	3.9	139.4	842.2	4733.4
1%-8GS	65	380	932	399.2	4.1	138.2	796.7	4435
5%-8GS	56	485	678.2	372.5	2.9	143.1	832.4	4844
10%-8GS	58	455	566	348.8	2.1	144.6	932.7	5538.4
1%-4P4GS	51	470	861.3	412.7	4.1	141.1	822.1	4527.6
5%-4P4GS	60	402	845.3	436.7	4.3	143.7	755.1	4480.1
10%-4P4GS	67	528	658.5	349.8	2.8	143.3	833.4	4970.4
1%-8PS	60	425	931.1	413.1	4.5	137.9	790.8	4309.9
5%-8PS	57	526	869.2	430.8	4.3	153.2	830.4	4574.1
10%-8PS	60	438	776.6	399.7	3.8	134.9	835.6	4529.4

TTI: time to ignition, TTF: time to flameout, pHRR: peak heat release rate, MARHE: maximum average rate of the heat emission, FIGRA: fire growth rate, THR: total heat release, SEA: specific extinction area, TSR: total smoke release.

## Supplementary Materials

The NMR and FT-IR spectra of the isolated compounds and the results of the SEM-EDS, DSC, and TG analyses are available online at https://www.mdpi.com/1996-1944/13/23/5373/s1. Figure S1: Structures of DOPA and DOPO (from left, respectively). Figure S2: FT-IR spectra of DOPA and DOPO. Figure S3: 1H NMR spectrum of DOPA. Figure S4: 13C NMR spectrum of DOPA. Figure S5: 31P NMR spectrum of DOPA. Figure S6: 1H NMR spectrum of 8GS derivative. Figure S7: 13C NMR spectrum of 8GS derivative. Figure S8: 29Si NMR spectrum of 8GS compound. Figure S9: 1H NMR spectrum of 4P4GS derivative. Figure S10: 13C NMR spectrum of 4P4GS derivative. Figure S11: ^31^P NMR spectrum of 4P4GS derivative. Figure S12: ^29^Si NMR spectrum of 4P4GS derivative. Figure S13: ^1^H NMR spectrum of 8PS derivative. Figure S14: ^13^C NMR spectrum of 8PS derivative. Figure S15: ^31^P NMR spectrum of 8PS derivative. Figure S16: ^29^Si NMR spectrum of 8PS derivative. Figure S17. EDS spectrum of unmodified epoxy resin (Ref.) sample. Figure S18: SEM micrograph of unmodified epoxy resin (Ref.) sample. Figure S19: SEM-EDS (**a**) carbon, (**b**) oxygen atoms distribution maps of the unmodified epoxy resin Ref. sample. Figure S20: EDS spectrum of 1%-8GS sample. Figure S21: SEM micrograph of 1%-8GS sample. Figure S22: SEM-EDS (**a**) carbon, (**b**) oxygen, and (**c**) silicon atoms distribution maps of 1%-8GS sample. Figure S23: EDS spectrum of 5%-8GS sample. Figure S24: SEM micrograph of 5%-8GS sample. Figure S25: SEM-EDS (**a**) carbon, (**b**) oxygen, and (**c**) silicon atoms distribution maps of 5%-8GS sample. Figure S26: EDS spectrum of 10%-8GS sample. Figure S27: SEM micrograph of 10%-8GS sample. Figure S28. SEM-EDS (**a**) carbon, (**b**) oxygen, and (**c**) silicon atoms distribution maps of 10%-8GS sample. Figure S29: EDS spectrum of 1%-4P4GS sample. Figure S30: SEM micrograph of 1%-4P4GS sample. Figure S31. SEM-EDS (**a**) carbon, (**b**) oxygen, (**c**) silicon and(**d**) phosphorus atoms distribution maps of 1%-4P4GS sample. Figure S32: EDS spectrum of 5%-4P4GS sample. Figure S33: SEM micrograph of 5%-4P4GS sample. Figure S34: SEM-EDS (**a**) carbon, (**b**) oxygen, (**c**) silicon and (**d**) phosphorus atoms distribution maps of 5%-4P4GS sample. Figure S35: EDS spectrum of 10%-4P4GS sample. Figure S36: SEM micrograph of 10%-4P4GS sample. Figure S37: SEM-EDS (**a**) carbon, (**b**) oxygen, (**c**) silicon and (**d**) phosphorus atoms distribution maps of 10%-4P4GS sample. Figure S38: EDS spectrum of 1%-8PS sample. Figure S39: SEM micrograph of 1%-8PS sample. Figure S40: SEM-EDS (**a**) carbon, (**b**) oxygen, (**c**) silicon and (**d**) phosphorus atoms distribution maps of 1%-PS sample. Figure S41: EDS spectrum of 5%-8PS sample. Figure S42: SEM micrograph of 5%-8PS sample. Figure S43. SEM-EDS (**a**) carbon, (**b**) oxygen, (**c**) silicon and (**d**) phosphorus atoms distribution maps of 5%-PS sample. Figure S44: EDS spectrum of 10%-8PS sample. Figure S45: SEM micrograph of 10%-8PS sample. Figure S46: SEM-EDS (**a**) carbon, (**b**) oxygen, (**c**) silicon and (**d**) phosphorus atoms distribution maps of 10%-PS sample. Figure S47: Graphs of (**a**) TG and (**b**) DTG curves of epoxy resin samples modified with 1, 5 and 10%wt. of 8GS derivative in N2 atmosphere. Figure S48: Graphs of (**a**) TG and (**b**) DTG curves of epoxy resin samples modified with 1, 5 and 10% wt. of 8GS derivative in air atmosphere. Figure S49: Graphs of **a**) TG and (**b**) DTG curves of epoxy resin samples modified with 1, 5 and 10% wt. of 4P4GS derivative in N2 atmosphere. Figure S50: Graphs of (**a**) TG and (**b**) DTG curves of epoxy resin samples modified with 1, 5 and 10% of 4P4GS derivative in air atmosphere. Figure S51: Graphs of (**a**) TG and (**b**) DTG curves of epoxy resin samples modified with 1, 5 and 10% wt. of 8PS derivative in N2 atmosphere. Figure S52: Graphs of (**a**) TG and (**b**) DTG curves of epoxy resin samples modified with 1, 5 and 10% wt. of 8PS derivative in air atmosphere. Figure S53: DSC heating curves for epoxy resins modified with 1%wt. of 8GS, 4P4GS and 8PS derivatives. Figure S54: DSC cooling curves for epoxy resins modified with 1%wt. of 8GS, 4P4GS and 8PS derivatives. Figure S55: DSC heating curves for epoxy resins modified with 10%wt. of 8GS, 4P4GS and 8PS derivatives. Figure S56: DSC cooling curves for epoxy resins modified with 10%wt. of 8GS, 4P4GS and 8PS derivatives. Table S1: Results of EDS analysis of unmodified epoxy resin (Ref.) sample. Table S2: Results of EDS analysis of 1%-8GS sample. Table S3: Results of EDS analysis of 5%-8GS sample. Table S4. Results of EDS analysis of 10%-8GS sample. Table S5: Results of EDS analysis of 1%-4P4GS sample. Table S6: Results of EDS analysis of 5%-4P4GS sample. Table S7: Results of EDS analysis of 10%-4P4GS sample. Table S8. Results of EDS analysis of 1%-PS sample. Table S9: Results of EDS analysis of 5%-PS sample. Table S10: Results of EDS analysis of 10%-PS sample.
