# Supplementary material for: Phosphorus-Containing Silsesquioxane Derivatives as Additive or Reactive Components of Epoxy Resins
Source: Materials (Basel). 2020 Nov 26;13(23):5373. doi: 10.3390/ma13235373 (PMC7730412; doi:10.3390/ma13235373)
Supplement: Supplementary file 1 [file materials-13-05373-s001.pdf]

Supplementary Materials

Phosphorus-Containing Silsesquioxane Derivatives as Additive or Reactive Components of Epoxy Resins

Mariusz Szołyga[1], Michał Dutkiewicz[1, 2], Marek Nowicki[1], Kamila Sałasińska[3], Maciej Celiński[3] and Bogdan Marciniec[1, 4 *]

[1] *Centre for Advanced Technologies, Adam Mickiewicz University in Poznan,*
Uniwersytetu Poznanskiego 10, 61-614 Poznan, Poland, e-mail: bogdan.marciniec@amu.edu.pl

[2] *Poznan Science and Technology Park, Adam Mickiewicz University Foundation,*
Rubiez 46, 61-612 Poznan, Poland

[3] *Department of Chemical, Biological and Aerosol Hazards, Central Institute for Labour Protection - National Research Institute, Czerniakowska 16, 00-701 Warsaw, Poland*

[4] *Faculty of Chemistry, Adam Mickiewicz University in Poznan, Uniwersytetu Poznanskiego 8, 61-614 Poznan, Poland,*

Table of Content

DOPA spectroscopic analysis

8GS spectroscopic analysis

4P4GS spectroscopic analysis

8PS spectroscopic analysis

Results of DSC analysis of modified resins

Results of TG analysis of modified resins

Results of SEM-EDS analysis of modified resins

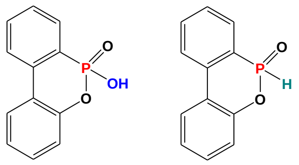

Figure S1. Structures of DOPA and DOPO (from left, respectively).

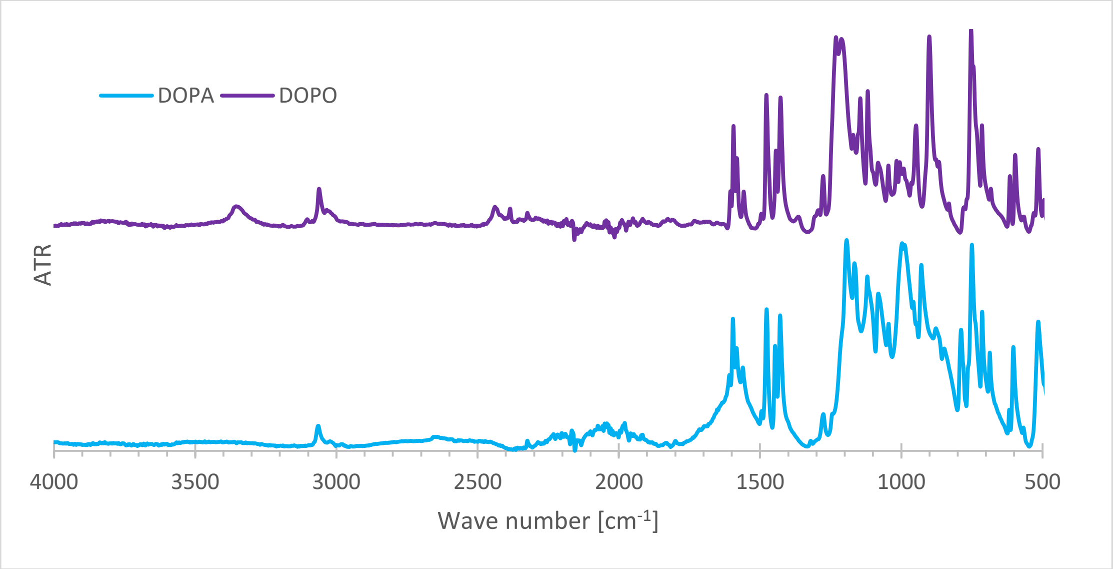

Figure S2. FT-IR spectra of DOPA and DOPO.

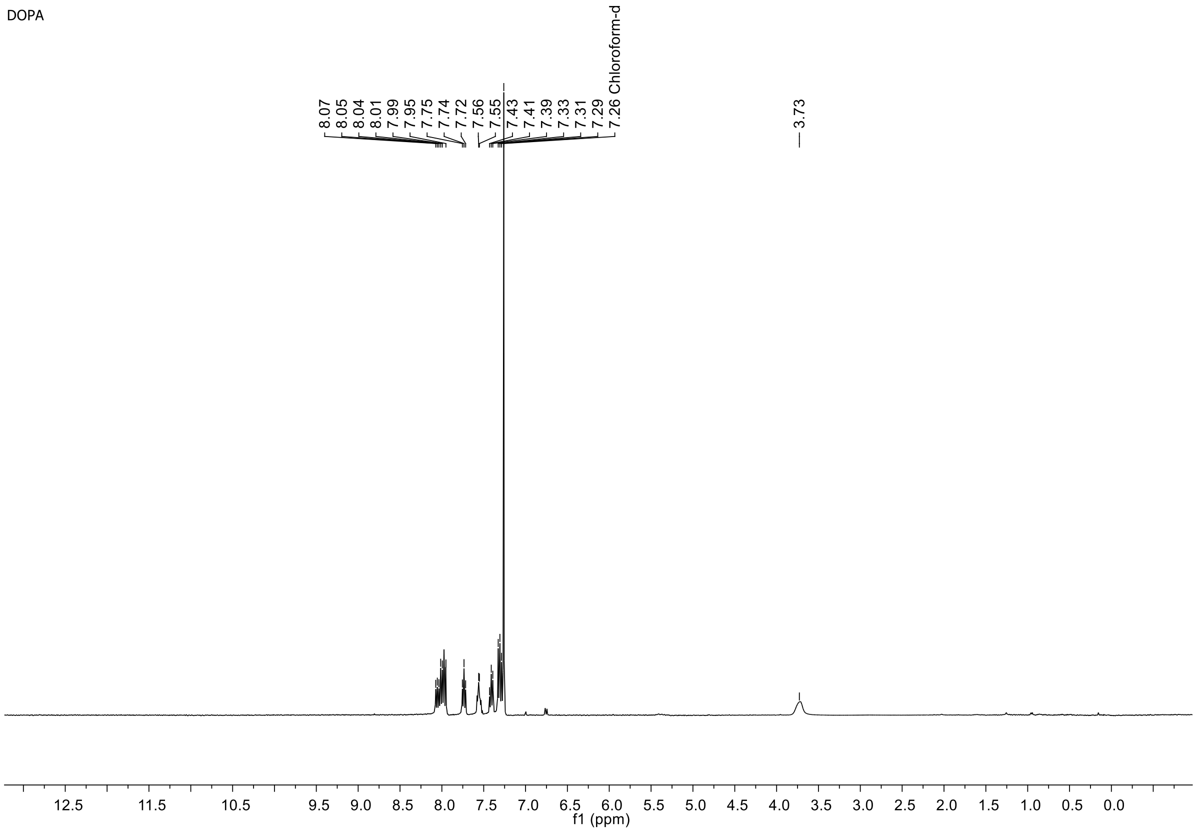

Figure S3. ^{1}H NMR spectrum of DOPA.

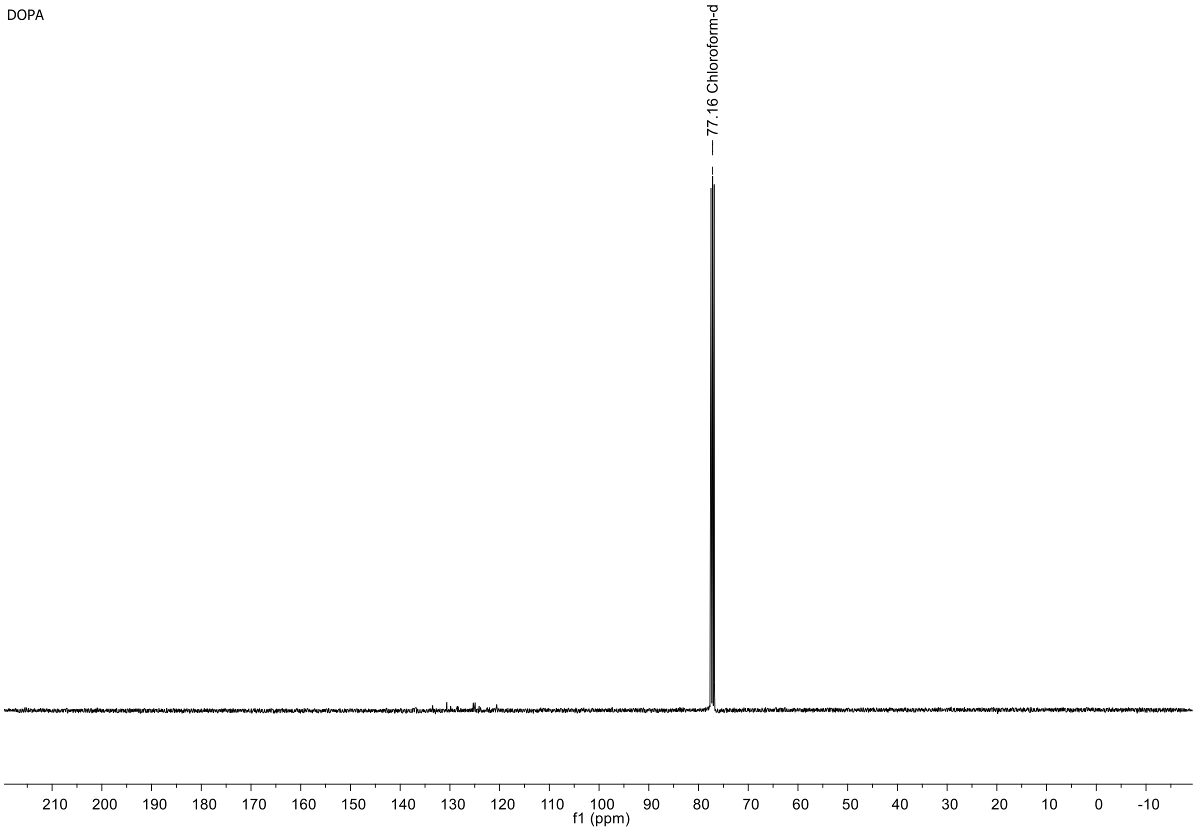

Figure S4. ^{13}C NMR spectrum of DOPA.

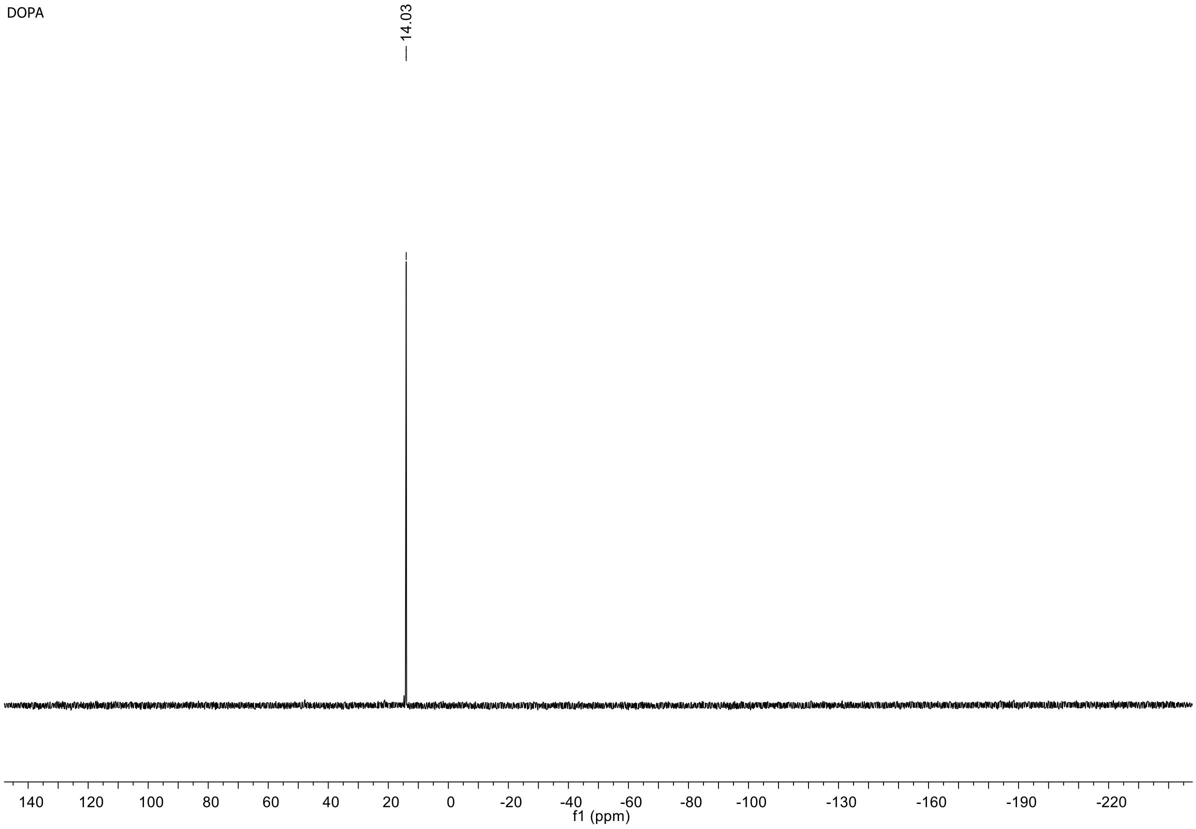

Figure S5. ^{31}P NMR spectrum of DOPA.

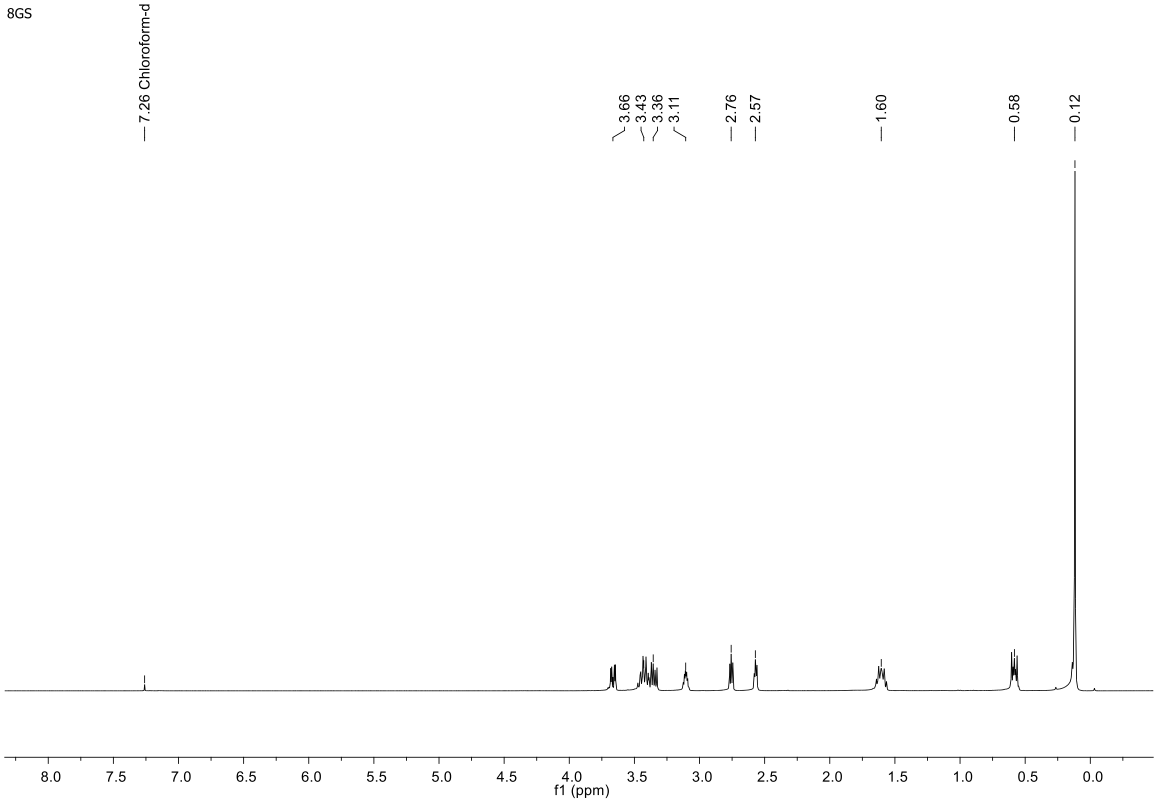

Figure S6. ^{1}H NMR spectrum of 8GS derivative.

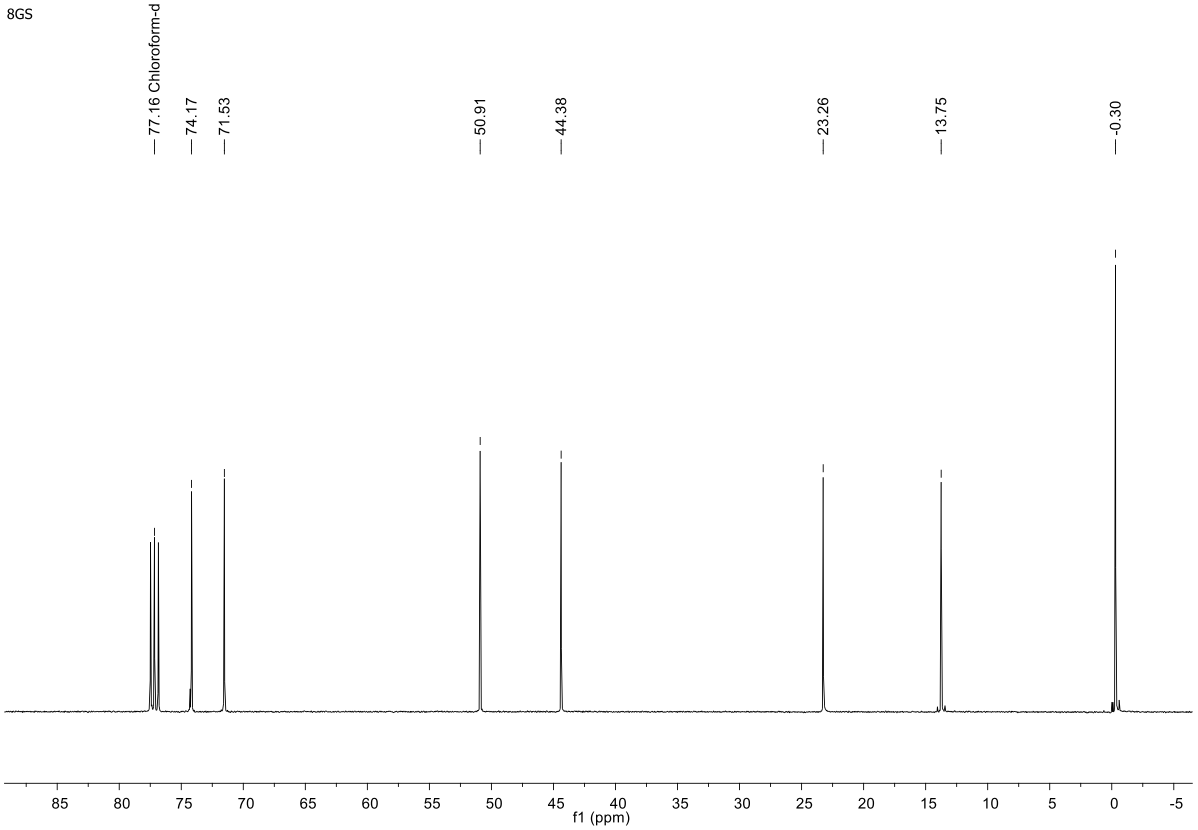

Figure S7. ^{13}C NMR spectrum of 8GS derivative.

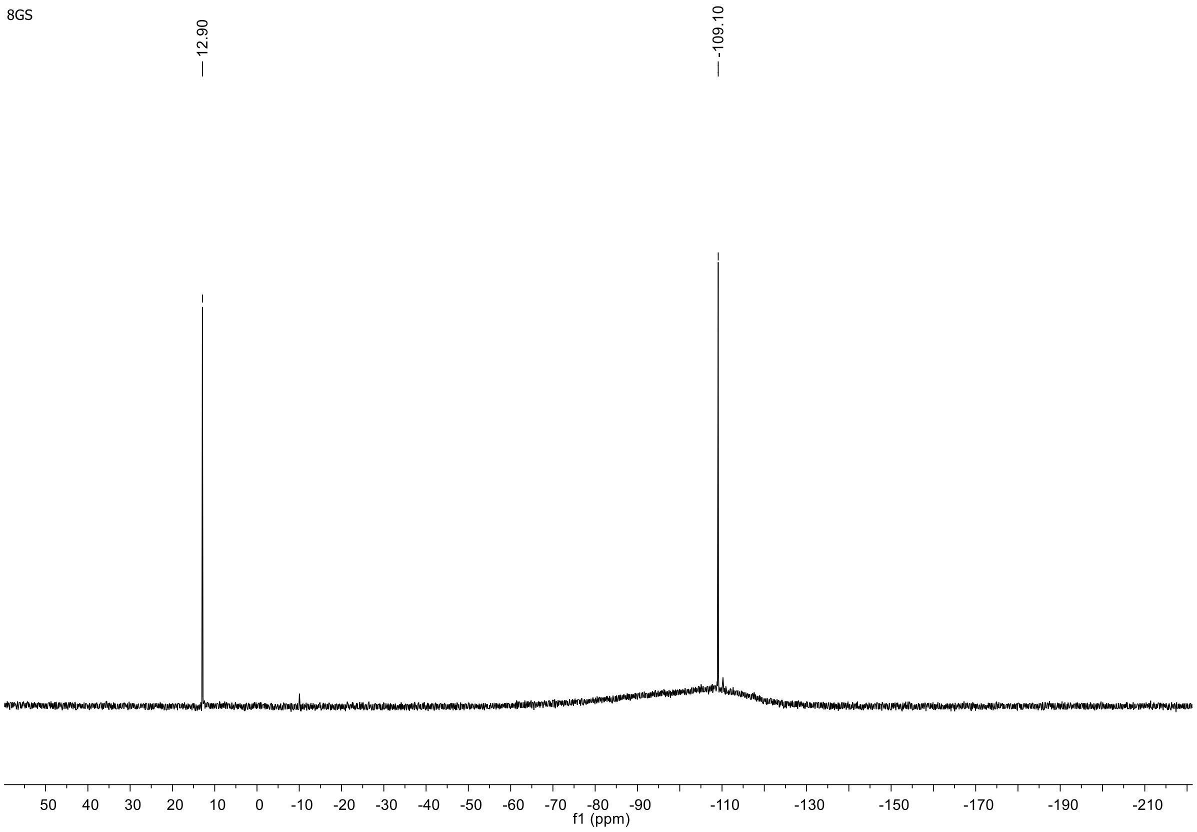

Figure S8. ^{29}Si NMR spectrum of 8GS compound.

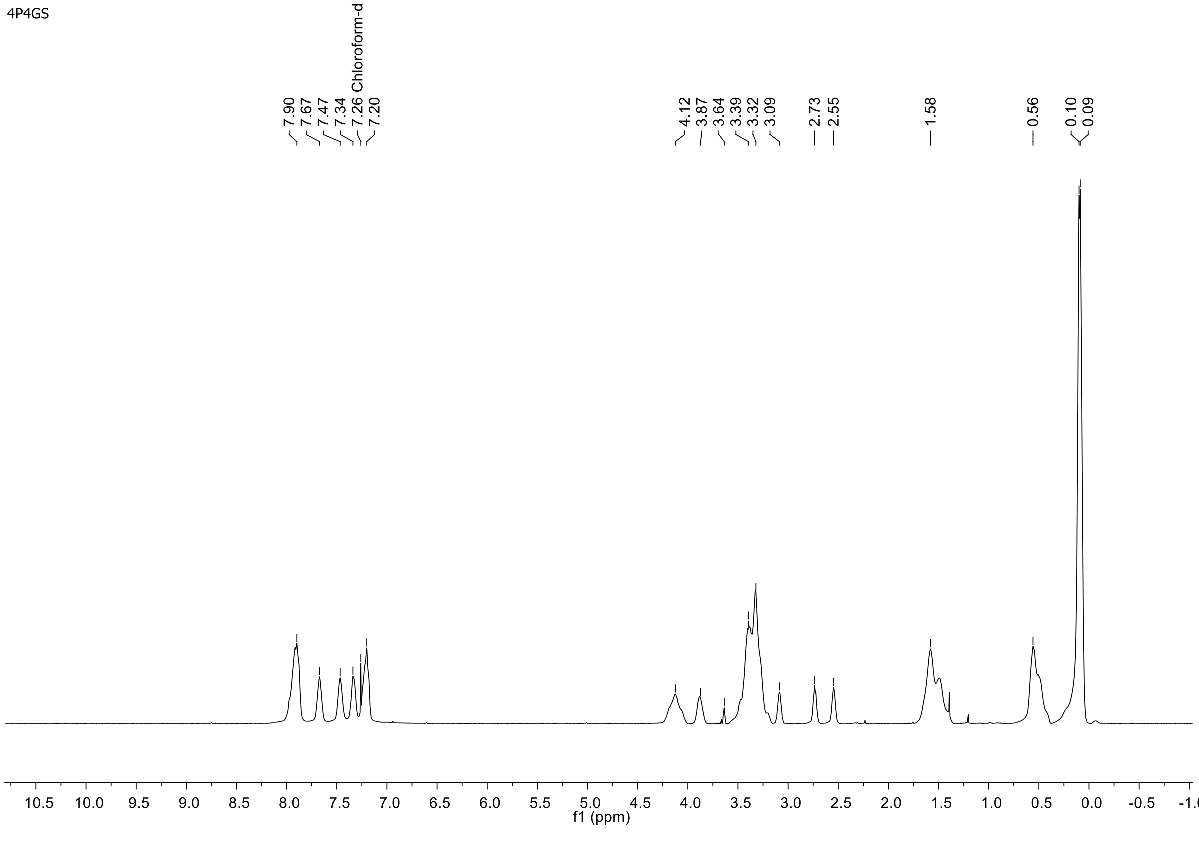

Figure S9. ^{1}H NMR spectrum of 4P4GS derivative.

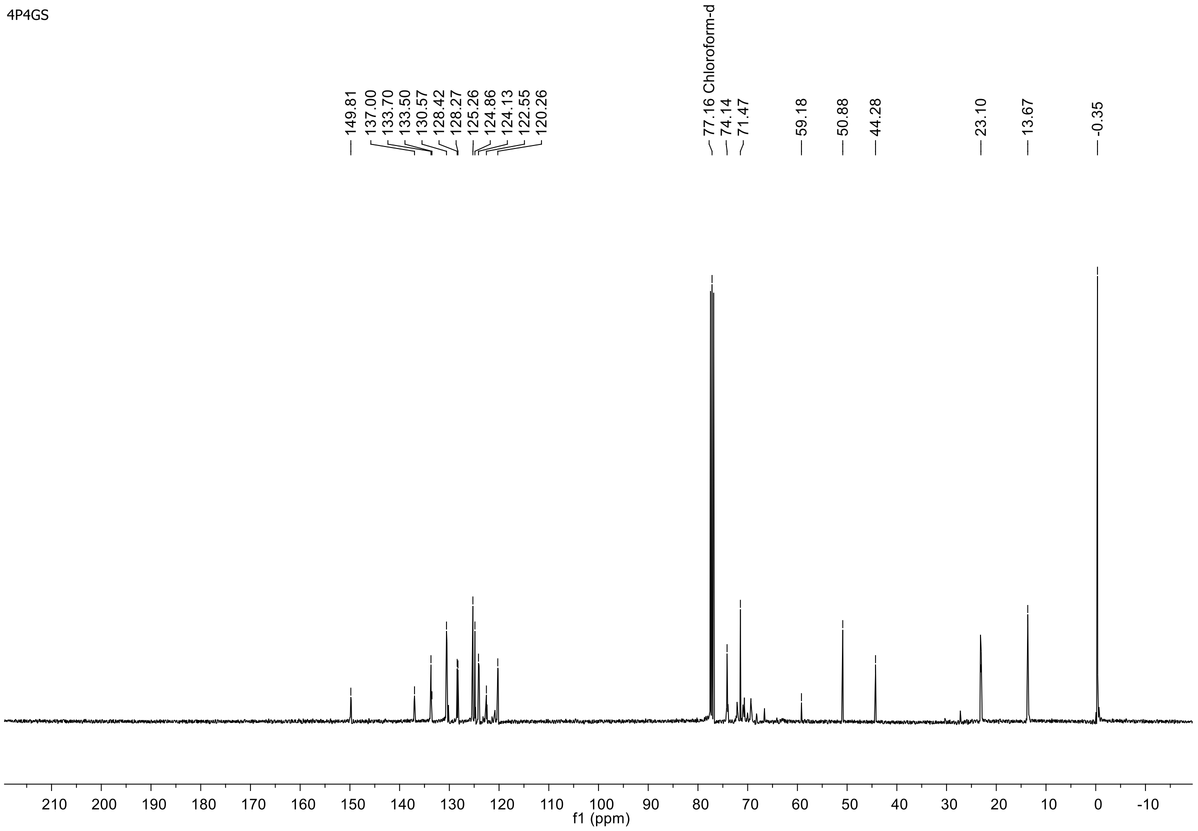

Figure S10. ^{13}C NMR spectrum of 4P4GS derivative.

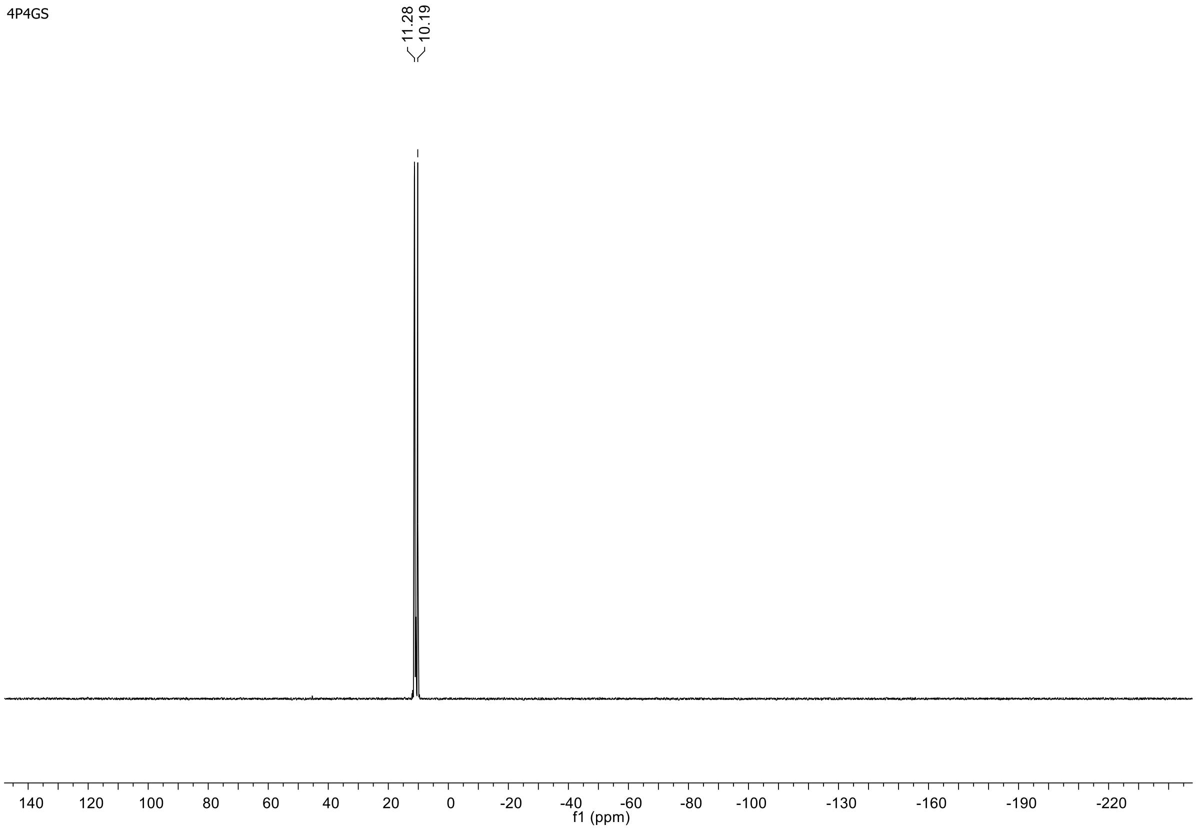

Figure S11. ^{31}P NMR spectrum of 4P4GS derivative.

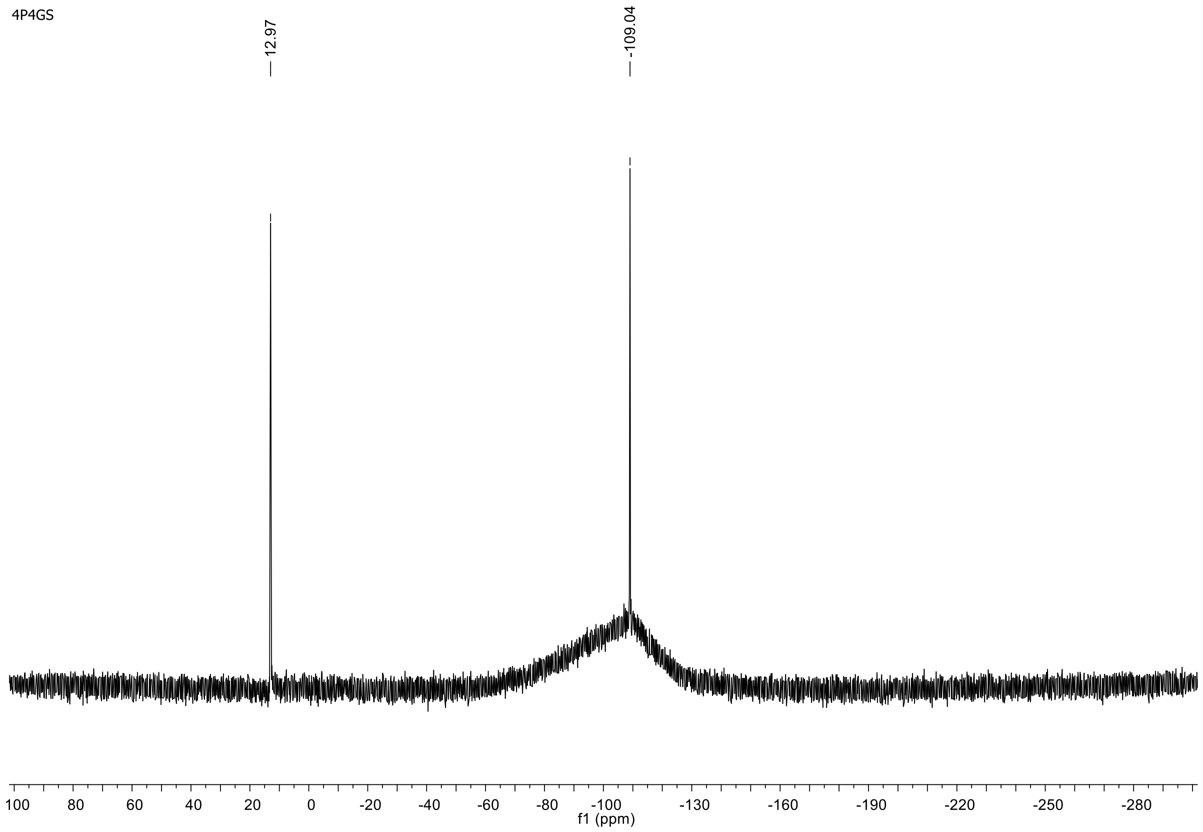

Figure S12. ^{29}Si NMR spectrum of 4P4GS derivative.

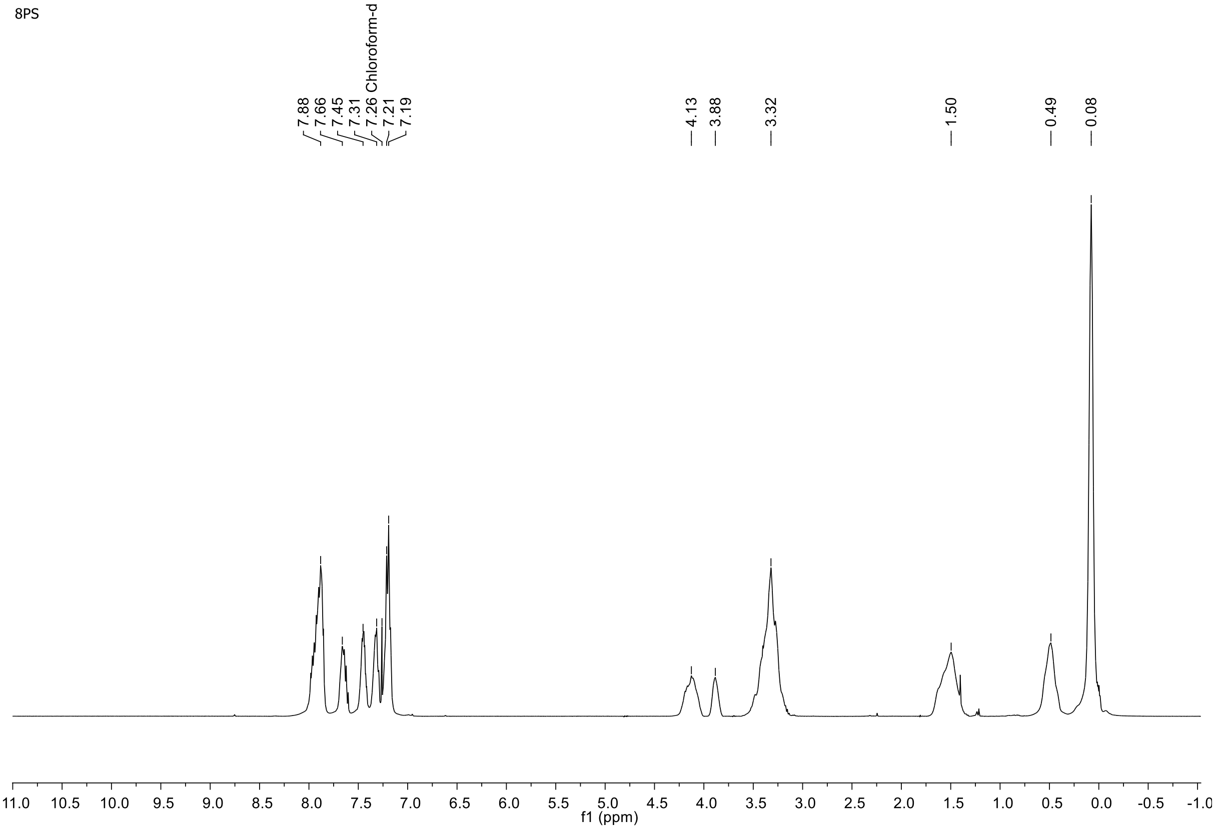

Figure S13. ^{1}H NMR spectrum of 8PS derivative.

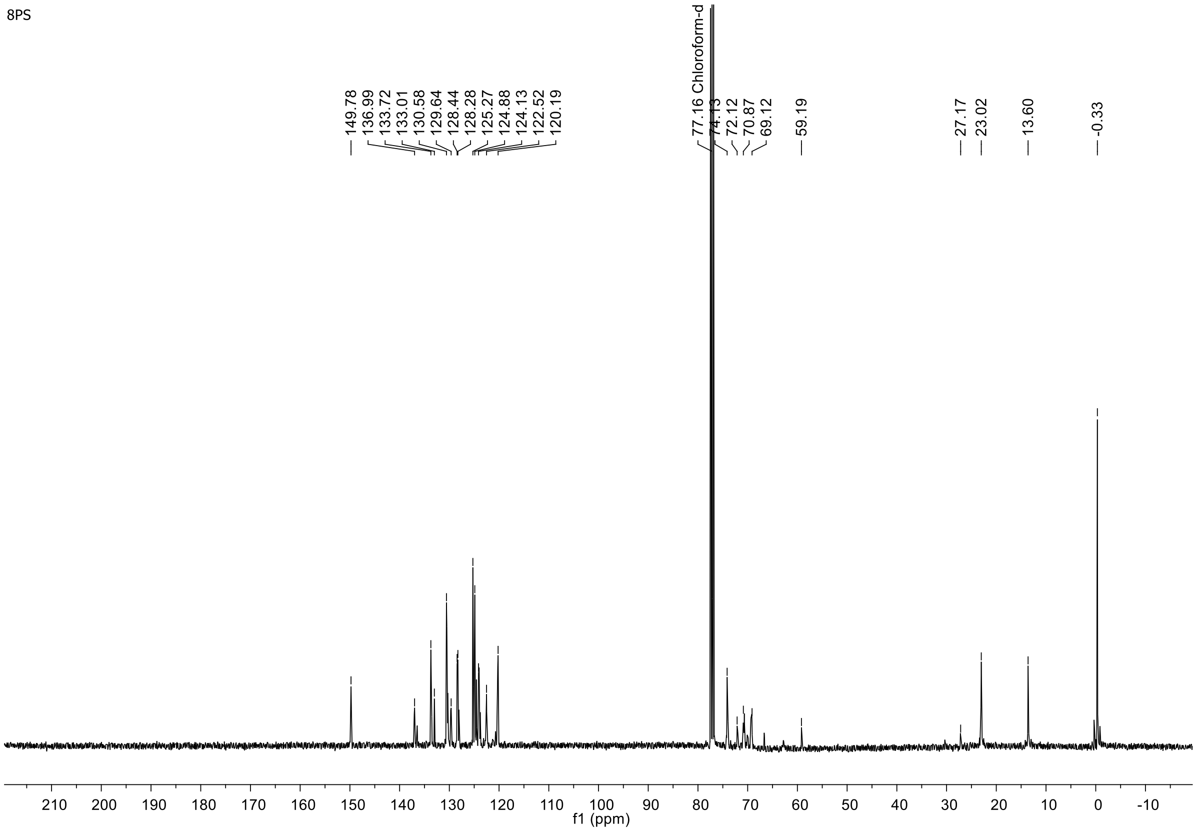

Figure S14. ^{13}C NMR spectrum of 8PS derivative.

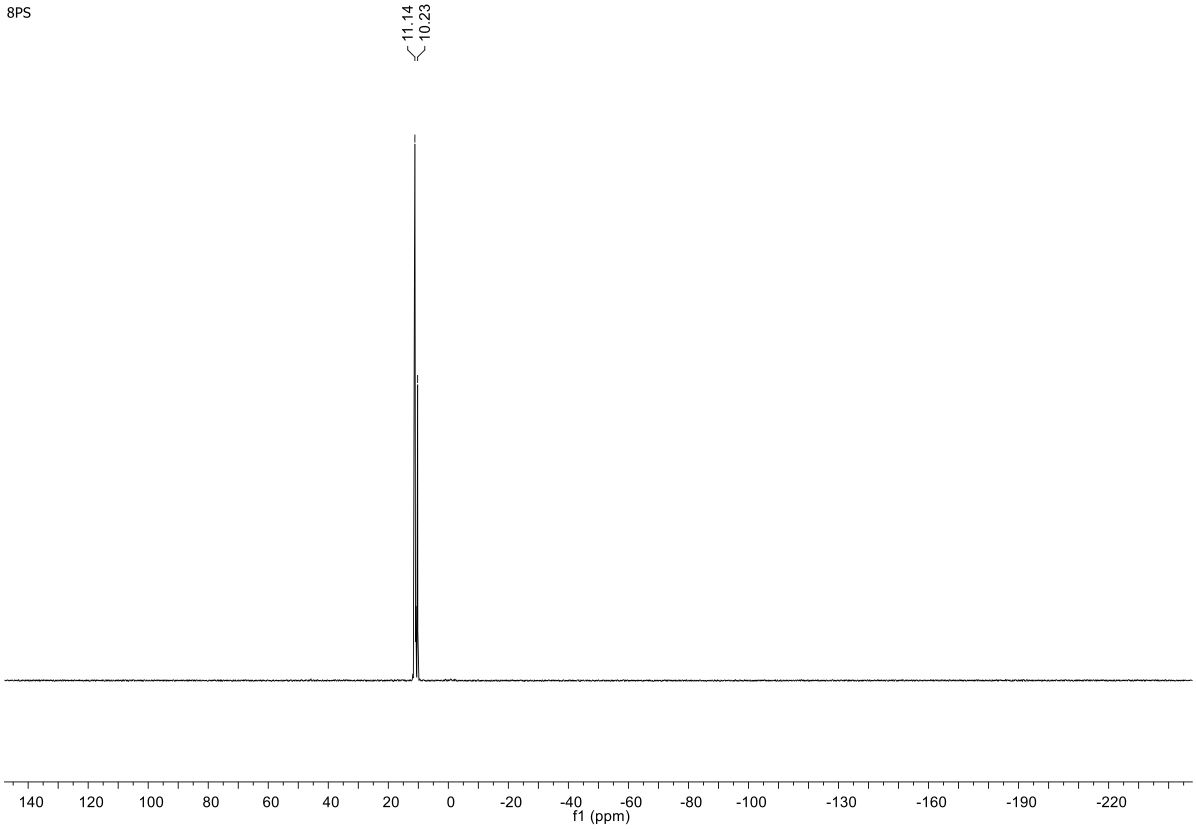

Figure S15. ^{31}P NMR spectrum of 8PS derivative.

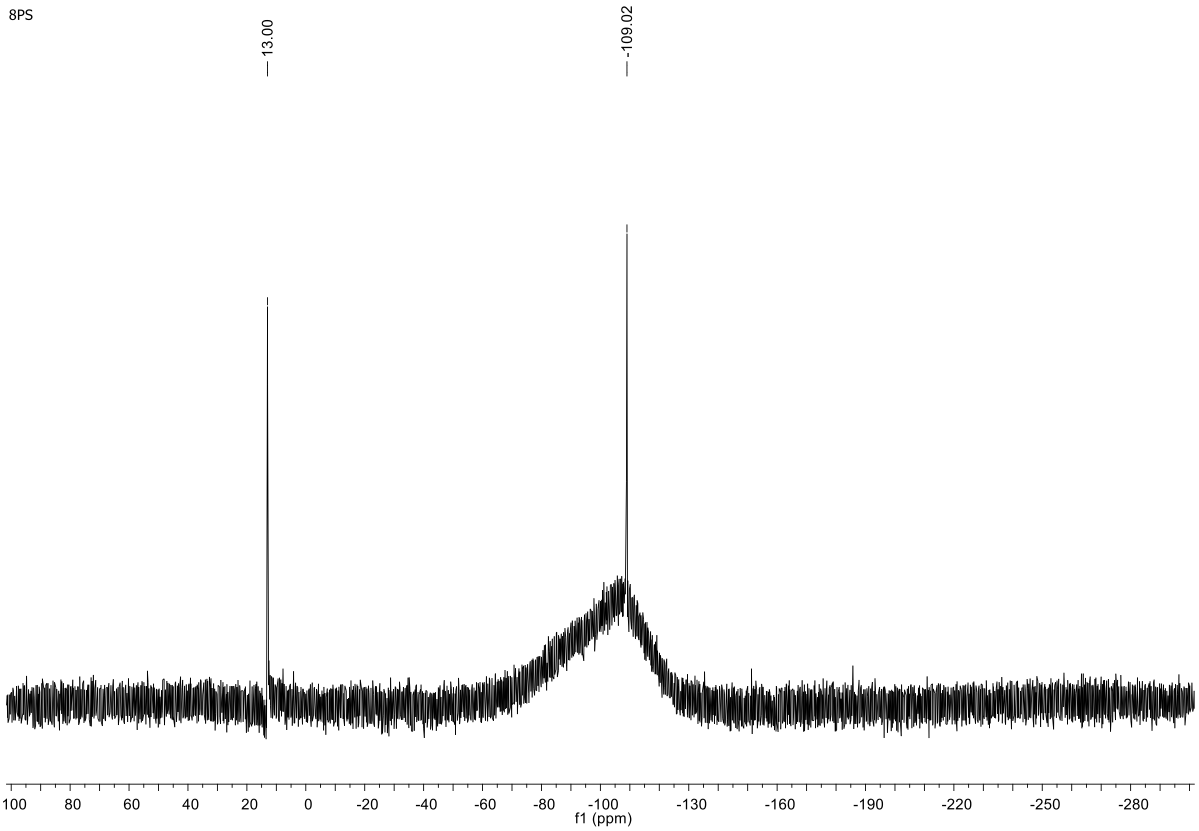

Figure S16. ^{29}Si NMR spectrum of 8PS derivative.

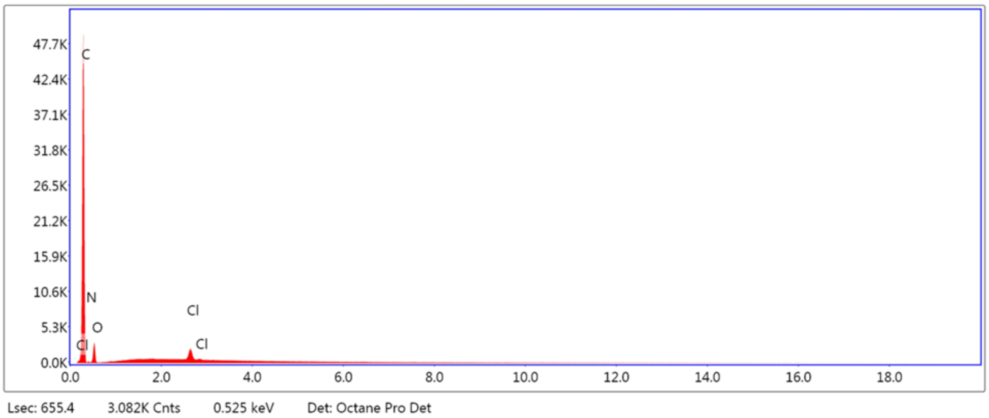

Figure S17. EDS spectrum of unmodified epoxy resin (Ref.) sample.

Table S1. Results of EDS analysis of unmodified epoxy resin (Ref.) sample

Element	Weight %	Atomic %	Net Int.	Error %	Kratio	Z	R	A	F
C K	83.02	86.72	710.19	3.43	0.6114	1.0064	0.9963	0.7318	1.0000
N K	1.94	1.74	1.65	16.12	0.0012	0.9860	1.0062	0.0618	1.0000
O K	14.44	11.33	45.10	10.36	0.0128	0.9682	1.0151	0.0918	1.0000
ClK	0.60	0.21	45.85	3.24	0.0054	0.8323	1.0736	1.0496	1.0257

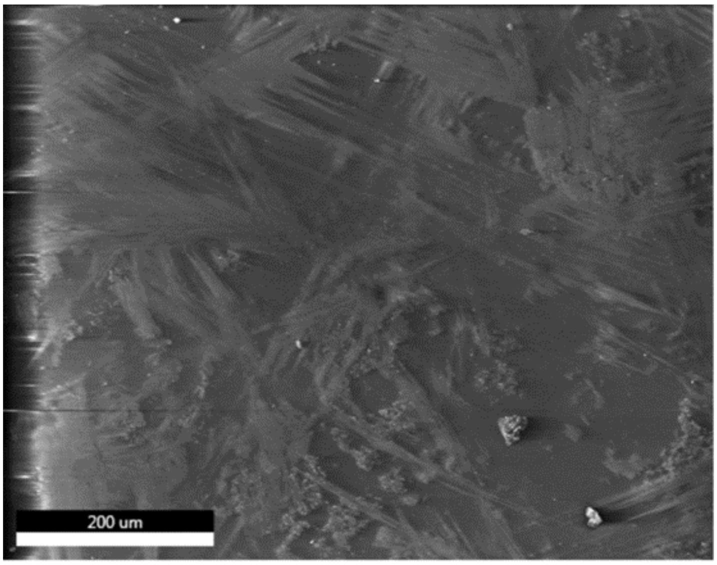

Figure S18. SEM micrograph of unmodified epoxy resin (Ref.) sample.

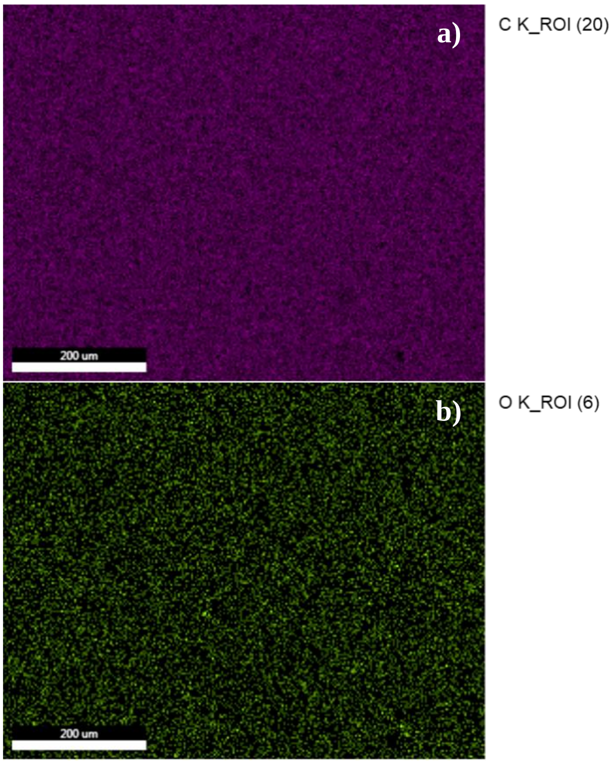

Figure S19. SEM-EDS a) carbon, b) oxygen atoms distribution maps of the unmodified epoxy resin Ref. sample.

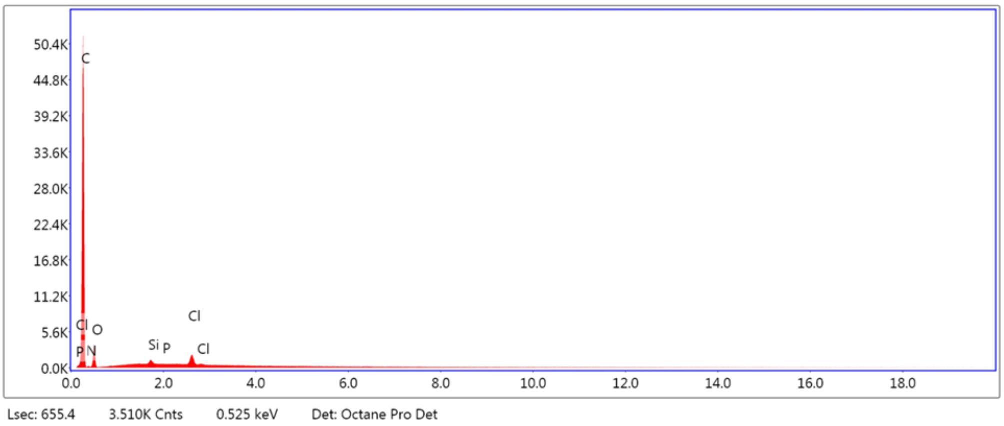

Figure S20. EDS spectrum of 1%-8GS sample.

Table S2. Results of EDS analysis of 1%-8GS sample.

Element	Weight %	Atomic %	Net Int.	Error %	Kratio	Z	R	A	F
C K	81.89	85.81	740.71	3.57	0.5919	1.0068	0.9960	0.7178	1.0000
N K	2.14	1.92	1.98	15.59	0.0013	0.9864	1.0059	0.0626	1.0000
O K	15.24	11.99	51.75	10.28	0.0137	0.9686	1.0149	0.0926	1.0000
SiK	0.19	0.08	14.65	7.05	0.0014	0.8902	1.0571	0.8117	1.0087
P K	0.01	0.00	0.80	55.63	0.0001	0.8562	1.0628	0.9242	1.0138
ClK	0.54	0.19	43.87	3.13	0.0048	0.8327	1.0734	1.0449	1.0254

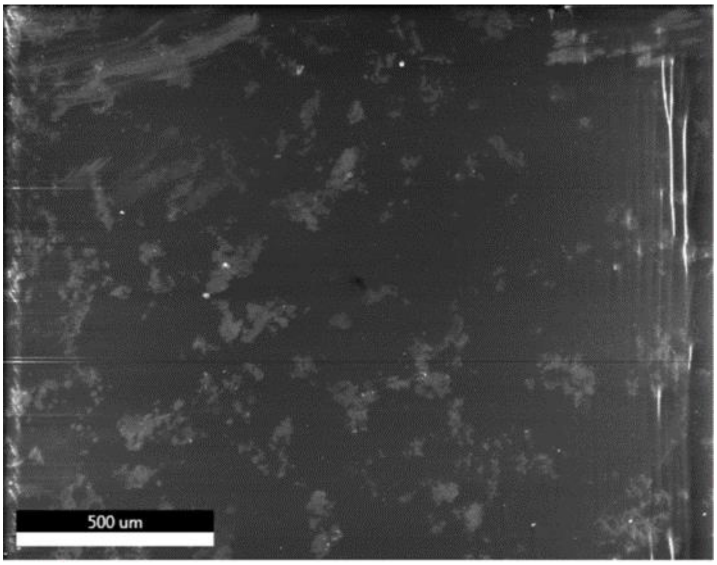

Figure S21. SEM micrograph of 1%-8GS sample.

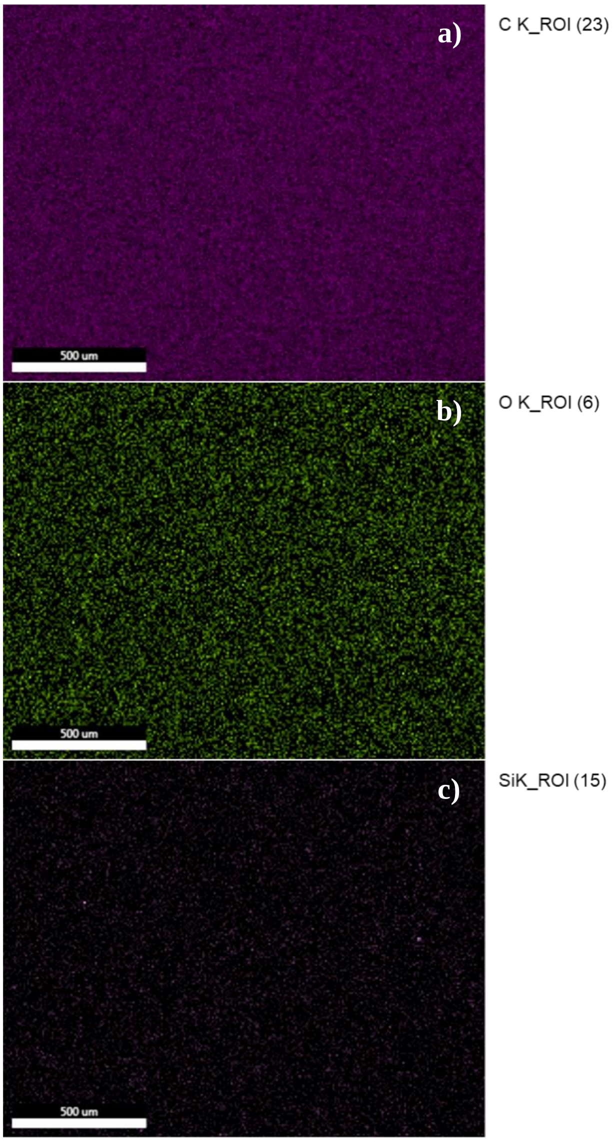

Figure S22. SEM-EDS a) carbon, b) oxygen, and c) silicon atoms distribution maps of 1%-8GS sample.

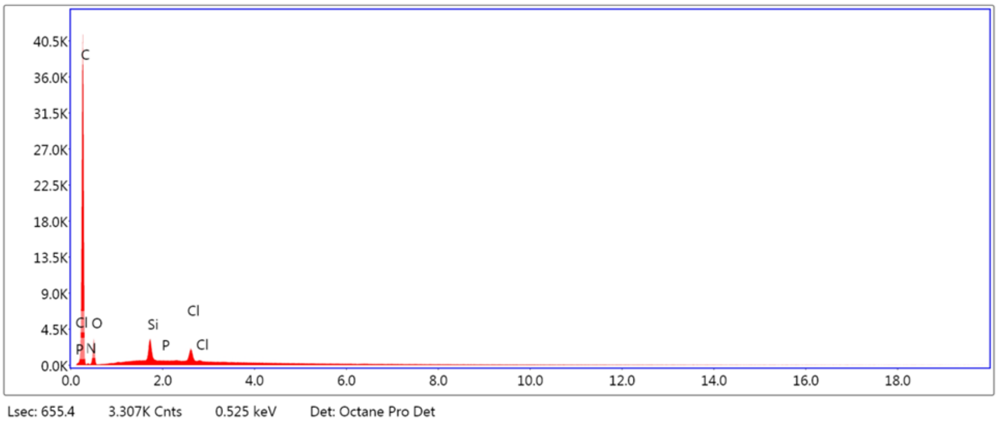

Figure S23. EDS spectrum of 5%-8GS sample.

Table S3. Results of EDS analysis of 5%-8GS sample.

Element	Weight %	Atomic %	Net Int.	Error %	Kratio	Z	R	A	F
C K	80.43	84.85	593.46	4.18	0.5334	1.0079	0.9953	0.6579	1.0000
N K	2.01	1.82	1.67	16.11	0.0013	0.9875	1.0052	0.0633	1.0000
O K	16.04	12.70	49.12	10.29	0.0146	0.9697	1.0142	0.0939	1.0000
SiK	0.91	0.41	62.52	3.59	0.0066	0.8913	1.0565	0.8088	1.0083
P K	0.01	0.00	0.67	56.14	0.0001	0.8572	1.0622	0.9054	1.0132
ClK	0.60	0.22	43.37	2.98	0.0053	0.8337	1.0729	1.0332	1.0238

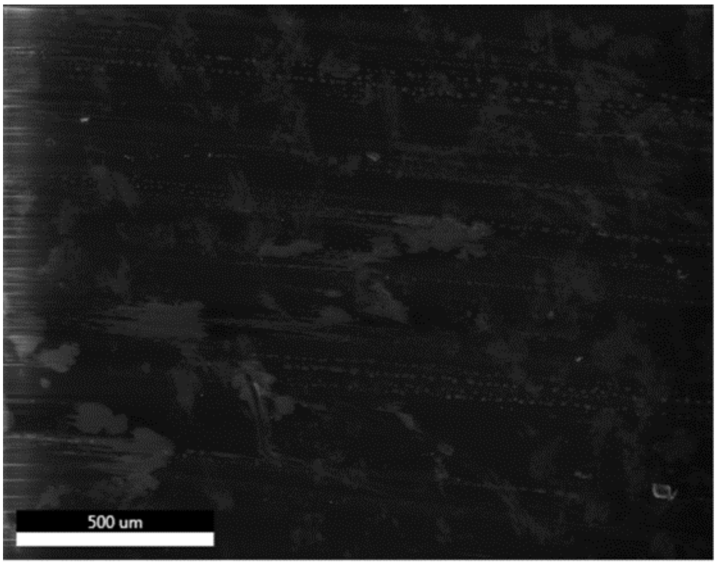

Figure S24. SEM micrograph of 5%-8GS sample.

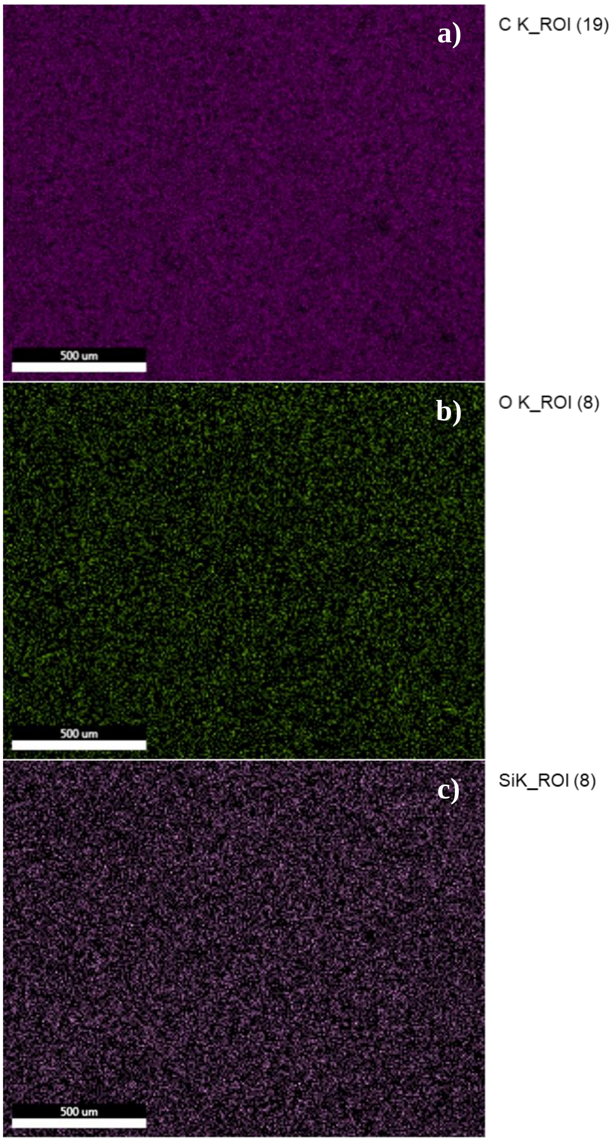

Figure S25. SEM-EDS a) carbon, b) oxygen, and c) silicon atoms distribution maps of 5%-8GS sample.

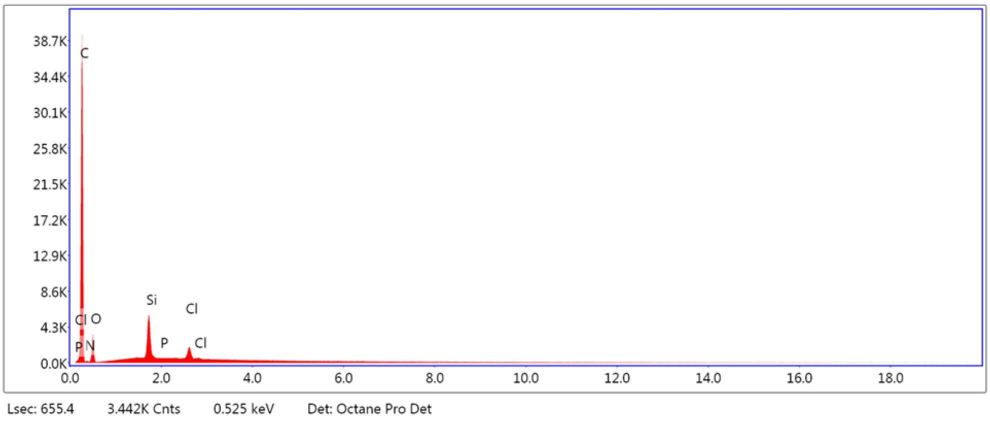

Figure S26. EDS spectrum of 10%-8GS sample.

Table S4. Results of EDS analysis of 10%-8GS sample.

Element	Weight %	Atomic %	Net Int.	Error %	Kratio	Z	R	A	F
C K	79.27	84.05	568.17	4.58	0.4946	1.0086	0.9947	0.6187	1.0000
N K	2.27	2.06	1.97	15.56	0.0014	0.9882	1.0046	0.0639	1.0000
O K	16.24	12.93	51.66	10.27	0.0149	0.9705	1.0137	0.0944	1.0000
SiK	1.69	0.77	120.40	3.13	0.0123	0.8920	1.0561	0.8080	1.0077
P K	0.01	0.01	0.96	55.94	0.0001	0.8580	1.0618	0.8873	1.0122
ClK	0.52	0.19	38.09	3.15	0.0045	0.8345	1.0725	1.0217	1.0228

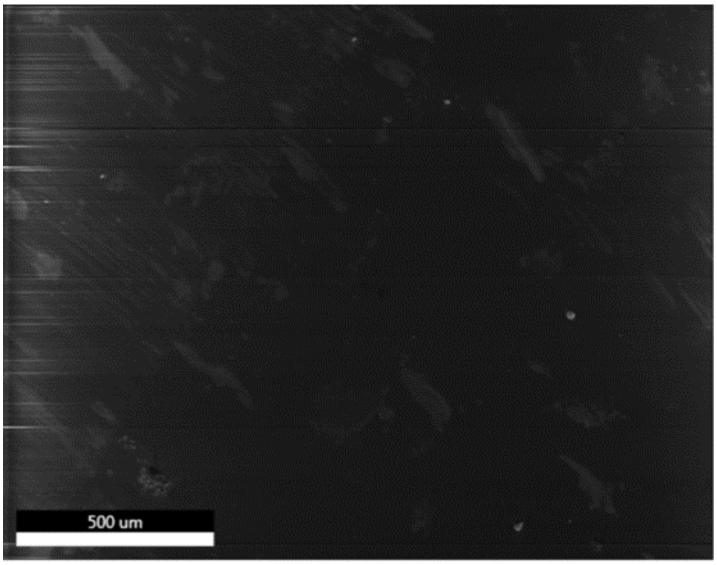

Figure S27. SEM micrograph of 10%-8GS sample.

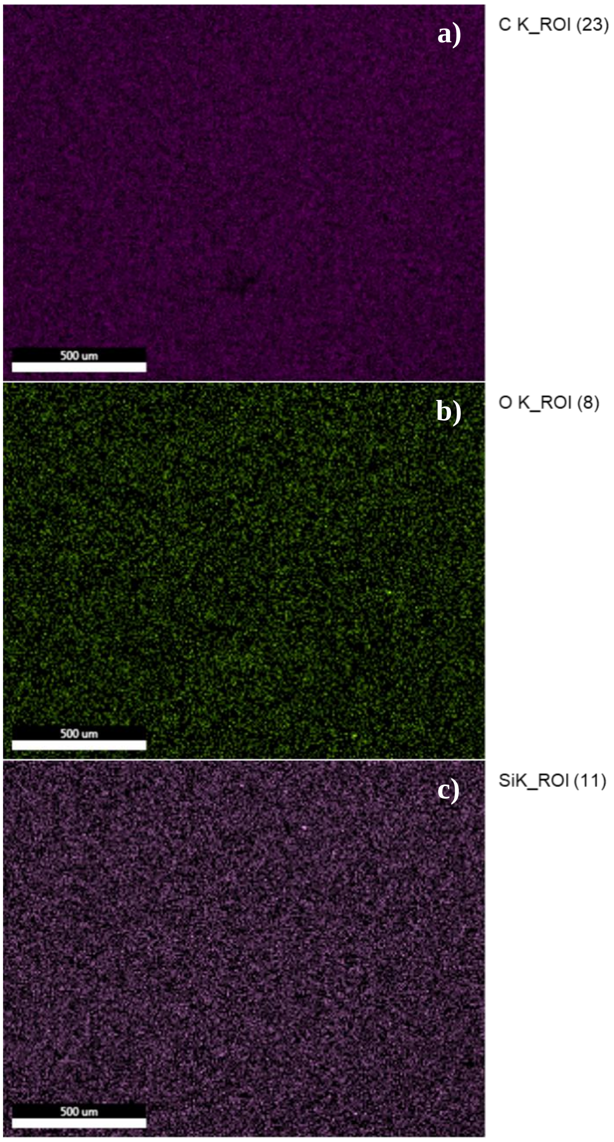

Figure S28. SEM-EDS a) carbon, b) oxygen, and c) silicon atoms distribution maps of 10%-8GS sample.

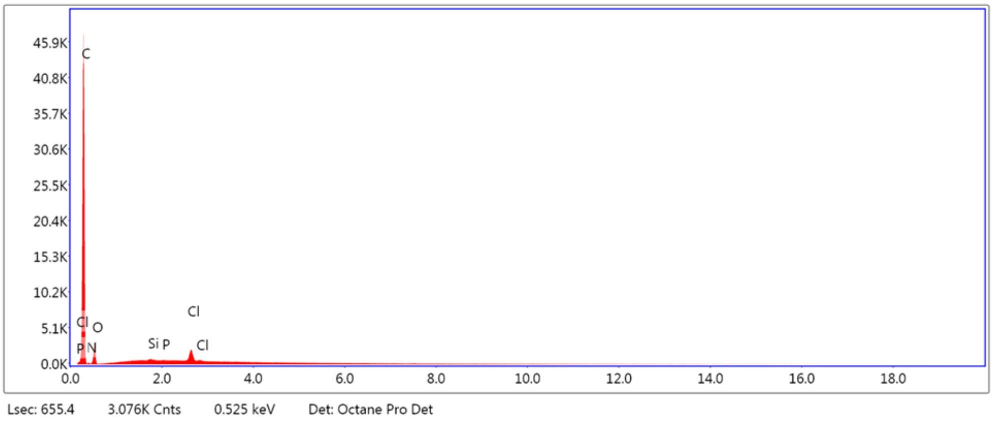

Figure S29. EDS spectrum of 1%-4P4GS sample.

Table S5. Results of EDS analysis of 1%-4P4GS sample.

Element	Weight %	Atomic %	Net Int.	Error %	Kratio	Z	R	A	F
C K	82.28	86.15	678.31	3.55	0.5967	1.0067	0.9961	0.7202	1.0000
N K	1.94	1.74	1.62	16.17	0.0012	0.9863	1.0060	0.0623	1.0000
O K	15.09	11.86	46.50	10.32	0.0135	0.9685	1.0149	0.0925	1.0000
SiK	0.07	0.03	4.93	18.71	0.0005	0.8901	1.0571	0.8123	1.0089
P K	0.02	0.01	1.42	54.88	0.0002	0.8561	1.0628	0.9275	1.0141
ClK	0.60	0.21	44.40	3.10	0.0053	0.8326	1.0735	1.0469	1.0254

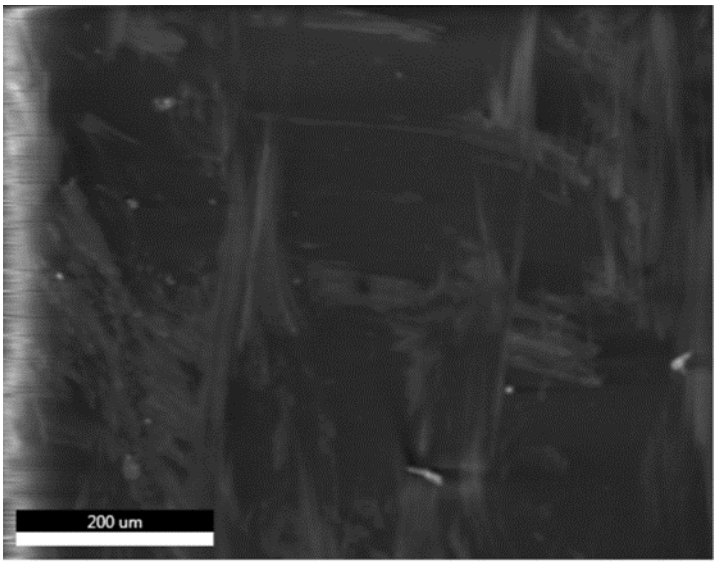

Figure S30. SEM micrograph of 1%-4P4GS sample.

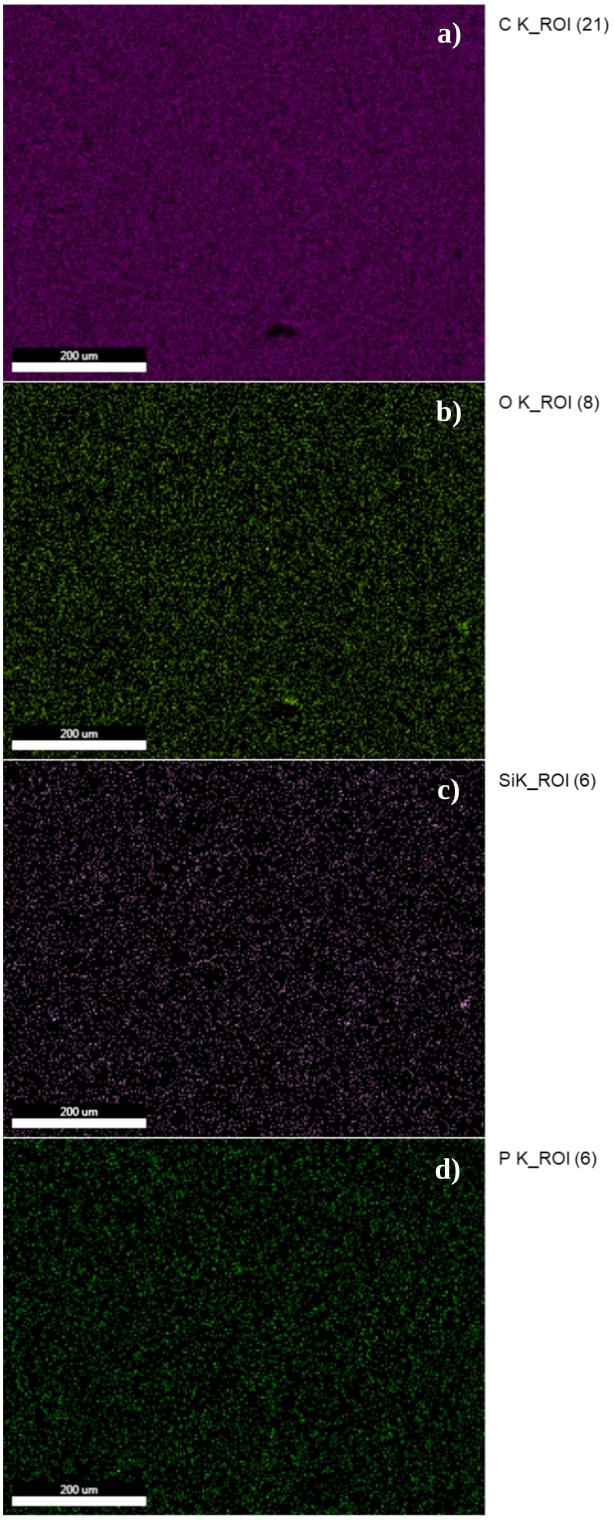

Figure S31. SEM-EDS a) carbon, b) oxygen, c) silicon and d) phosphorus atoms distribution maps of 1%-4P4GS sample.

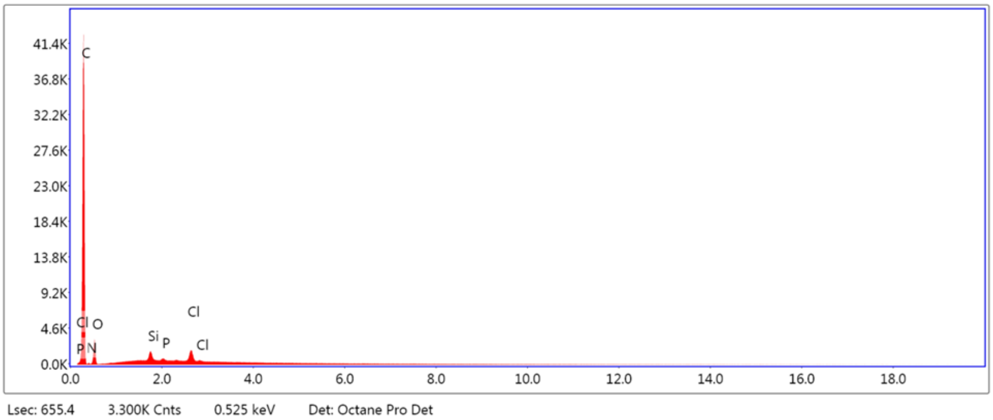

Figure S32. EDS spectrum of 5%-4P4GS sample.

Table S6. Results of EDS analysis of 5%-4P4GS sample.

Element	Weight %	Atomic %	Net Int.	Error %	Kratio	Z	R	A	F
C K	80.46	84.70	615.78	3.90	0.5566	1.0075	0.9956	0.6865	1.0000
N K	2.39	2.15	1.98	15.56	0.0015	0.9871	1.0055	0.0635	1.0000
O K	16.10	12.73	48.87	10.29	0.0146	0.9693	1.0145	0.0935	1.0000
SiK	0.40	0.18	27.17	4.86	0.0029	0.8909	1.0567	0.8075	1.0087
P K	0.10	0.04	6.32	12.84	0.0008	0.8569	1.0624	0.9160	1.0134
ClK	0.56	0.20	40.27	3.22	0.0050	0.8334	1.0731	1.0386	1.0246

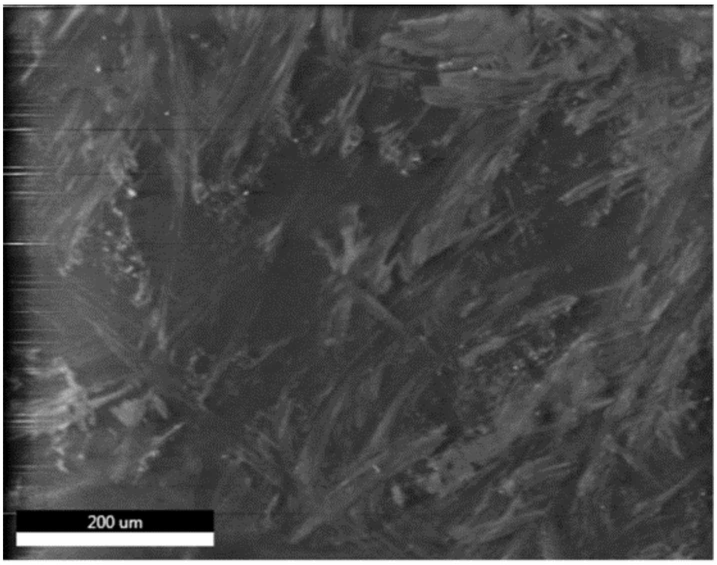

Figure S33. SEM micrograph of 5%-4P4GS sample.

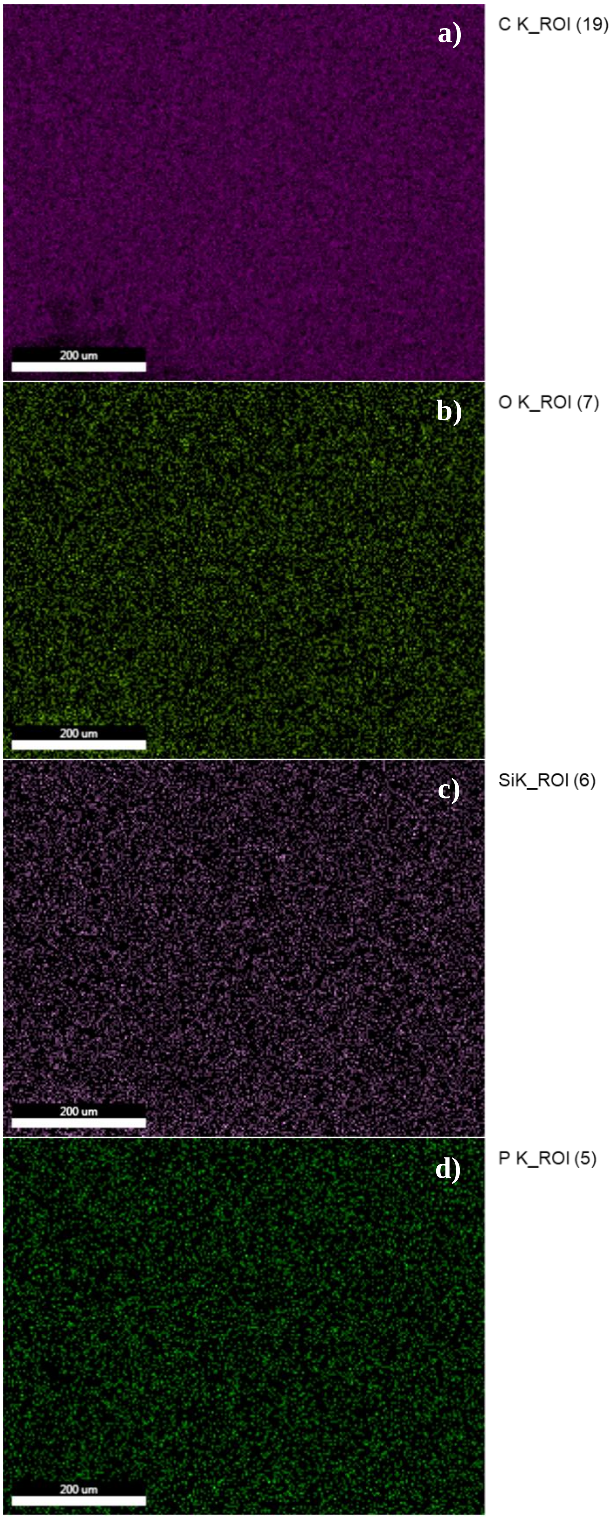

Figure S34. SEM-EDS a) carbon, b) oxygen, c) silicon and d) phosphorus atoms distribution maps of 5%-4P4GS sample.

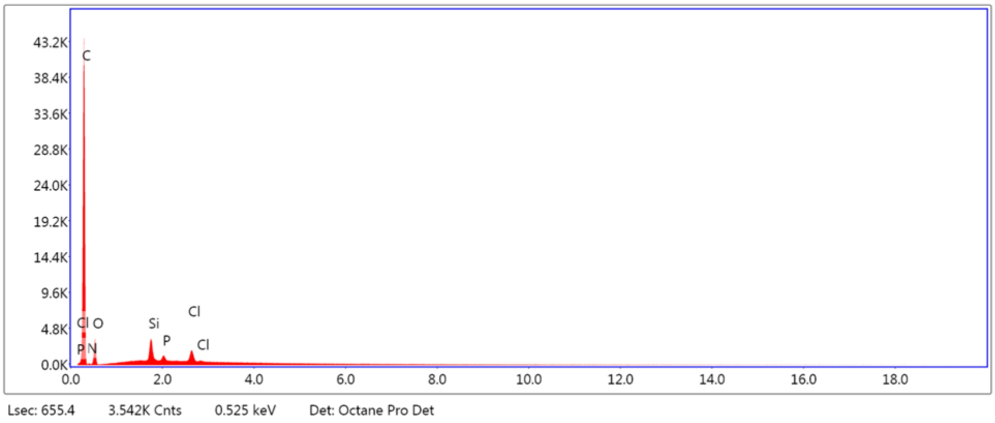

Figure S35. EDS spectrum of 10%-4P4GS sample.

Table S7. Results of EDS analysis of 10%-4P4GS sample.

Element	Weight %	Atomic %	Net Int.	Error %	Kratio	Z	R	A	F
C K	78.04	82.70	633.24	4.32	0.5068	1.0085	0.9949	0.6439	1.0000
N K	3.99	3.62	3.83	13.31	0.0026	0.9881	1.0048	0.0650	1.0000
O K	16.33	12.99	55.93	10.25	0.0148	0.9704	1.0139	0.0934	1.0000
SiK	0.88	0.40	67.49	3.48	0.0063	0.8919	1.0563	0.8037	1.0085
P K	0.25	0.10	18.39	6.61	0.0020	0.8579	1.0620	0.9021	1.0126
ClK	0.52	0.19	41.56	3.15	0.0045	0.8343	1.0727	1.0277	1.0235

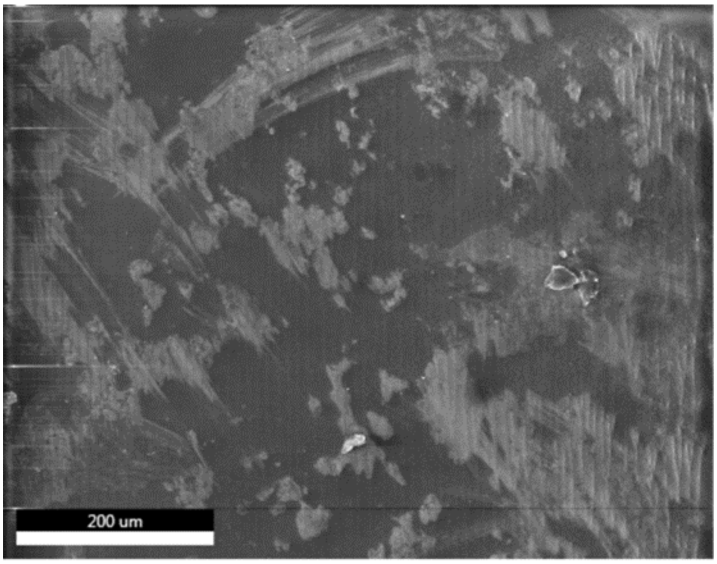

Figure S36. SEM micrograph of 10%-4P4GS sample.

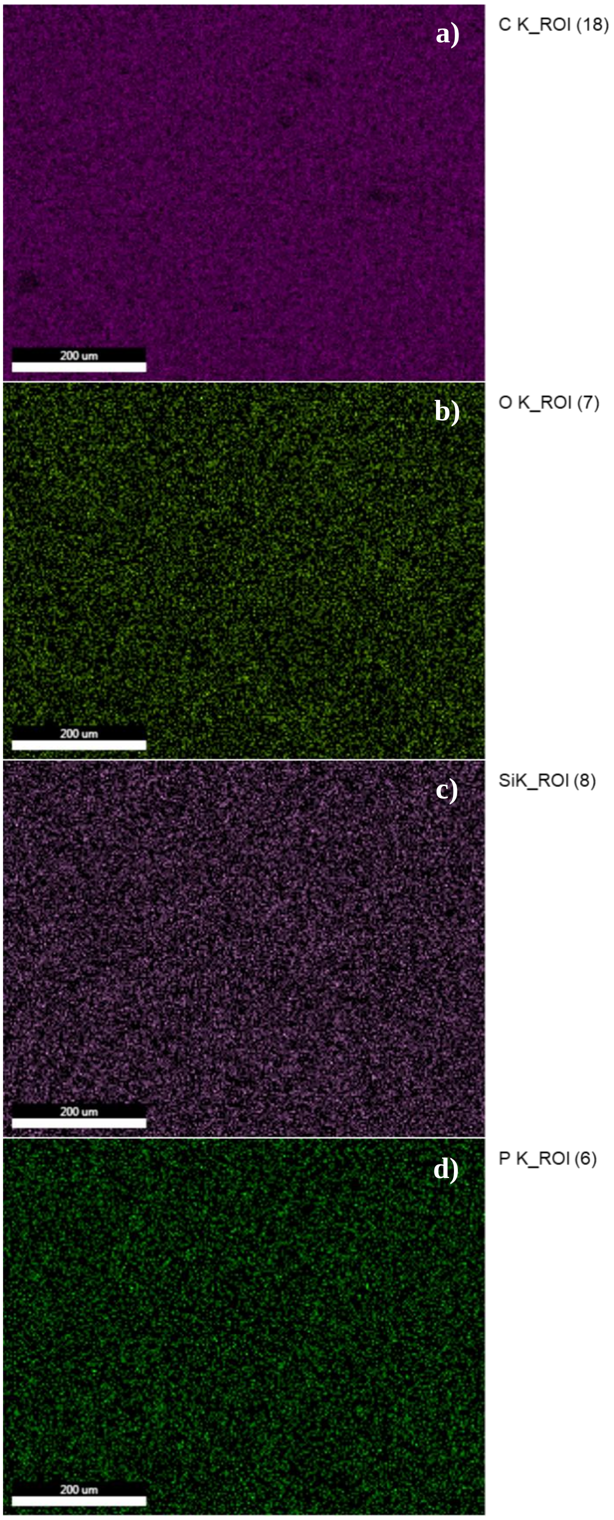

Figure S37. SEM-EDS a) carbon, b) oxygen, c) silicon and d) phosphorus atoms distribution maps of 10%-4P4GS sample.

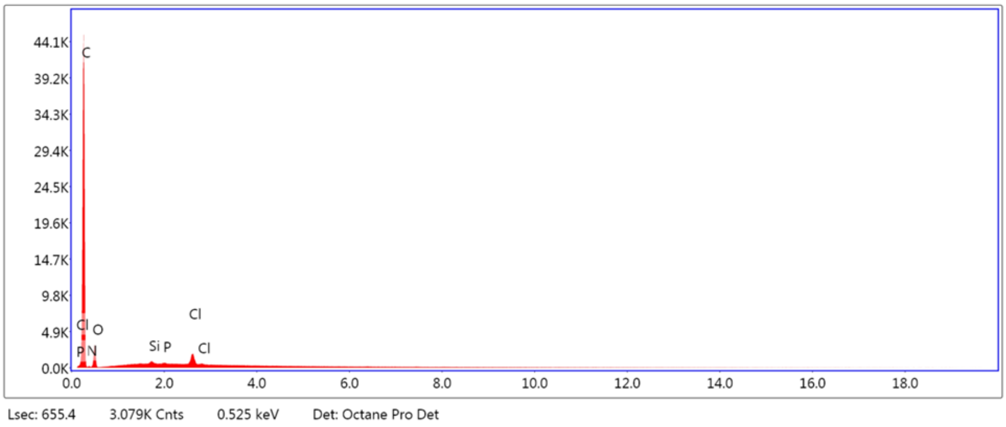

Figure S38. EDS spectrum of 1%-8PS sample.

Table S8. Results of EDS analysis of 1%-PS sample.

Element	Weight %	Atomic %	Net Int.	Error %	Kratio	Z	R	A	F
C K	81.75	85.70	652.21	3.63	0.5865	1.0069	0.9960	0.7124	1.0000
N K	2.20	1.98	1.81	15.80	0.0014	0.9865	1.0058	0.0626	1.0000
O K	15.28	12.03	46.13	10.32	0.0137	0.9687	1.0148	0.0926	1.0000
SiK	0.12	0.06	8.58	11.79	0.0009	0.8903	1.0570	0.8112	1.0089
P K	0.07	0.03	4.56	19.81	0.0006	0.8563	1.0627	0.9253	1.0139
ClK	0.58	0.20	41.75	3.18	0.0051	0.8328	1.0734	1.0447	1.0252

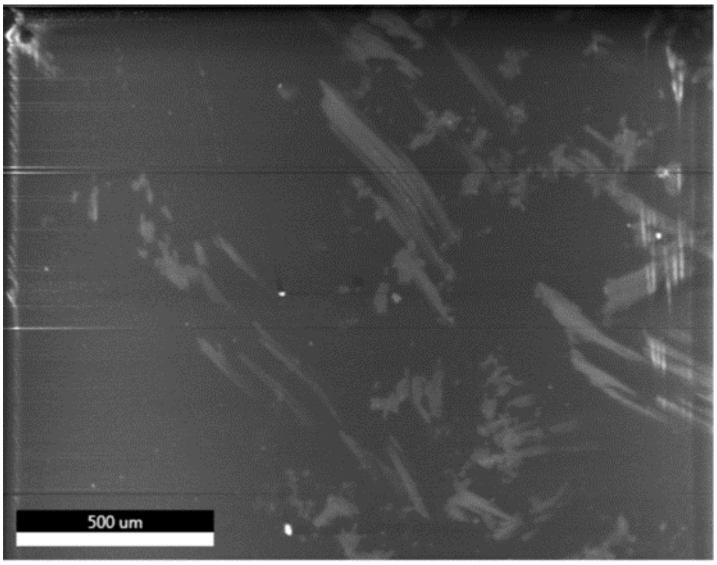

Figure S39. SEM micrograph of 1%-8PS sample.

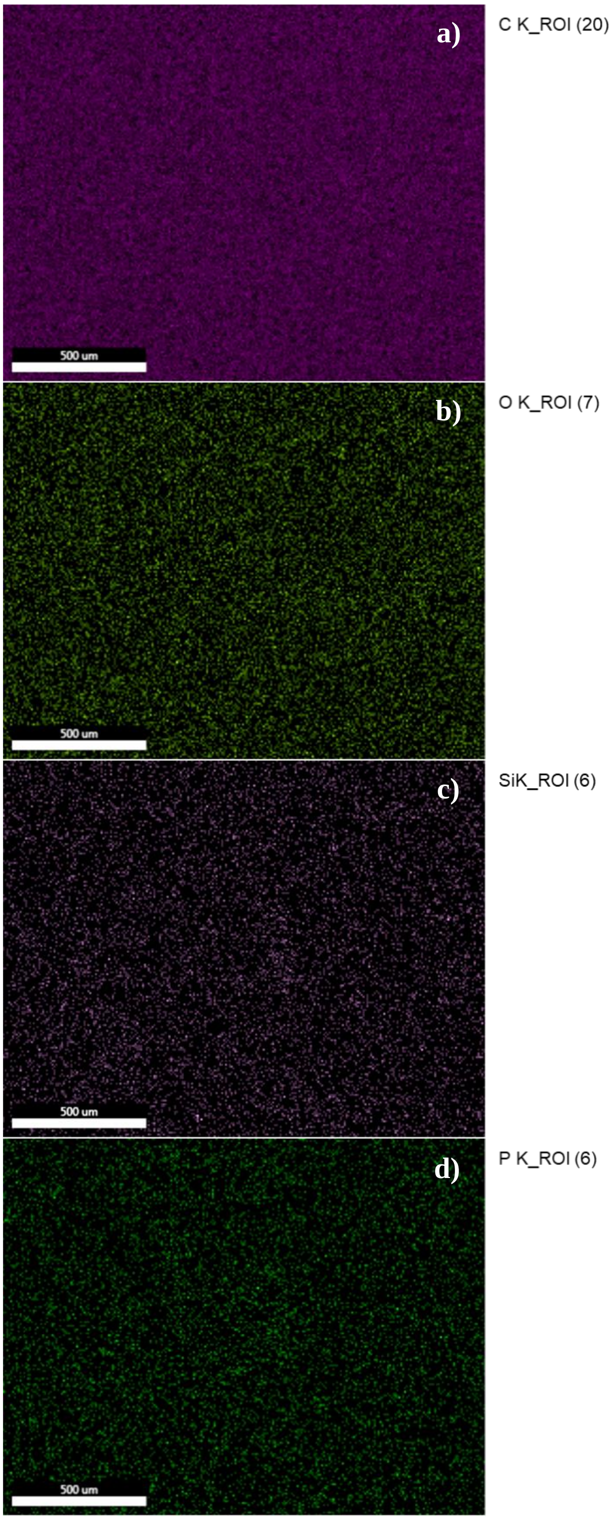

Figure S40. SEM-EDS a) carbon, b) oxygen, c) silicon and d) phosphorus atoms distribution maps of 1%-PS sample.

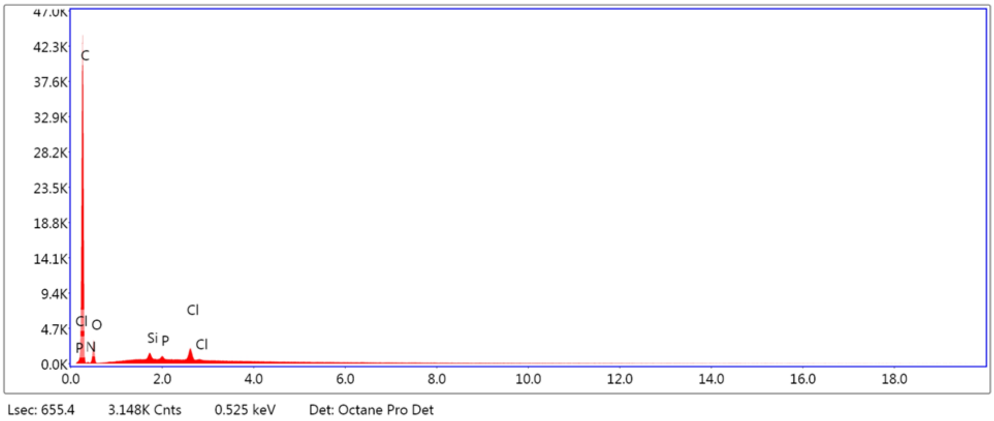

Figure S41. EDS spectrum of 5%-8PS sample.

Table S9. Results of EDS analysis of 5%-PS sample.

Element	Weight %	Atomic %	Net Int.	Error %	Kratio	Z	R	A	F
C K	81.30	85.42	631.42	3.86	0.5647	1.0073	0.9957	0.6895	1.0000
N K	2.32	2.09	1.93	15.65	0.0014	0.9869	1.0056	0.0628	1.0000
O K	15.31	12.08	46.53	10.32	0.0138	0.9691	1.0146	0.0927	1.0000
SiK	0.30	0.14	20.98	5.21	0.0022	0.8906	1.0568	0.8107	1.0090
P K	0.17	0.07	11.66	8.55	0.0014	0.8566	1.0625	0.9209	1.0136
ClK	0.59	0.21	42.94	3.16	0.0052	0.8331	1.0732	1.0405	1.0246

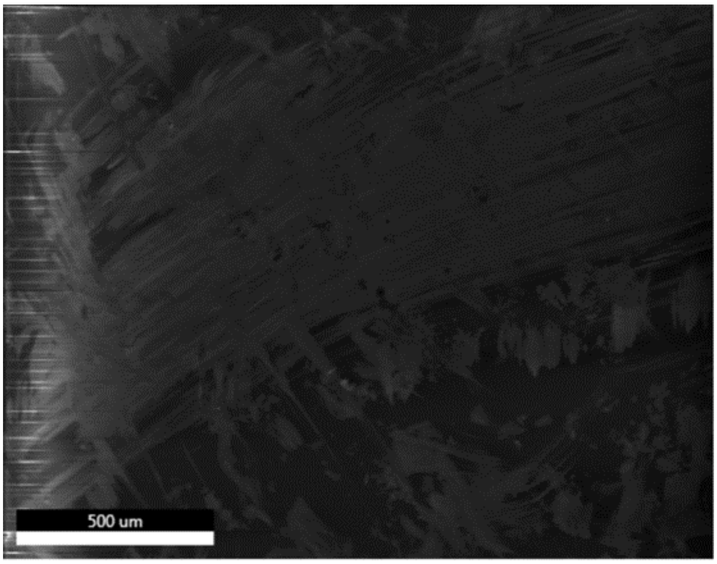

Figure S42. SEM micrograph of 5%-8PS sample.

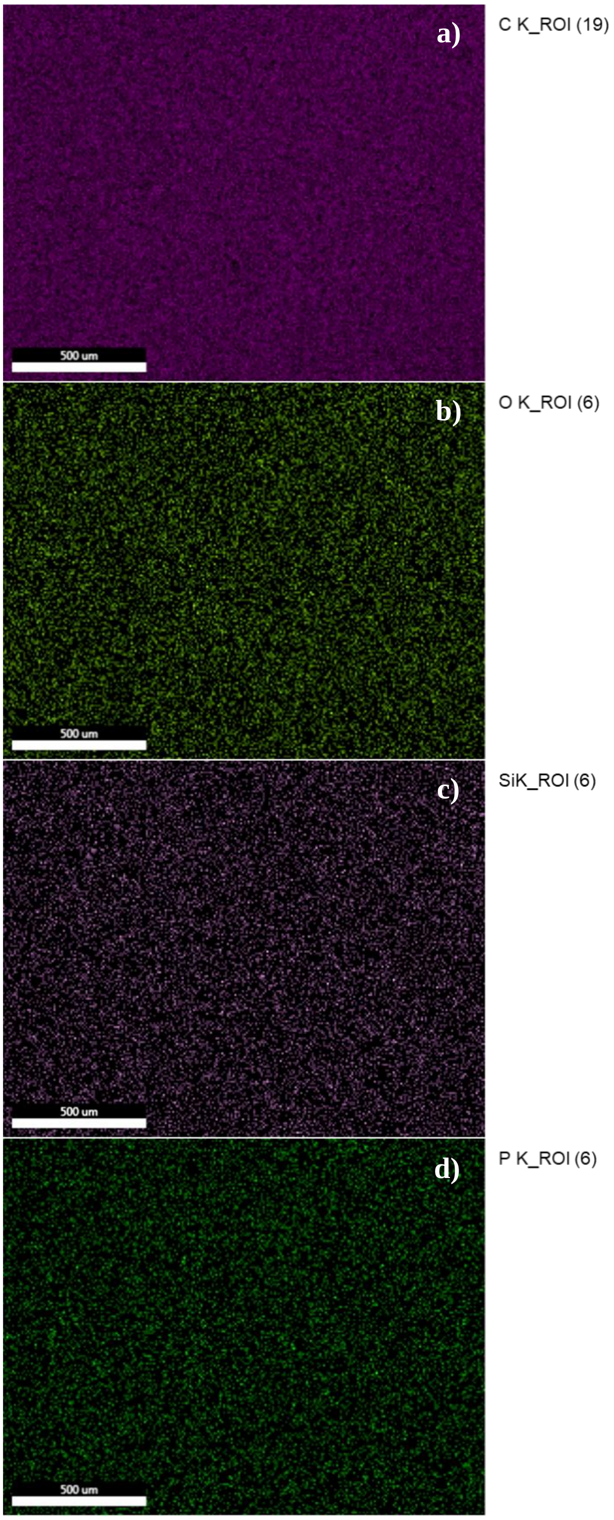

Figure S43. SEM-EDS a) carbon, b) oxygen, c) silicon and d) phosphorus atoms distribution maps of 5%-PS sample.

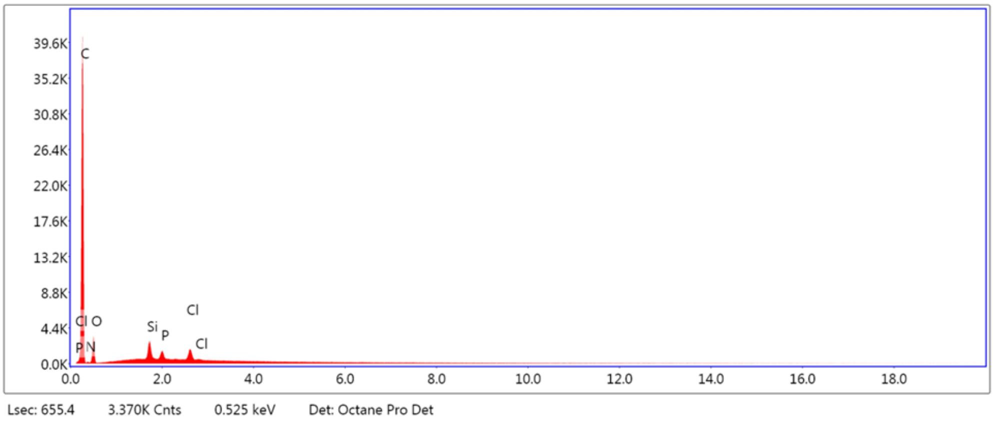

Figure S44. EDS spectrum of 10%-8PS sample.

Table S10. Results of EDS analysis of 10%-PS sample.

Element	Weight %	Atomic %	Net Int.	Error %	Kratio	Z	R	A	F
C K	80.52	85.01	583.24	4.27	0.5273	1.0080	0.9952	0.6496	1.0000
N K	1.55	1.40	1.28	17.90	0.0010	0.9876	1.0051	0.0632	1.0000
O K	16.28	12.91	49.88	10.28	0.0149	0.9699	1.0141	0.0944	1.0000
SiK	0.73	0.33	49.88	3.68	0.0053	0.8914	1.0564	0.8084	1.0089
P K	0.41	0.17	26.39	4.99	0.0032	0.8574	1.0622	0.9092	1.0126
ClK	0.51	0.18	36.31	3.38	0.0045	0.8339	1.0729	1.0297	1.0236

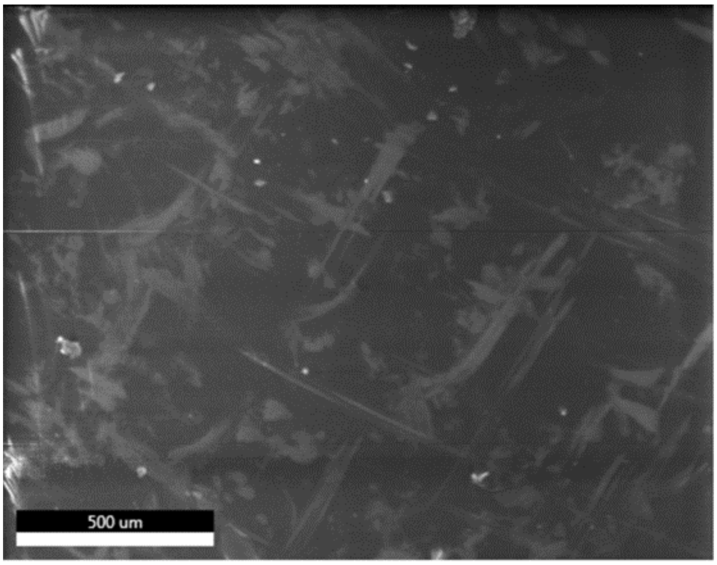

Figure S45. SEM micrograph of 10%-8PS sample.

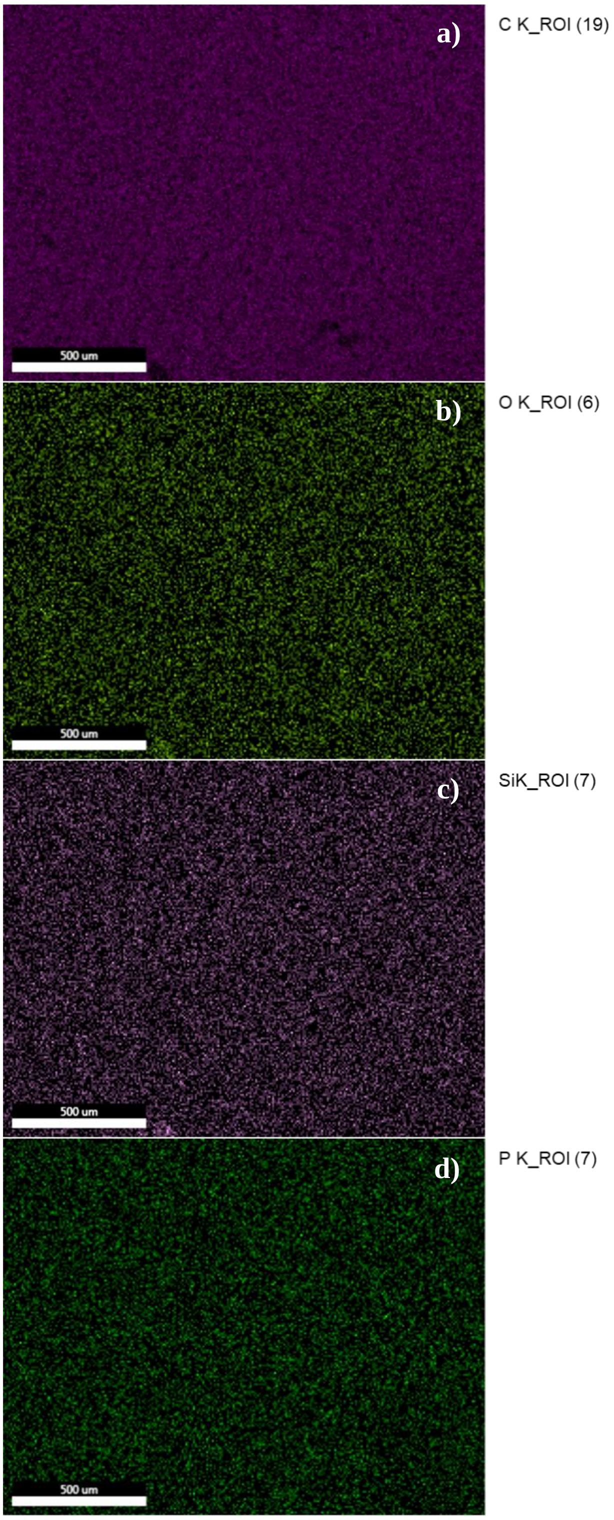

Figure S46. SEM-EDS a) carbon, b) oxygen, c) silicon and d) phosphorus atoms distribution maps of 10%-PS sample.

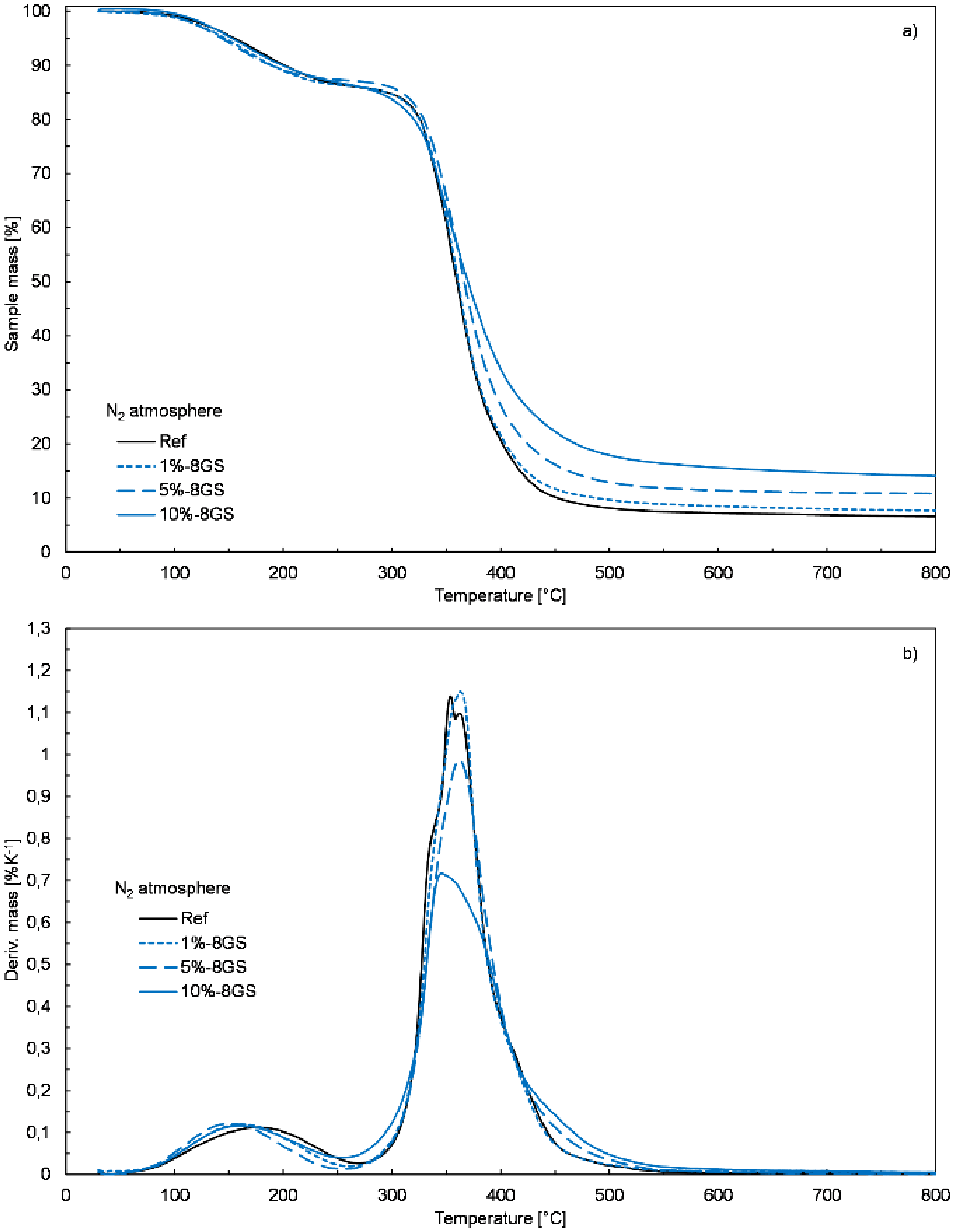

Figure S47. Graphs of a) TG and b) DTG curves of epoxy resin samples modified with 1, 5 and 10%wt. of 8GS derivative in N_2 atmosphere.

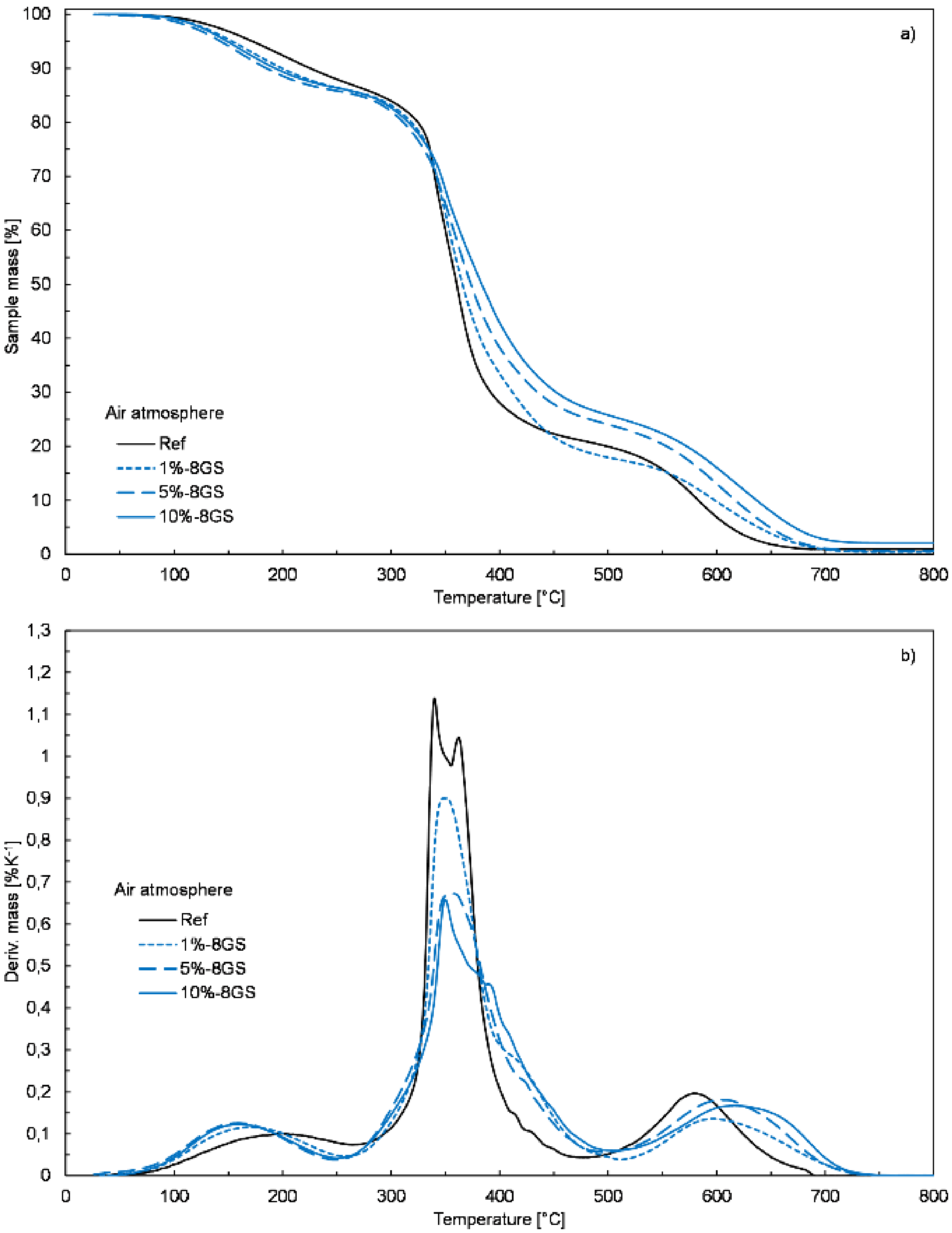

Figure S48. Graphs of a)TG and b) DTG curves of epoxy resin samples modified with 1, 5 and 10% wt. of 8GS derivative in air atmosphere.

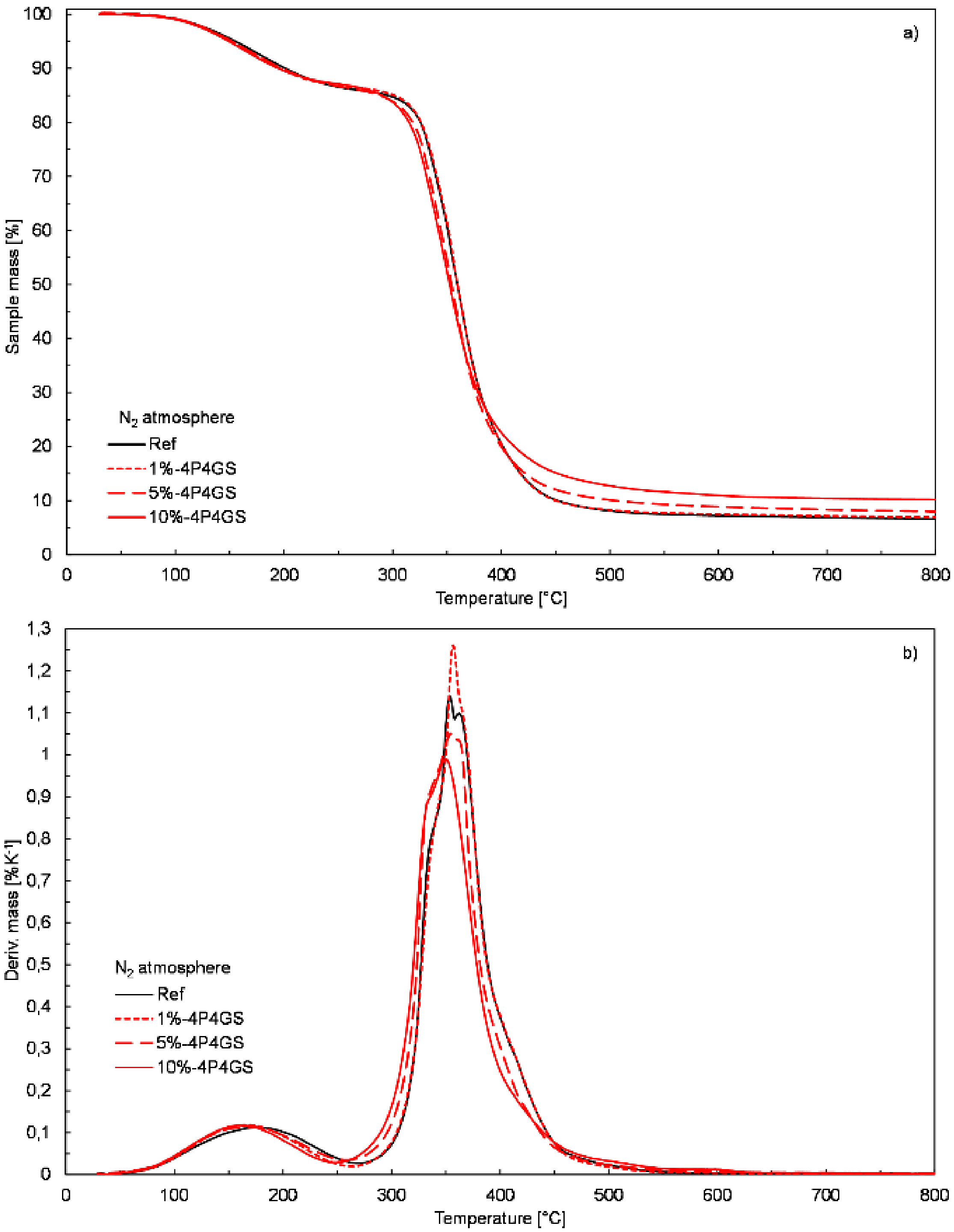

Figure S49. Graphs of a) TG and b) DTG curves of epoxy resin samples modified with 1, 5 and 10% wt. of 4P4GS derivative in N_2 atmosphere.

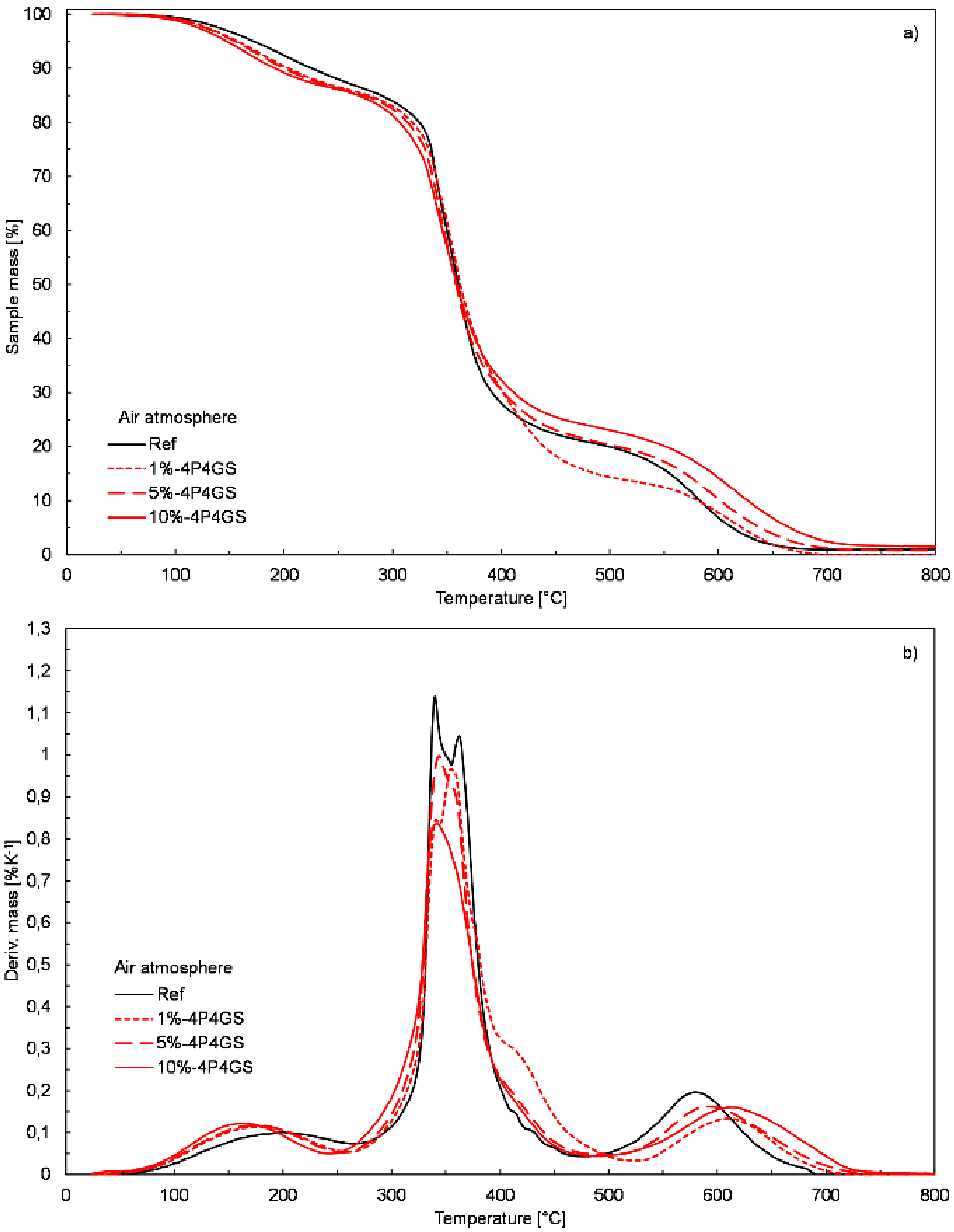

Figure S50. Graphs of a) TG and b) DTG curves of epoxy resin samples modified with 1, 5 and 10% of 4P4GS derivative in air atmosphere.

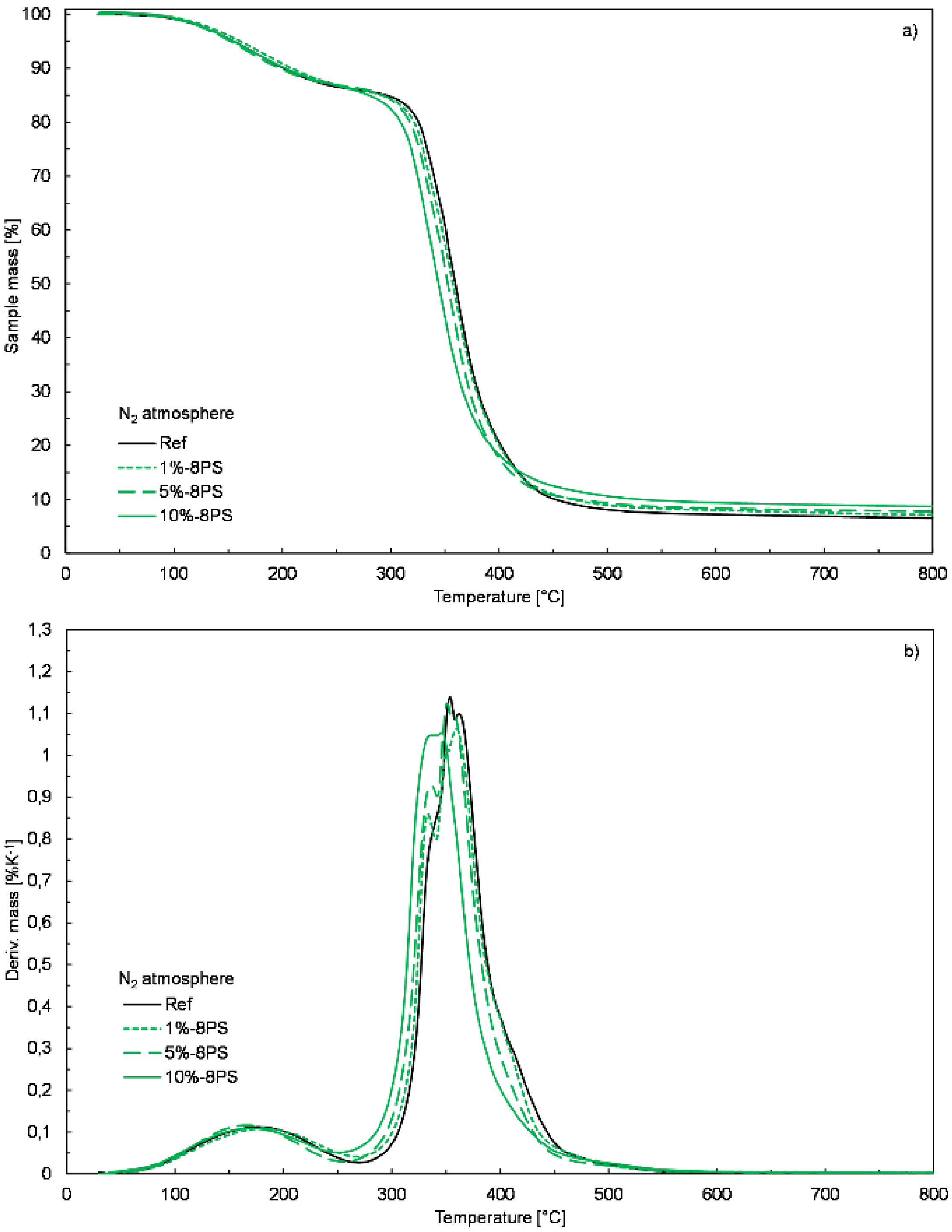

Figure S51. Graphs of a) TG and b) DTG curves of epoxy resin samples modified with 1, 5 and 10% wt. of 8PS derivative in N_2 atmosphere.

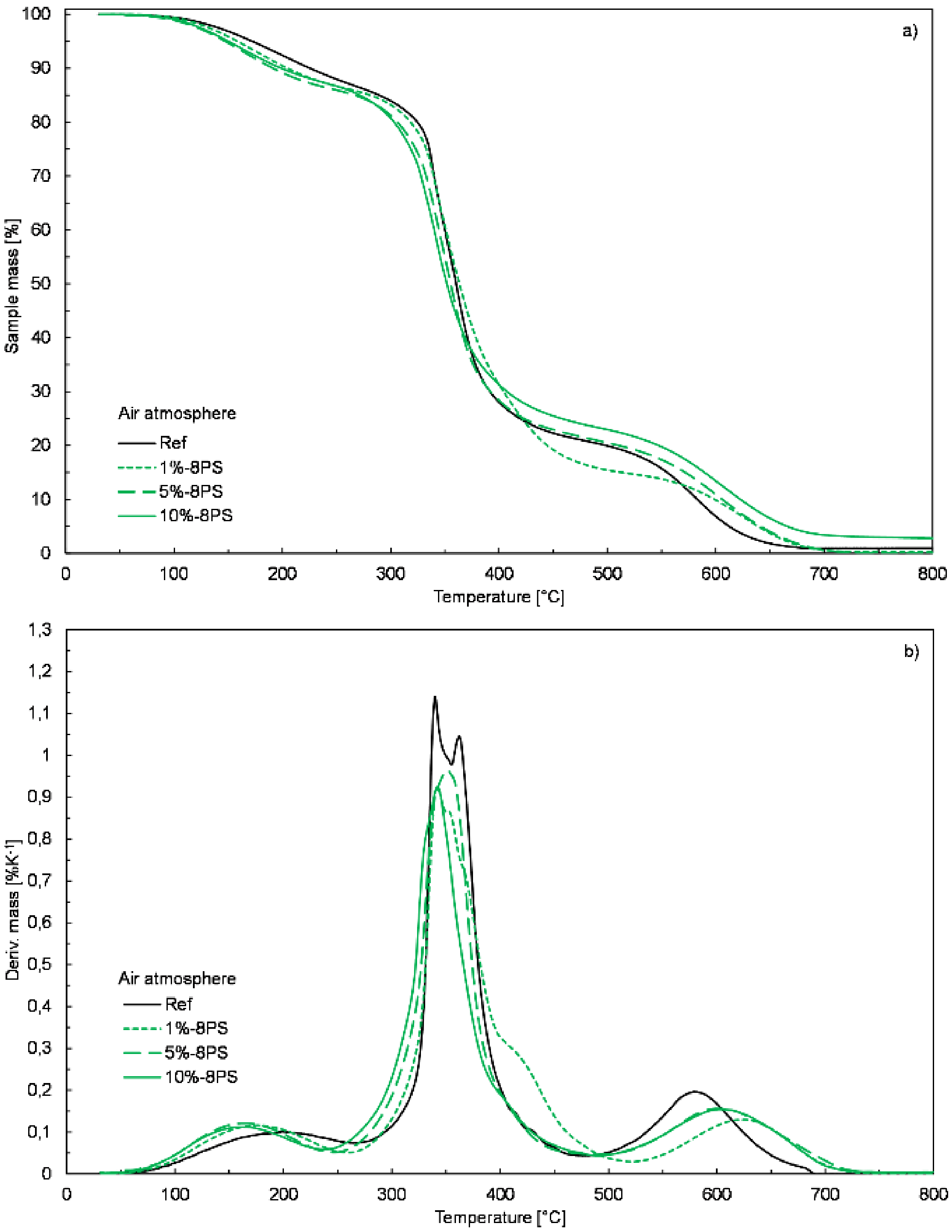

Figure S52. Graphs of a) TG and b) DTG curves of epoxy resin samples modified with 1, 5 and 10% wt. of 8PS derivative in air atmosphere.

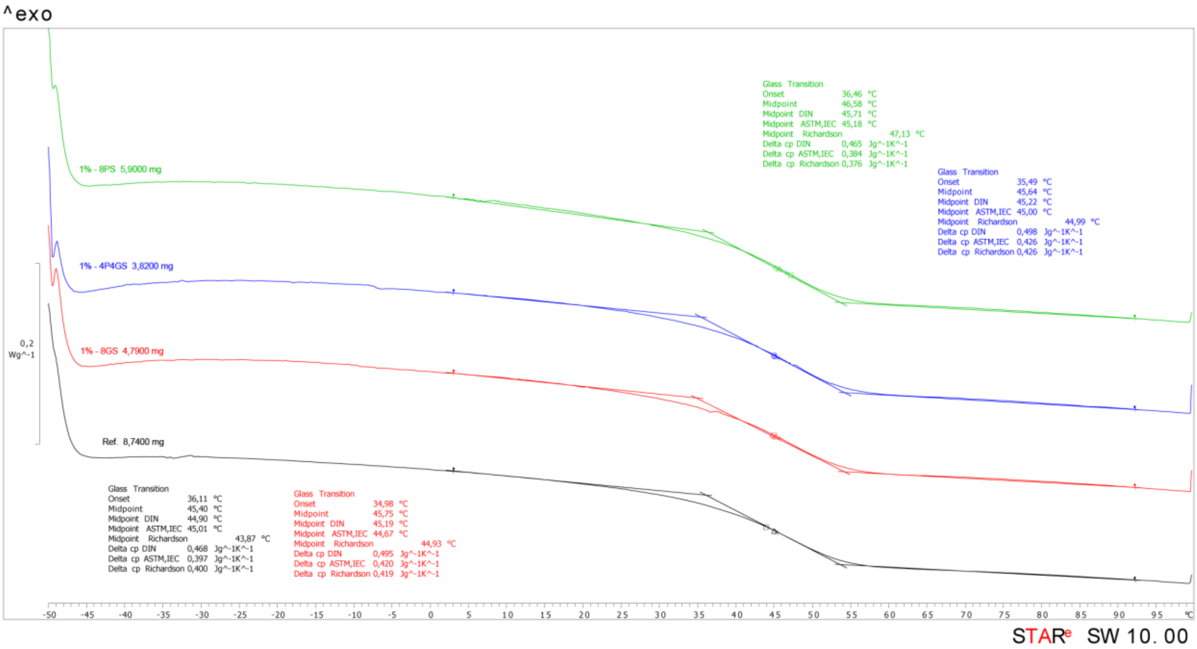

Figure S53. DSC heating curves for epoxy resins modified with 1%wt. of 8GS, 4P4GS and 8PS derivatives

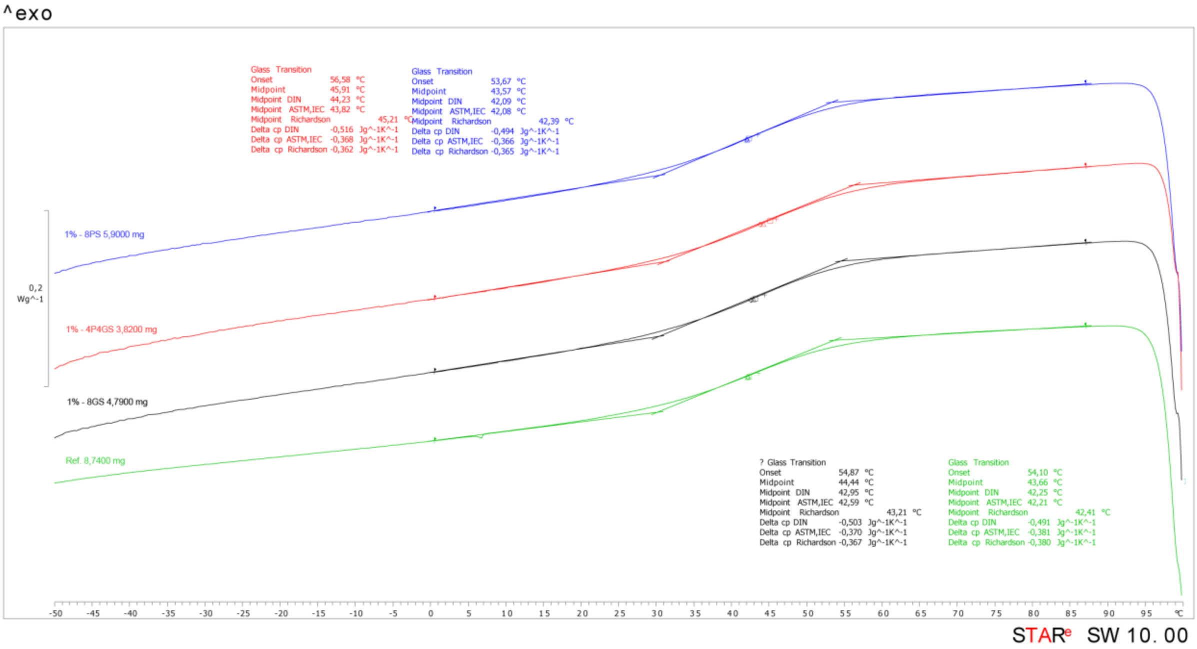

Figure S54. DSC cooling curves for epoxy resins modified with 1%wt. of 8GS, 4P4GS and 8PS derivatives

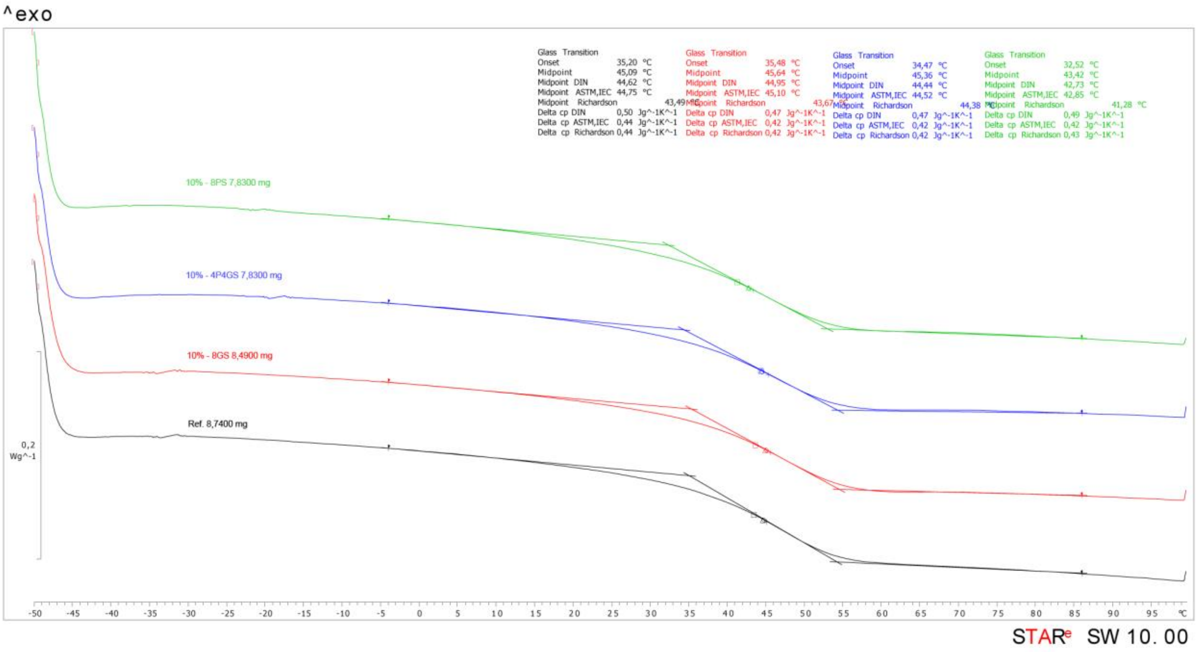

Figure S55. DSC heating curves for epoxy resins modified with 10%wt. of 8GS, 4P4GS and 8PS derivatives

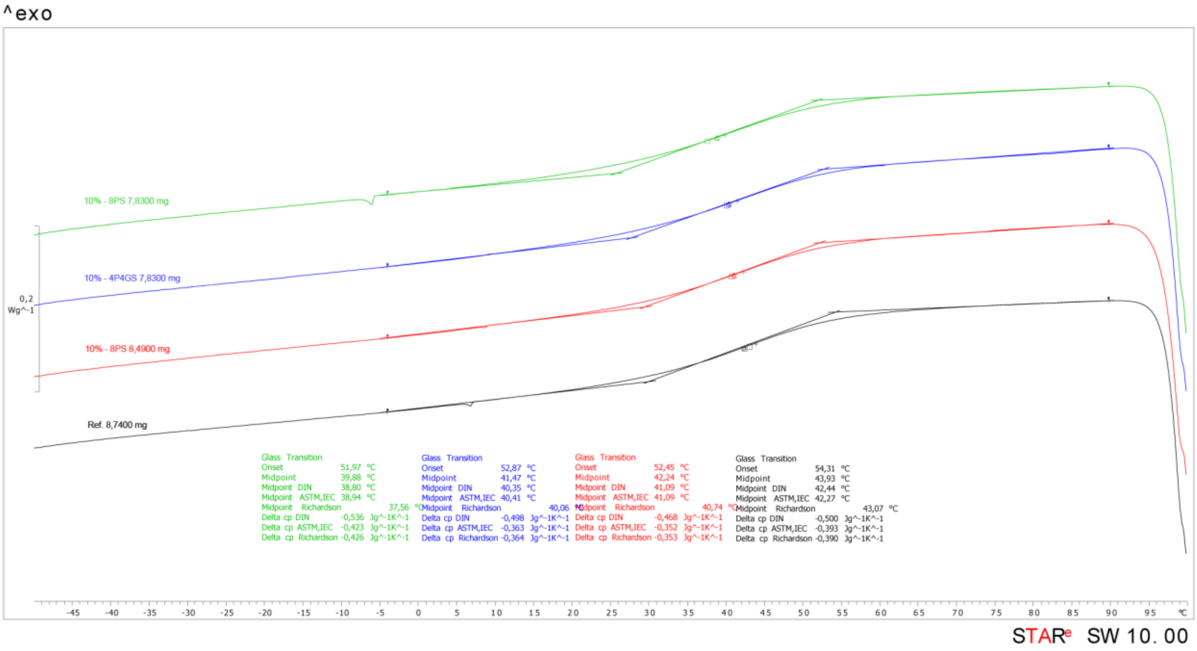

Figure S56. DSC cooling curves for epoxy resins modified with 10%wt. of 8GS, 4P4GS and 8PS derivatives
